# Multiple horizontal transfer events of a DNA transposon into turtles, fishes, and a frog

**DOI:** 10.1186/s13100-024-00318-9

**Published:** 2024-04-11

**Authors:** Nozhat T. Hassan, James D. Galbraith, David L. Adelson

**Affiliations:** 1 School of Biological Sciences, University of Adelaide, Adelaide, Australia; 2 Institute of Ecology and Evolution, University of Edinburgh, Edinburgh, UK

**Keywords:** Horizontal transfer, Transposable element, DNA transposable element, Genome evolution, Vertebrate

## Abstract

Horizontal transfer of transposable elements (HTT) has been reported across many species and the impact of such events on genome structure and function has been well described. However, few studies have focused on reptilian genomes, especially HTT events in Testudines (turtles). Here, as a consequence of investigating the repetitive content of *Malaclemys terrapin terrapin* (Diamondback turtle) we found a high similarity DNA transposon, annotated in RepBase as hAT-6_XT, shared between other turtle species, ray-finned fishes, and a frog. hAT-6_XT was notably absent in reptilian taxa closely related to turtles, such as crocodiles and birds. Successful invasion of DNA transposons into new genomes requires the conservation of specific residues in the encoded transposase, and through structural analysis, these residues were identified indicating some retention of functional transposition activity. We document six recent independent HTT events of a DNA transposon in turtles, which are known to have a low genomic evolutionary rate and ancient repeats.

**Supplementary Information:**

The online version contains supplementary material available at 10.1186/s13100-024-00318-9.

## Summary of species

*Malaclemys terrapin terrapin* (Diamondback turtle).

*Malaclemys terrapin pileata* (Mississippi diamondback terrapin turtle).

*Trachemys scripta elegans* (Red-eared slider turtle).

*Chrysemys picta bellii* (Western painted turtle).

*Dermatemys mawii* (Hickatee turtle).

*Sternotherus odoratus* (Common musk turtle).

*Mesoclemmys tuberculata* (Tuberculate Toad-headed turtle).

*Etheostoma spectabile* (Orangethroat darter fish).

*Thalassophryne amazonica* (Prehistoric monster fish).

*Scophthalmus maximus* (Turbot fish).

*Syngnathus acus* (Greater pipefish).

*Scleropages formosus* (Asian Arowana fish).

*Xenopus tropicalis* (Western clawed frog).

**Supplementary Information:**

The online version contains supplementary material available at 10.1186/s13100-024-00318-9.

## Introduction

A large proportion of eukaryotic genomes is composed of mobile repetitive sequences known as transposable elements (TEs). TEs are divided into two classes based on their mode of mobilisation. Retrotransposons (Class I) copy themselves using an RNA intermediate which is reverse-transcribed into cDNA and is integrated back into the host genome, whereas DNA transposons (Class II) move via a cut-and-paste mechanism mediated by an encoded transposase [1, 2]. One of the largest and most widespread superfamilies of DNA transposons are hAT elements. While there is sequence variation between families of hAT transposons, they are defined by ∽ 8 bp terminal target site duplication (TSDs) and ∽ 15 bp terminal inverted repeats (TIRs) [3]. The hAT transposase is a multidomain protein and the only hAT transposase crystal structure is from the Hermes transposase of the house fly (*Musca domestica*), which showed the presence of conserved residues and domains required for TIR recognition and transposition [4, 5].

Testudines (turtles) are a group of reptiles found in diverse ecological settings ranging from terrestrial, marine, and freshwater environments and are a sister group to Archosauria (birds and crocodilians) that diverged during the Permian-Triassic period approximately 257.4 Mya [6, 7]. The repetitive content of turtle genomes has not been studied extensively, but current studies indicate that TEs make up approximately 30% of the genome in various turtles and are dominated by LINEs and DNA transposons [8–10]. Turtle genomes generally do not show significant variation in size and appear to evolve slowly compared to other reptiles which makes analysis of repeats an area of interest [11]. Until now, horizontal transfer of DNA transposable elements (HTT) has not been documented in or between turtles, fishes, and a frog.

Horizontal transfer is the process by which genetic material is obtained from non-parental genomes/sources, as opposed to vertical transfer which is from parent to offspring [12, 13]. TEs, in particular, are widely spread through horizontal transfer in eukaryotes and can persist within the invaded genome [14]. HTT has been documented in several species, for example, SPIN (DNA TEs) and BovB elements (non-LTR retrotransposons) have colonised many squamate reptiles [15–17]. However, in both studies, evidence for HTT was notably absent in turtles. In Zhang et al. (2020), HTT of both retrotransposons and DNA transposons between turtles, fishes, and lizards were inferred, however, they did not specifically detect hAT-6_XT (first curated in *Xenopus tropicalis* by Kapitonov & Jurka, 2006) HTT events between species, HTT events between turtles or carry out an evolutionary analysis [18, 19]. In this study, we outline a rare horizontal transfer of a hAT-6_XT DNA TE between/into six turtles, fishes, and a frog.

## Results and discussion

### HTT amongst species of turtles, fishes, and a frog

Horizontal transposon transfer has been widely reported in the species discussed in this study, with new reports emerging describing HTT of both DNA and RNA transposable elements in ray-finned fish, amphibians, and reptiles [18, 20]. HTT between turtles, especially of DNA transposons, has been proposed to be a rare event based on our current understanding of turtle genome evolution [18, 20, 21]. We screened the genomes of approximately 100 species ranging from fishes, reptiles, mammals, birds, and insects to find hAT-6_XTs that share striking homology (Additional file 1; Table S1). We have identified the first case of horizontal transfer of a hAT-6_XT DNA transposon, first annotated in *Xenopus tropicals*, into turtles, ray-finned fishes, and a frog.

**Fig. 1:**
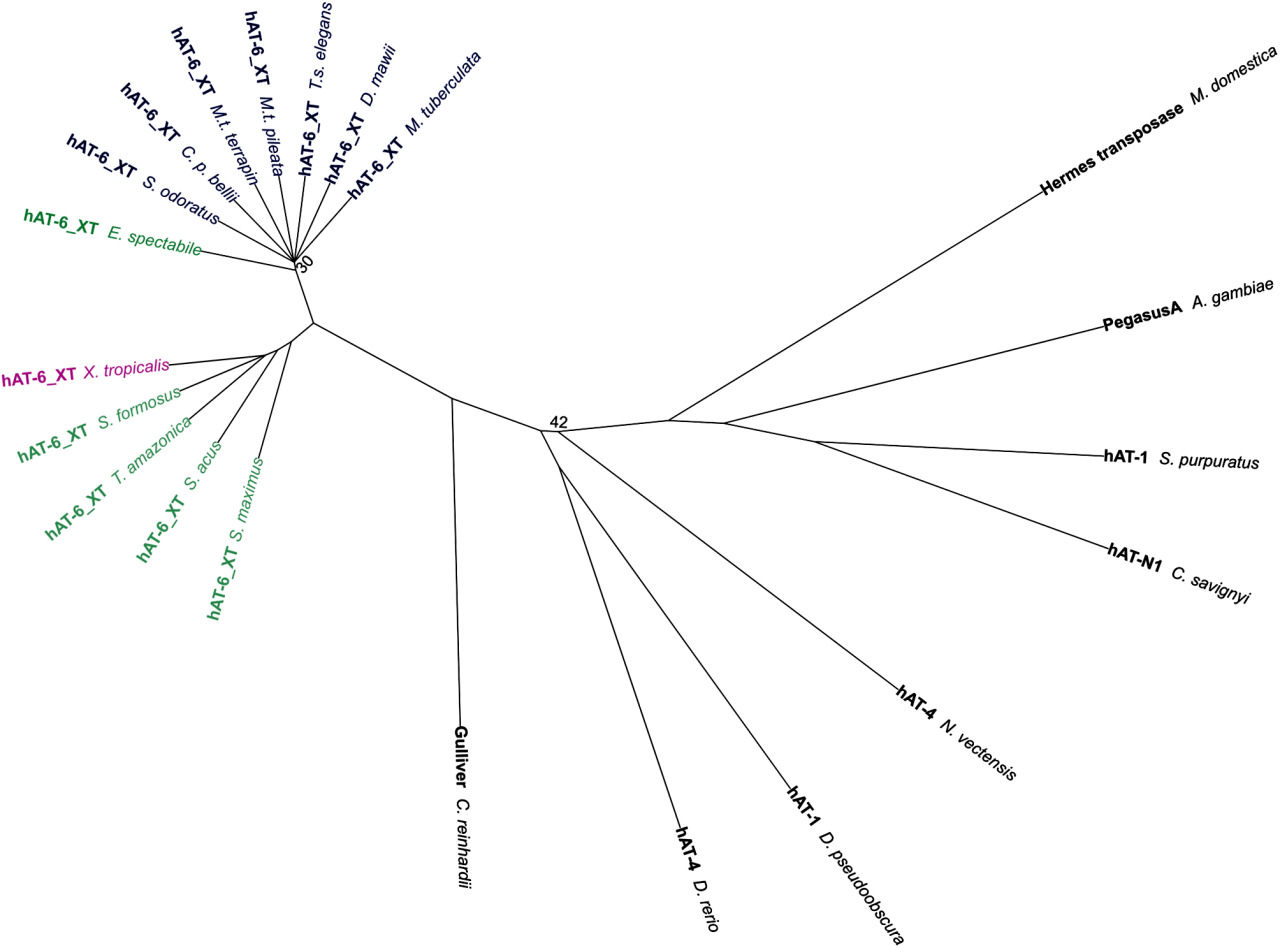
Phylogeny of horizontally transferred hAT-6_XT TEs with a sample of other hAT TEs from Repbase. Tree constructed using IQTree 2 (1000 bootstraps) based on MAFFT protein alignments trimmed using Clipkit and formatted in iTOL. Support values under 65 are displayed at nodes. Turtle species are in blue, frogs are in pink, and fishes are in green

**Fig. 2:**
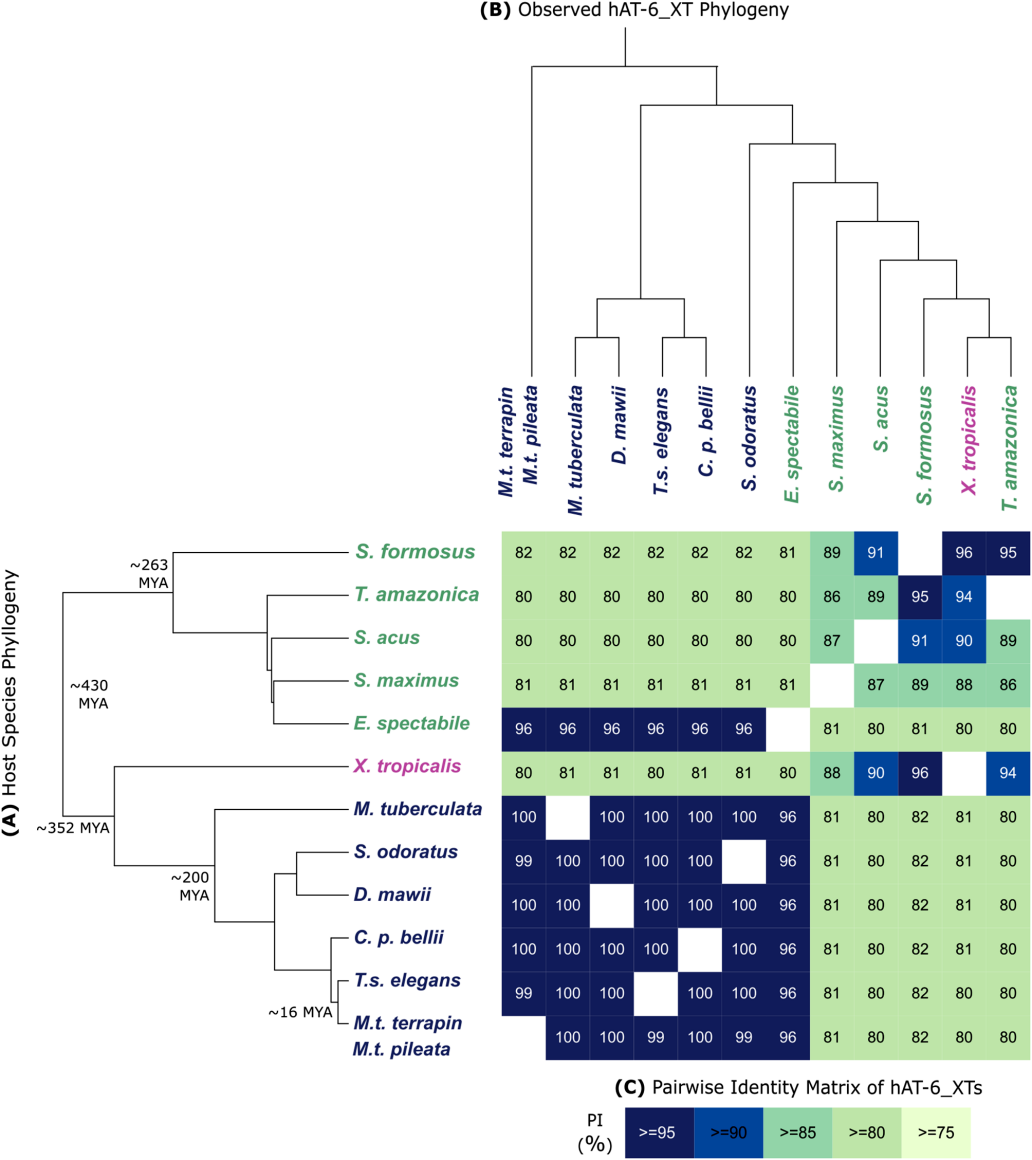
Sequence homology and phylogeny of hAT-6_XT relative to host species phylogeny. (**A**) Host species phylogeny (topology only) of hAT-6_XT host species. The time of divergence between some host species is shown in MYA. (**B**) Observed hAT-6_XT topology constructed using IQTree (1000 bootstraps) based on MAFFT protein alignments trimmed using Clipkit and plotted in iTOL. (**C**) BLASTN pairwise identity matrix of hAT-6_XT representative sequence alignments. Darker shading indicates a higher pairwise identity between two hAT-6_XTs. Turtle species are in blue, frogs are in pink, and fishes are in green

We show that hAT-6_XT TEs are distinct from other hAT and DNA TEs (Fig. 1). In addition, the high sequence similarity of these TEs from distant species, absence in closely related species, and discordant topologies of the species and hAT-6_XT phylogenies (Fig. 2; Additional file 2: Figure S1) make a compelling case for the horizontal transfer of this DNA TE. The first detected transfer of hAT-6_XT was between *Xenopus tropicalis* (Western clawed frog) and *M.t. terrapin* as it satisfied the previous criteria for HTT. A further 12 hAT-6_XTs were found across turtles, a frog, and fish. In addition, given the high sequence identity of hAT-6_XT between pairs of species (Fig. 2), hAT-6_XT may have repeatedly transferred between aquatic animals over time. While we could not determine the direction of transfer, we speculate that transfer is mediated by a parasitic donor.

### The structure of hAT-6_XT transposases indicates activity

To support our case for HTT of hAT-6_XT, we determined the predicted structure and the presence of functional domains required for mobility in the corresponding encoded transposases. The conservation of the TIRs/TSDs in full-length hAT-6_XT transposons is expected and required for HTT. In contrast, the absence of those features in fragmented hAT-6_XT transposons indicates degradation in the genome. We find conservation of such features in most examined hAT-6_XTs (Additional file 1; Table S2). In addition, the functional annotation of hAT-6_XT transposases shows the presence of the essential DDD/E catalytic triad required for transposition and for HTT (Additional file 2: Figure S2-3) [4]. These findings suggest that the hAT-6_XTs have the necessary sequence features and motifs to be active now and thus increase the likelihood that they were recently horizontally transferred.

### hAT-6_XT expansion and divergence

As all hAT-6_XT from turtle genomes are remarkably similar, we selected two closely related species, *M.t. terrapin* and *T.s. elegans*, for downstream investigation. To understand the evolution of each hAT-6_XT transfer in the host species, we investigated the coverage of each element. The coverage and divergence plots of hAT-6_XT in turtles (Additonal file 2: Figure S4) show very low divergence which may be a product of the slow genome evolution [18] of turtles rather than recent HTT events [18]. When combined with patchy phylogenetic distribution in turtles/other species, and how unexpectedly similar hAT-6_XTs are across all species, the evidence points towards HTT in turtles. In comparison to the other species examined in this study, turtles also contained more full-length copies of hAT-6_XT. We were able to rule out horizontal transfer of hAT-6_XT into the most recent common ancestor, prior to the divergence of *Trachemys scripta elegans* (red-eared slider) and *M.t. terrapin* ∽ 16 MYA, and its subsequent preservation in the genomes of ​*T.s. elegans* ​and *M.t. terrapin*​. To determine if HTT of hAT-6_XT occurred independently into turtle species we used a presence/absence analysis to determine if insertions in each species were present or absent from homologous genomic regions (Additional file 2: Figure S5-S10). This analysis showed independent HTT for *M.t. terrapin*, ​*T.s. elegans, C.p. bellii*, *D. mawii*, and *S. odoratus*, but not for *M.t. pilaeta* (subspecies of *M.t. terrapin*). We did not observe full-length hAT-6_XT in the *C.p. bellii* genome assembly, and this could be the result of degradation of hAT-6_XT following HTT, or incompleteness of the assembly (Additional file 2: Figure S28). *M.t. terrapin* and *M.t. pileata* share eight hAT-6_XT insertions indicating HTT into the common ancestor of *M.t. terrapin* with a subsequent sub-species expansion of hAT-6_XT in *M.t. pileata* (Additional file 2: Figure S9).

In *E. spectabile*, where hAT-6_XT divergence is much greater, we also observed a highly amplified region of ∽ 800 bp from hAT-6_XT (Additional file 2: Figure S4). Upon further investigation, we found that this region is a hAT-6_XT derived non-autonomous DNA transposon (Additional file 2: Figure S11). By looking at the Kimura-based divergence of both the hAT-6_XT and hAT-6_XT derived non-autonomous DNA transposon from *E. spectabile* (hAT-6N1_XT_ESp), we see that copy number increase of hAT-6N1_XT_ESp occurred at the same time as copy number increase of hAT-6_XT (Additional file 2: Figure S12). We also observed two additional instances of hAT-6_XT copy number increase in *E. spectabile*, however, these two peaks were also observed in all instances where hAT-6_XT was present, which could indicate reactivation of hAT-6_XT rather than recent HTT (Additional file 2: Figure S13-S25). However, taken together with other evidence of HTT, we hypothesise that there may have been repeated HTT into *E. spectabile*, as opposed to a scenario where the more diverged copies identified in the alignments are degraded versions of a similar hAT.

In FigureS26 (Additional File 2), we show the frequency of hAT-6_XT insertion and divergence relative to other vertically inherited DNA TEs and retrotransposons in turtles. In both *M.t. terrapin* (Fig. 6.1) and *T.s. elegans* (Fig. 6.2), where there has been independent HTT, hAT-6_XT is present in low quantities compared to other elements but shows less than 1% divergence, further supporting the case for HTT. When comparing hAT-6_XT to hAT-3_MTT/TSE, a DNA TE shared by both *M.t. terrapin* and *T.s. elegans*, we see that the hAT-6_XT Kimura divergence and abundance are more similar than expected between the two turtles, despite the divergence between the two species estimated to be 14.5–15.6 MYA [22]. In addition, hAT-3s from both turtles are nearly identical in sequence but show a higher Kimura divergence over time which is consistent with an ancestral repeat. We also show that DNA TEs and retrotransposable elements have similar patterns of copy number increase and divergence, likely indicating the presence of population bottlenecks leading to fixation of insertions [23]. It is important to note that other vertically inherited TEs show peaks consistent with reactivation over time in the turtle genomes, which is not observed with hAT-6_XT even though hAT-6_XTs in turtles appear to have two peaks (marked with arrows) (Additional file 2: Figure S26). When investigating the sequences corresponding to the second peak between 34 and 52% divergence for hAT-6_XT, manual curation revealed that the alignments showed no resemblance to hAT-6_XT, but did resemble other hATs. We observe a similar pattern in hAT-like elements in snakes suggesting they are remnants of other degraded and ancient hATs (Additional file 2; Figure S27).

### Aquatic environments and parasite-host relationships may facilitate HTT

The high percentage identity and low divergence of hAT-6_XT between species are likely the result of independent transfers from an unknown donor(s). The species in this study are largely found in semi-aquatic or aquatic environments but are mostly geographically distant. This indicates possible donor(s) that is/are likely ubiquitously aquatic and/or parasitic in nature. This finding also supports the frequent and recent transfer of transposons in aquatic environments [18, 20, 24]. Horizontal transfer is known to be facilitated by parasite-host relationships with previous findings showing HTT of a retrotransposon from parasitic nematodes [25, 26].

The presence of parasites in aquatic ecosystems involves complex life cycles and may contribute to HTT. The larval stages of certain parasites go through freshwater fish as intermediate hosts and reach turtles as the final hosts, therefore a similar mechanism may happen for frogs and tadpoles [27]. Protozoans and some Metazoans, particularly leeches, exhibit generalist feeding behaviour and can transmit pathogens like trypanosomes [28, 29]. Host specificity of parasites is variable, with some parasites reaching dead-end hosts due to dietary or environmental factors [29, 30]. Parasites in aquatic systems adeptly utilise paratenic hosts, where development is paused until the intermediate host is consumed, thus advancing the parasites through the food chain [29–31]. In addition, there is a geographical overlap between the turtles *M.t. terrapin*, *T.s. elegans*, *C.p. belli*, and *S. odoratus* and *E. spectabile* (Orangethroat darter) suggesting hAT-6_XT HTT into these turtles may have occurred from *E. spectabile* into turtles through a shared parasitic vector, such as darter fish parasites, as all five species have overlapping geographical distributions in North America [32]. However, we cannot rule out the possibility of HTT between turtle species. Finally, as there is no geographical overlap between *X. tropicalis* and the other fishes or turtles, more genomic data from geographically/ecologically overlapping species are required to determine possible donors (Additional file 1; Table S3). As a whole, our study documenting the horizontal transfer of hAT-6_XT DNA transposons among turtles, fishes, and a frog sheds light on the interplay of genetic elements across diverse aquatic species, and provides insights into their genome evolution.

## Conclusions

Overall our study expands our knowledge of HTT in aquatic species and especially the evolution of HTT repeats in the slow-evolving genomes of turtles. We have documented new, recent horizontal transfer events between/into turtles, ray-finned fishes, and a frog, showing that HTT may be more common than expected in turtles. Our findings support the notion that HTT is a common occurrence in ray-finned fishes and suggest that aquatic environments may facilitate a large number of HTT events. The direction of HTT of hAT-6_XT into *M.t. terrapin*, *T.s. elegans*, *C.p. belli*, *S. odoratus* turtles is possibly from *E. spectabile* as the species share habitat and overlap geographically, but we cannot rule out transfer from an unknown donor into all five species. While the donors for HTT are unknown, our results and others from the literature suggest the existence of a cryptic aquatic network of horizontal transfer that is widely distributed given the geographic distances between the HTT recipients.

## Methods

### Identification and classification of horizontally transferred DNA transposon candidates

We performed an *ab initio* repeat annotation for the genome of ​*M.t. terrapin* using the Comprehensive *ab initio* Repeat Pipeline (CARP) [33]. We identified one DNA transposon in *M.t. terrapin* which was originally curated in *X. tropicalis* (Western clawed frog) as hAT-6_XT (Additional file 1; Table S2). We thus renamed the DNA TE in *M.t. terrapin* as hAT-6_XT_MTT. To find more similar sequences to hAT-6_XT_MTT, we performed an extensive local alignment using hAT-6_XT_MTT as a query against the following taxonomic groups: crocodilians, birds, frogs and toads, snakes, turtles, and fish using sensitive BLASTN 2.7.1 + parameters [34] (​word-size: 7, match/mismatch score: 4, -5​) against RefSeq Representative Genomes [35] (Additional file 1; Table S1). A cutoff of 75% identity and 90% coverage to hAT-6_XT_MTT was used to find full-length transposon sequences (​-blastn -e-value 1e-10​). We did global alignments of each sequence back to the query species using MAFFT v7.450 using NCBI coordinates to detect TIRs/TSDs [35, 36]. We classified sequences as full-length transposons or fragments based on the presence of TIRs and TSDs. Representative sequences were selected based on the previous characteristics when multiple copies were found in the respective genomes. Through this process, 13 sequences similar to hAT-6_XT_MTT were found. Open reading frames (ORFs) were identified using GENSCAN and a pairwise identity matrix (PIM) for all 13 sequences was made using BLASTN [37].

### Construction of repeat phylogenies

We aligned the above 13 horizontally transferred hAT-6_XT repeats and representative hAT sequences from RepBase using MAFFT v7.450 [35, 36, 38] (​FFT-NS-1 model​). To select conserved regions for phylogenetic analyses we processed the multiple alignments using Clipkit [39], allowing small final blocks, gap positions within the final blocks and relaxed flanking positions (smart-gap). Two independent tree-building tools were used: IQTree 2 (JTT + G4; 1000 bootstraps​) and Fasttree 2.1 (​JTT + CAT model​) [40, 41]. All tree files were visualized and edited using iTOL [42].

### Construction of species phylogeny

We used TimeTree to construct a species tree of amphibians, bony fishes, and reptiles (https://timetree.org/). The tree was visualised on the Interactive Tree of Life (iTOL) [42, 43]. In the case where a species of interest was not available on TimeTree, we substituted a species from the same clade as a proxy.

### Protein structural analysis of hAT-6_XTs

We determined the structural features of three of the hAT-6_XT DNA transposons we discovered with a protein sequence exceeding 600 amino acids - hAT-6_XT_TSE (*T.s. elegans*), hAT-6_XT_SFo (*S. formosus*), and hAT-6_XT_SAc (*S. acus*) - using AlphaFold [40]. AlphaFold output was visualized using PYMOL (The PyMOL Molecular Graphics System, Version 2.0 Schrödinger, LLC). DDD/E and RW amino acid residues in transposase sequences were identified using M-COFFEE with alignment to the Hermes transposase (Additional file 2; Figure S3) [44].

### Divergence and genome coverage of horizontally transferred DNA transposons

We identified orthologous full-length hAT-6_XT sequences from the genomes we searched (see above) using reciprocal BLAST searches [20, 45]. A custom database was made using the relevant turtle, fishes, and frog genomes as stated above to find the reciprocal best hit using hAT-6_XT_MTT as a nucleotide query from the genome of interest. TE-Aid (https://github.com/clemgoub/TE-Aid) was used to align a sample of full-length hAT-6_XT DNA transposon sequences to each other from the genome it was curated from to visualise the frequency of the complete sequence and fragments.

### Kimura distances

We calculated the Kimura 2-parameter distance for all hAT-6_XT TEs using RepeatMasker version 4.1.5 and the script calcDivergenceFromAlign.pl to obtain the relative age distribution of the TEs in the genome (http://www.repeatmasker.org/RMDownload.html). We also calculated the Kimura 2-parameter distance for other repeats in the Testudine genomes (*T.s. elegans* and *M.t. terrapin*) to determine the age of hAT-6_XT compared to other repeats. We excluded short simple repeat alignments from the analysis.

### Presence/absence test

To determine whether HTT of hAT-6_XT in all the turtle species resulted from a single ancestral event or independent transfers, we conducted a presence/absence test. Using BLASTN 2.7.1 + parameters [34] (blastn-short), all hAT-6_XT intervals were retrieved from the turtle genomes using the representative hAT-6_XT from that genome as a query. The blast output was cleaned to remove hits < 100 bp and converted to bed format with blast2bed.sh (https://github.com/nterhoeven/blast2bed). A genome index file was generated and the cleaned intervals were extended by 1500 bp on each side using BEDTools and SAMtools (slopBed, faidx) [46, 47]. Fasta formatted sequences were extracted for the extended bed intervals using BEDTools (getfasta). The extended hAT-6_XT sequences from *M.t. terrapin* were aligned to each turtle with Gepard and displayed as a dot plot [47, 48].

### Electronic supplementary material

Below is the link to the electronic supplementary material.

Supplementary Material 1

Supplementary Material 2

## Data Availability

The dataset(s) supporting the conclusions of this article are included within the article (and its additional. file(s)).
