# Supplementary material for: Multiple horizontal transfer events of a DNA transposon into turtles, fishes, and a frog
Source: Mob DNA. 2024 Apr 11;15:7. doi: 10.1186/s13100-024-00318-9 (PMC11008031; doi:10.1186/s13100-024-00318-9)
Supplement: Supplementary file 1 — Supplementary Material 1 [file 13100_2024_318_MOESM1_ESM.pdf]

**Table S1**			
**Species screened for hAT-6_XT**			
**Turtles**	**Species name**	**Assembly**	**Homology greater than 80% to hAT-6 and presence of TSDs and TIRs (Y/N)**
	Malaclemys terrapin terrapin	terp_v2_2	Y
	Malaclemys terrapin pileata	rMalTer1.hap1	Y
	Emys orbicularis	rEmyOrb1.hap1	N
	Trachemys scripta elegans	CAS_Tse_1.0	Y
	Gopherus evgoodei	rGopEvg1_v1.p	N
	Mauremys reevesii	ASM1616193v1	N
	Gopherus flavomarginatus	rGopFla2.mat.asm	N
	Dermochelys coriacea	rDerCor1.pri.v4	N
	Terrapene carolina triunguis	T_m_triunguis-2.0	N
	Pelodiscus sinensis	PelSin_1.0	N
	Mauremys mutica	ASM2049712v1	N
	Chelonoidis abingdonii	ASM359739v1	N
	Caretta caretta	GSC_CCare_1.0	N
	Chrysemys picta	Chrysemys_picta_BioNano-3.0.4	N
	Chelonia mydas	rCheMyd1.pri.v2	N
	Carettochelys insculpta	ASM3395843v1	N
	Chelydra serpentina	ASM1885937v1	N
	Chrysemis picta belli	Chrysemys_picta_BioNano-3.0.4	N
	Dermatemys mawii	Dermatemys_mawii-1.0	Y
	Mesoclemmys tuberculata	Mesoclemmys_tuberculata	Y
	Sternotherus odoratus	rSteOdo2_p1.0	Y
	Chrysemis picta	Chrysemys_picta_BioNano-3.0.4	N
**Birds**	**Species name**	**Assembly**	**Homology greater than 80% to hAT-6 and presence of TSDs and TIRs (Y/N)**
	Chlamydotis macqueenii	ASM69519v1	N
	Balearica regulorum gibbericeps	ASM70989v1	N
	Corvus hawaiiensis	bCorHaw1.pri.cur	N
	Tympanuchus pallidicinctus	pur_lepc_1.0	N
	Neopelma chrysocephalum	ASM398488v2	N
	Corvus moneduloides	bCorMon1.pri	N
	Nipponia nippon	ASM70822v1	N
	Melopsittacus undulatus	bMelUnd1.mat.Z	N
	Dryobates pubescens	bDryPub1.pri	N
	Egretta garzetta	ASM68718v1	N
	Falco naumanni	bFalNau1.pat	N
	Aythya fuligula	bAytFul2.pri	N
	Aquila chrysaetos chrysaetos	bAquChr1.4	N
	Lagopus leucura	USGS_WTPT01	N
	Fulmarus glacialis	ASM69083v1	N
	Apteryx australis mantelli	AptMant0	N
	Hirundo rustica	bHirRus1.pri.v2	N
	Chiroxiphia lanceolata	Chiroxiphia lanceolata	N
**Frogs**	**Species name**	**Assembly**	**Homology greater than 80% to hAT-6 and presence of TSDs and TIRs (Y/N)**
	Xenopus tropicalis	UCB_Xtro_10.0	Y
	Bombina bombina	aBomBom1.pri	N
	Nanorana parkeri	ASM93562v1	N
	Bufo bufo	aBufBuf1.1	N
	Spea bombifrons	aSpeBom1.2.pri	N
	Rana temporaria	aRanTem1.1	N
	Bufo gargarizans	ASM1485885v1	N
	Xenopus laevis	Xenopus_laevis_v10.1	N
**Fishes**	**Species name**	**Assembly**	**Homology greater than 80% to hAT-6 and presence of TSDs and TIRs (Y/N)**
	Etheostoma spectabile	UIUC_Espe_1.0	Y
	Thalassophryne amazonica	fThaAma1.1	Y
	Scophthalmus maximus	ASM2237912v1	Y
	Syngnathus acus	fSynAcu1.2	Y
	Scleropages formosus	fSclFor1.1	Y
	Epinephelus lanceolatus	ASM528154v1	N
	Silurus meridionalis	ASM1480568v1	N
	Hippocampus zosterae	ASM2543408v3	N
	Nothobranchius furzeri	UI_Nfuz_MZM_1.0	N
	Tachysurus fulvidraco	HZAU_PFXX_2.0	N
	Melanotaenia boesemani	fMelBoe1.pri	N
	Clarias gariepinus	CGAR_prim_01v2	N
	Carassius gibelio	carGib1.2-hapl.c	N
	Maylandia zebra	M_zebra_UMD2a	N

Insects	Species name	Assembly	Homology greater than 80% to hAT-6 and presence of TSDs and TIRs (Y/N)				
	Acromyrmex echinatior	Aech_3.9	N				
	Drosophila gunungcola	Dgunungcola_SK_2	N				
	Leguminivora glycinivorella	LegGlyc_1.1	N				
	Bombus bifarius	Bbif_JDL3187	N				
	Diaphorina citri	Diaci psyllid	N				
	Cryptotermes secundus	Csec_1.0	N				
	Melitaea cinxia	ilMelCinx1.1	N				
	Drosophila santomea	Prin_Dsan_1.1	N				
	Nilaparvata lugens	ASM1435652v1	N				
	Zootermopsis nevadensis	ZooNev1.0	N				
	Myzus persicae	MPER_G0061.0	N				
	Frankliniella occidentalis	Focc_3.1	N				
	Drosophila subpulchrella	RU_Dsub_v1.1	N				
	Solenopsis invicta	Solenopsis invicta	N				
	Bactrocera oleae	MU_Boleae_v2	N				
**Snakes & Lizards**	**Species name**	**Assembly**	**Homology greater than 80% to hAT-6 and presence of TSDs and TIRs (Y/N)**				
	Podarcis raffonei	rPodRaf1.pri	N				
	Sceloporus undulatus	Sceloporus undulatus	N				
	Pseudonaja textilis	EBS10Xv2-PRI	N				
	Pogona vitticeps	pvi1.1	N				
	Thamnophis sirtalis	Thamnophis_sirtalis-6.0	N				
	Notechis scutatus	TS10Xv2-PRI	N				
	Python bivittatus	Python_molurus_bivittatus-5.0.2	N				
	Gekko japonicus	Gekko_japonicus_V1.1	N				
	Eublepharis macularius	MPM_Emac_v1.0	N				
	Anolis carolinensis	AnoCar2.0	N				
	Pantherophis guttatus	UNIGE_PanGut_3.0	N				
	Thamnophis elegans	rThaEle1.pri	N				
	Varanus komodoensis	ASM479886v1	N				
	Crotalus tigris	ASM1654583v1	N				
	Podarcis muralis	PodMur_1.0	N				
	Zootoca vivipara	UG_Zviv_1	N				
**Mammals**	**Species name**	**Assembly**	**Homology greater than 80% to hAT-6 and presence of TSDs and TIRs (Y/N)**				
	Pipistrellus kuhlii	mPipKuh1	N				
	Mesocricetus auratus	BCM_Maur_2.0	N				
	Peromyscus leucopus	UCI_PerLeu_2.1	N				
	Cricetulus griseus	CriGri_1.0	N				
	Dipodomys ordii	Dord_2.0	N				
	Suncus etruscus	mSunEtr1.pri.cur	N				
	Microtus fortis	M_Fortis_MF-2015_v1.1	N				
	Mus caroli	CAROLI_EIJ_v1.1	N				
	Dipodomys spectabilis	ASM1905484v1	N				
	Phodopus roborovskii	PHOROB	N				
	Rattus rattus	Rrattus_CSIRO_v1	N				
	Heterocephalus glaber	HetGla_female_1.0	N				
	Nannospalax galili	S.galili_v1.0	N				
	Lipotes vexillifer	Lipotes_vexillifer_v1	N				

Table S2

Representative bucleotide sequences of horizontally transferred hAT-6_XT

>hAT-6_XT

CAGGGGTGTCAAACTCAATTGCACAGGGGGCCAAAATCCAAAACACACTTTAGGTCGCGGGCCGAACAGGATAAACATTTATTGAACACACTAAAATTCAGTTGTCTTTGCTCTTATGT
CATTTTATCAGAGCTGGACGCCTGGCATCTTTTTTTGGCAACAAGTTTGTTTATGTTTGGTGTGAGGTTCTGTGTTGTGGAGATTCTCAGGATGGACTGCAGGTGCTCATCAGTGAGAT
GACTCCTGTGTGCTGTTTTGTTAGTCTTCATCACTGAGAAGAGCTTCTCACACAGATATGTGCTACCAAACATGCACAAGGTTCGAGCCGCATGTAGACGGAGCTGGGGCATTTCTGC
GGGAATGGAGTGAATAAACTGTGCGGGCCCTGCAGTGTCGTACTTTGCCTTCAGTGTACCATTACACTGCAGCTCAATCAGCTCCATCTGAATCTGCACAGGTGCAGTTTCCACATCG
ACGGCAAATGGGTTGCGAAGCAACTCAAAATTCTTTTTTTGTTCTTCAAAGTCACCAAAGCGCCGTGCGAACTCAGTGCGCAGTGCGCTCAGTTTATCAGCAAAGTGCGTATTTGGGA
ACACCGTTGTGCCGACTTGGTTCAACATTACTTGGCAACAGGGAAAGTGGGGCAAGTTGCACTGGTGCATTTGTGTCTCCCATAAAAGCAGCTTCACTTGAAATGCCTTCACTGCGTC
ATACATGTCAGTGATCATGCGGTCACATCCCTGGAGCTGGAGGTTTAAGGTGTTGAGATGAGCTGTTATGTCAGTTGTAATGCCAACTCGCATTTCCACTTTTTATCCCTAAAATTGGT
GGAGTCTTTTCCTTTACTGTCCATCAACTGACAGATTTCGTTGCTGAGCTCAAAAACTCTGTTGAGAACTTTTCCCCGACTTAGCCAATGCACCTCTGTATGATAATGAATGTCAGCAAA
CTCTGAATCTATCTCCCGCATAAAGGACTGAAATTGCCGGTGTTTTAAACCTTTAGCTCGGAGTTTACAGTTTGTGTTAAAGTGCTCATTACATTGTCCATCTTCAGGACTTTGCCACAT
AGTGCTTCCTGGTGTATGATGCAGTGATATGCCGTTAACTCACCAGTACAGTTCTCCTCCTGCATCTTTACTCGCATCCTTCCCACTAGTCCACTTTTTTCACTACATATAGATGGTGCT
CCATCAGTTGTAAGTGCAATAAATTTGTCCCAGGGCAGTTTCATGTCAGTTATACTTTGACATACATTTTCAAAAATGTCTTTTCCACTCGTTGTCCCGTGCATCGATTTAATGTCCATTA
TTTCCTCTGTAATGCGCAAATTGGAGTCCACTCCACGGATTAAGATGGCCAGCTGTGCAGTATCAGTCATGTCAGTACTTTCATCCACAGCAAGAGAGTATGCAATAAAGTCTTTGCTT
CTTTCACTCAACTGTGTTCTTAAATCAGTGGCCATCTCACAAACTCGATCAAAAATCGAATTTCTACTCAGGCTTACATTTGCCAGCATCTGCTTTTTGTCTGGACATAAGACGTCAAAC
ACCTTGATCATGCAGCTCTTCAGAAATTCTCCCTTGGTAAATGGCCTGGCTGATTTGGCAATCTCCTCTGCCACGATAAAACTTGCTTTCACAGCAGCTTCACTTTCTGATTTTGCACG
GGTAAAAATCGTTTGCTGAAGTGTCAGATTCTTCTTTAACTCTTCTACTTTCTGTATCTTCTGCTCTGCATTCAGGTCTTTCAGGTTATCCTGATGTTTTGTCTCATAGTGCCGTCTTAAA
TTAAATTCTTTAATTACAGCCACATTAGCTCCACAAATGAGACACAGGGGTTTACCGGCAATGTCAGTAAACATATACTCAGCCTCCCATCGGTTTTTAAAGGCTCTGTTTTTAGAATCA
ACTTTTCTCTTCGGCATCGTGAGGGCTAGCTTCGCAATAACTTGCAGCATCATAAGCTAGACTTGATTAATGCGGGAAGTGTTCGGCAAGGCAGCTGAAGCGCTGCATTATGGGACCT
GTAGTTTATTGTGTTACCAGTGCTTCATATCGCCGGGCCATTAATAACAATAATAAATAAAATGATCTTGCAGGCCGGATATAATTACACACCGGGCCGGATGTGGCCTGCAGGCCTT
GAGTTTGACACATATG

>hAT-6_XT_MTT

CTCTAGGGCAGGGGTGTCAAACTCAATTGCACAGGGGGCCAAAACTCAAAACTCGCGGGCCGAACAGTATAACCATTTATTTAACAGACTAAATATTTATGTTTTTAACCATTAATAT
GAACCAAAATACAGGATATCATTCCAAAATAAATACATTTCAACTTAAAATATTTTGCTCTTCATAAAAAAAATATCCTGTCAATACAATACATTCAAAATCTGTACATGCTGGAATATTAAA
AAATCTGAAAATATAAATAAAAAATTGAATTTAAAGCAAAAGTATAATCATAACAAATCATAACATTCCATTTTCTTGTCCTATTTAGTCAGAGCCTGATACTTGGAGTCTTTTCTTTGCAA
CAAGTTCGTTTATGTTTGGGGTTAGGTTCTGTGTTGTGAAGATTCTCAGGATCGATTGCAGGTGTTCATCAGTGAGGCGACTCCTGTGTGACGTTTTGTTAATTTTCATCACAGAGAAC
AGCTGCTCGCACAGATATGTGCTGCCAAACATGGACAGGATTCGAGCCGCATGTTGGCGGAGCTGTTGCATCGCTTCAGGAATGAAGCGAGTGAACTGTGCTGGCCCCGCAGTGTC
GTACTTTGCCTTCAGTGTCCCGTTACATTGCAGTTCTATCAGCTCCATCTGCATTTCTACAGGTGCGGTTTCCACATCGACTGCAAATGGGTTGCGAAGCAGCTCGAAGTTACTTTTCT
GTGCCTCAAAGTCACTGAAGCGCCGTGCGAACTCAGTGCGCAGCGCGCTGAGTTTTTCAGCAAAGGTGGCATTTGGGAAAACTGTGGCACTTTCTTGGTTCCGTATTACTTGGCAAC
AGGGAAAGTGAGACAAGTTGCATTGGTGCATGTGTGTCTCCCATAAGCGCAGCTTCACTTGAAATGCCTTCACTGCATCATGCATATCGGTTATTATGTGCTCCCGTCCCTGGAGTTG
AAGGTTTAAAGCGCTAAGATGCGACGTTATGTCAGCCAGGAACACCAACTCACATTTCCACTTTTCATCCCGCAGAACTGTGCAGTCTTTCCCCTTACTGTCCATGAACTGGCAGATTT
CCTCTCGCAGCTCAAAGTGTCTTTTGAGAACTTTTCCCCGACTTAGCCACCGGACCTCCGTATGATATGGCATATCGCCAAACTCGCTATCTATTTCCCGCAGAAAAGACTGGAATTG
GCGGTGATTTAAACGTGGCTCTGATAAAGTTGACGGTTTGTGTTACAGTGTTCATCACATGATCCATTTTTAGGACTTTAGCACTCAGCGATTCCTGGTGTATGATGCAGTGATACACT
GTCAACTCACCGGCACAGTTCTCCTCCCGCATCTTTGAGCGCATTCTTCCCACCAGTCCATTTTTTTCACCACACATAGCAGGTGCGCCATCTGTTGTAAGTCCAATGAGTTTTTCCCA
CGGCAGTTTCATGTCGGTTACACTTTGAAATACATTTCCAAAGATGTCTTCTCCTTTCGTTGTCCCGTGCATCGATTTAATGTCCAGTATTTCCTGTGTTACGCACAAATTGGAATCCAC
ACCACGGATAAATATCGCCAGCTGTGCAGTGTCAGTCGCGTCAGTAGTTTCATCCACGGCAAGGGAGTATGCAACAAAATCTTTTGCTCTTTCAATCAACTGTGTTTTCAAATCAGTCG
CCATCTCACAAACCCGATTAGCAACAGTGTTTCTGCTGAGGCTTACATTTGCAAACGCTCGCGTTTTATCTGGACAAAGGACGTCGCACACTTTCATCATACAATTCTTTACGAATTCC
CCCTCGGTAAACGGCCGTCCTGATTTGGCGATCTCTTCGGCCACAATGAAACTTGCTTTCACAGCAGCTTCACTTTGTGATTTGCTTTGGTGAAAAACGTCTGCTGGGATGTCAAATTC
TTCTTCAACTCCTCTACCTTTTGTAGCTTCTGTCCTGCGCTCAGGTTTTTGAATTTGTTCTCATGTTTCGTCTCGTAGTGCCGTCTTAGGTTATACTCCTTCATTACAGCGATATTACTCC
CGCAAAGGAGACACACTGGTTTACCTGCAATTTCAGTAAACATATACTCATTCTCCCACCGGCTTTGAAAGCCTCGGTTTTCAGAATCAATTTTTCTCTTGGCCATTGTTGGGGTCTAG
CTTTGATTTGAGATTAGCGTTGTATACATCAACAGACCGCGAACTTGAACCGCGGCTTGCCGTTGGCGGCAATTGCACTGCATCATGGGATTTGTAGTGTGTGTTATTGGGGCGTCAT
ATCACAGGGCCATTAAAAACAGATATATAAAACGATTTCGCGGGCCGGATATAATTGTATGGCGGGCCGGACGTGGCCCGCGGGCCTTGAGTTTGACACATGTGCTCTAGGG

>hAT-6_XT_TSE

ATCTAGCACAGGGGTGTCAAACTCAATTGCACAGGGGGCCAAAACTCAAAACTCGCGGGCCGAACAGTATAACCATTTATTTAACAGACTAAATATTTATGTTTTTAACCATTAATATG
AACCAAAATACAGGATATCATTCCAAAATAAATACATTTCAACTTAAAATATTTTGCTCTTCATAAAAAAAATATCCTGTCAATACAATACATTCAAAATCTGTACATGCTGGAATATTAAAA
AATCTGAAAATATAAATAAAAAATTGAATTTAAAGCAAAAGTATAATCATAACAAATCATAACATTCCATTTTCTTGTCCTATTTAGTCAGAGCCTGATACTTGGAGTCTTTTCTTTGCAAC
AAGTTCGTTTATGTTTGGGGTTAGGTTCTGTGTTGTGAAGATTCTCAGGATCGATTGCAGGTGTTCATCAGTGAGGCGACTCCTGTGTGACGTTTTGTTAATTTTCATCACAGAGAACA
GCTGCTCGCACAGATATGTGCTGCCAAACATGGACAGGATTCGAGCCGCATGTTGGCGGAGCTGCGGCATCGCTTCAGGAATGAAGCGAGTGAACTGTGCTGGCCCCGCAGTGTC
GTACTTTGCCTTCAGTGTCCCGTTACATTGCAGTTCTATCAGCTCCATCTGCATTTCTACAGGTGCGGTTTCCACATCGACTGCAAATGGGTTGCGAAGCAGCTCGAAGTTACTTTTCT
GTGCCTCAAAGTCACTGAAGCGCCGTGCGAACTCAGTGCGCAGCGCGCTGAGTTTTTCAGCAAAGGTGGCATTTGGGAAAACTGTGGCACTTTCTTGGTTCCGTATTACTTGGCAAC
AGGGAAAGTGAGACAGGTTGCATTGGTGCATTTGTGTCTCCCATAAGCGCAGCTTCACTTGAAATGCCTTCACTGCATCATGCATATCGGTTATTATGTGCTCCCGTCCCTGGAGTTG
AAGGTTTAAAGCGCTAAGATGCGACGTTATGTCAGCCAGGAACGCCAACTCACATTTCCACTTTTCATCCCGCAGAACTGTGCAGTCTTTCCCCTTACTGTCCATGAACTGGCAGATTT
CCTCTCGCAGCTCAAAGTGTCTTTTGAGAACTTTTCCCCGACTTAGCCACCGGACCTCCGTATGATATGGCATATCGCCAAACTCGCTATCTATTTCCCGCAGAAAAGACTGGAATTG
GCGGTGATTTAAACCGTGGGCTCTGATAAAGTTGACGGTTTGTGTTACAGTGTTCATCACATGATCCATTTTTAGGACTTTAGCACTCAGCGATTCCTGGTGTATGATGCAGTGATACA
CTGTCAACTCACCGGCACAGTTCTCCTCCCGCATCTTTGAGCGCATCCTTCCCACCAGTCCATTTTTTTCACCACACATAGCAGGTGCGCCATCTGTTGTAAGTCCAACGAGTTTTTCC
CACGGCAGTTTCATGTCGGTTACACTTTGAAATACATTTCCAAAGATGTCTTCTCCTTTCGTTGTCCCGTGCATCGATTTAATGTCCAGTATTTCCTCTGTTACGCACAAATTGGAATCC
ACACCACGGATAAATATCGCCAGCTGTGCAGTGTCAGTCGCGTCAGTAGTTTCATCCACGGCAAGGGAGTATGCAACAAAATCTTTTGCTCTTTCAATCAACTGTGTTTTCAAATCAGT
CGCCATCTCACAAACCCGATTAGCAACAGTGTTTCTGCTGAGGCTTACATTTGCAAACGCTCGCGTTTTATCTGGACAAAGGACGTCGCACACTTTCATCATACAATTCTTTACGAATT
CCCCCTCGGTAAACGGCCGTCCTGATTTGGCGATCTCTTCGGCCACAATGAAACTTGCTTTCACAGCAGCTTCACTTTGTGATTTTGCTTTGGTGAAAAACGTCTGCTGGGATGTCAA
ATTCTTCTTCAACTCCTCTACCTTTTGTAGCTTCTGTCCTGCGCTCAGGTTTTTGAATTTGTTCTCATGTTTCGTCTCGTAGTGCCGTCTTAGGTTATACTCCTTCATTACAGCGATATTA
CTCCCGCAAAGGAGACACACTGGTTTACCTGCAATTTCAGTAAACATATACTCATTCTCCCACCGGCTTTGAAAGCCTCGGTTTTCAGAATCAATTTTTCTCTTGGCCATTGTTGGGGT
CTAGCTTTGATTTGATATTAGCGTTGTATACATCAACAGACCGCGAACTTGAACCGCGGCTTGCCGTTGGCGGCAATTGCACTGCATCATGGGATTTGTAGTGTGTGTTATTGGGGCG
TCATATCACAGGGCCATTAAAAACAGATATATAAAACGATTTCGCGGGCCGGATATAATTGTATGGCGGGCCGGATGTGGCCCGCGGGCCTTGAGTTTGACACATGTGATCTAGCA

>hAT-6_XT

CTCTAAACCATATGTGTCAAACTCAAGGCCTGCAGGCCACATCCGGCCCGGTGTGTAATTATATCCGGCCTGCAAGATCATTTTATTTATTATTGTTATTAATGGCCCGGCGATATGAA
GCACTGGTAACACAATAAACTACAGGTCCCATAATGCAGCGCTTCAGCTGCCTTGCCGAACACTTCCCGCATTAATCAAGTCTAGCTTATGATGCTGCAAGTTATTGCGAAGCTAGCC
CTCACGATGCCGAAGAGAAAAGTTGATTCTAAAAACAGAGCCTTTAAAAACCGATGGGAGGCTGAGTATATGTTTACTGACATTGCCGGTAAACCCCTGTGTCTCATTTGTGGAGCTA
ATGTGGCTGTAATTAAAGAATTTAATTTAAGACGGCACTATGAGACAAAACATCAGGATAACCTGAAAGACCTGAATGCAGAGCAGAAGATACAGAAAGTAGAAGAGTTAAAGAAGAAT
CTGACACTTCAGCAAACGATTTTTACCCGTGCAAAATCAGAAAGTGAAGCTGCTGTGAAAGCAAGTTTTATCGTGGCAGAGGAGATTGCCAAATCAGCCAGGCCATTTACCAAGGGAG
AATTTCTGAAGAGCTGCATGATCAAGGTGTTTGACGTCTTATGTCCAGACAAAAAGCAGATGCTGGCAAATGTAAGCCTGAGTAGAAATTCGATTTTTTGATCGAGTTTGTGAGATGGC
CACTGATTTAAGAACACAGTTGAGTGAAAGAAGCAAAGACTTTATTGCATACTCTCTTGCTGTGGATGAAAGTACTGACATGACTGATACTGCACAGCTGGCCATCTTAATCCGTGGAG
TGGACTCCAATTTGCGCATTACAGAGGAAATAATGGACATTAAATCGATGCACGGGACAACGAGTGGAAAAGACATTTTTGAAAATGTATGTCAAAGTATAACTGACATGAAACTGCCC
TGGGACAAATTTATTGCACTTACAACTGATGGAGCACCATCTATATGTAGTGAAAAAAGTGGACTAGTGGGAAGGATGCGAGTAAAGATGCAGGAGGAGAACTGTACTGGTGAGTTAA
CGGCATATCACTGCATCATACACCAGGAAGCACTATGTGGCAAAGTCCTGAAGATGGACAATGTAATGAGCACTTTAACACAAACTGTAAACTCCGAGCTAAAGGTTTAAAACACCGG
CAATTTCAGTCCTTTATGCGGGAGATAGATTCAGAGTTTGCTGACATTCATTATCATACAGAGGTGCATTGGCTAAGTCGGGGAAAAGTTCTCAACAGAGTTTTTGAGCTCAGCAACGA
AATCTGTCAGTTGATGGACAGTAAAGGAAAAGACTCCACCAATTTTAGGGATAAAAAGTGGAAATGCGAGTTGGCATTACAACTGACATAACAGCTCATCTCAACACCTTAAACCTCCA
GCTCCAGGGATGTGACCGCATGATCACTGACATGTATGACGCAGTGAAGGCATTTCAAGTGAAGCTGCTTTTATGGGAGACACAAATGTACCAGTACAACTTGCCCCACTTTCCCTGT
TGCCAAGTAATGTTGAACCAAGTCGGCACAACGGTGTTCCCAAATATGCACTTTGCTGATAAACTGAGCGCACTGCGCACGGCGCTTTGGCGACTTTGAAGAACAAAAAAAGAATTTT
GAGTTGCTTCGCAACCCATTTGCCGTCGATGTGGAAACTGCACCTGTGCAGATTCAGATGGAGCTAATTGAGCTGCAGTGTAATGGTACACTGAAGGCAAAGTACGACACTGCAGGG
CCTGCACAGTTTATTCACTCCATTCCCGCAGAAATGCCCCAGCTCCGTCTACATGCAGCTCGAACCTTGTGCATGTTTGGTAGCACATATCTGTGTGAGAAGCTCCTCTCAGTGATGA
AGACTAACAAAACAGCACACAGGAGACATCTCACTGATGAGCACCTGCAGTCCATCCTGAGAATCTCCACACAGAACCTCACACCAAACATAAACAACTTGTTCGTTTGCCAGGCGTC
CAGCTCTGATAAAATGACATAAGAGCAAAGACAACTGAATTTTAGTGTGTTCAATAAATGTTTATCCTGTTCGGCCCGCGACCTAAAGTGTGTTTTGGATTTTGGCCCCTGTGCAATTG
ACACCCTTGCTCTAAAC

>hAT-6_XT_ESp (Contains Mariner-4_Ilyo-like insertion)

GTCTAGAACAGGGGTGTCAAACTCAATTGCACAGGGGGCCAAAACTCAAAACACACTTAATGTCGAGGGCCGAACAGTATTAACATTTATTTAACAGACTAAATATTGGTGTTTTTTAA
CGATTAATATGAATCAAAATACAGGATATCATTCCAAAATAAATAAATTCAACTTAAAATATTTTGCTCTTCATAAAACAATATCCTGTCAATGCAATACATTCAAAATCTGTACATGCTGG
AATATAACAAATCTGAAAATGTAAATGAAAAATGTAATTTAAAGTAAAAGTTAAATCATAACAAAACATAACATTCAATTTTCTTGTCCTATTTAGTCAGAGCCTGAGACTTGGAGTCTTTT
CTTTGCAACAAGTTGGTTTATGTTTGGGGTCAGGTTCTGTGTTGTGGAGATTCTCAGGATGGATTGCAGGTGTTCATCAGTGAGGCGGCTCCTGTGTGACGTTTTGTTAATTTTCATCA
CAGAGAACAGCTGCTCGCACACATACAGTGGTGCTCAAAAGTTTACATACACATGCTTAAGTTGACTAAAAAGAGGAATAAAAAATCATGTTTTGGAAATTTATTTTAATACCTAAATTAA
AAAATGAGGTAAAATCCAACCTTTAAGGACACCAATTTTCTTTGTGAATGAATAACGTATTGTAAATAAATAAATGTTCTTATTTAAAATACAGGGGTCATAAGTATACATACCCCTATGTT
AAATTCCCATAGAGGCAGGCAGATTTTTATTATTAAAGGCCAGTTATTTCCTGGATTCAGGATATTATGCATCCTGATAAAGTTCCCTTGGCCTTTAGAATTAAAATAGCCCCACATCCT
CACATACTCTTCACCATGCTTAGAGATAGGCATGGGATACTTTCTATAAAATCATCTCTCAATGCAAATCAAACCAGGTATTAGTCTAACTGAAATAAAACCATGCCTATCTCTAAGCAT
GGTGAAGAGTATGTGAGGATGTGGGGCTATTTTAATTCTAAAGGCCAAGGGAACTTTATCAGGATGCATAATATCCTGAATCCAGGAAATAACTGGCCTTTAATAATAAAAATCTGCCT
GCCTCTATGGGAATTTAACATAGGGGTATGTATACTTATGACCCCTGTATTTTAAATAAGAACATTTATTTATTTACAATACGTTATTCATTCACAAAGAAAATTGGTGTCCTTAAAGGTT
GGATTTTACCTCATTTTTTAATTTAGGTATTAAAATAAATTTCCAAAACATGATTTTTTATTCCTCTTTTTAGTCAACTTAAGCATGTGTATGTAAACTTTTGAGCACCACTGTATGTGCTGC
CAAACATGGACAGGATTCGAGCCGCATGTTGGCGGAGCTGCGGCATCGCTTCAGGAAGGAAGCGGGTGAACTGTGCTGGCCCCTCAGTGTCGTACTTTGCCTTCAGTGTCCCGTTA
CACTGCAGTTCTATCAGCTCCATCTGCATTTCTACTGGTGCGGTTTCCACGTCGACTGCAAATGGGTTGCGAAGCAGCTCGAAATTACTTTTCTGTGCCTCAAAGTCACTGAAGCGCC
GGGCGAACTCAGTGCGCAGCGCGCTGAGTTTTTCAGCAAAGGTGGCATTTGGGAAAACTGTGTCACTTTCTTGGTTCAGTATTACTTGGCAACAGGGAAAGTGAGACAAGTTGCATT
GGTGCATTTGGGTCTCCCATAAGCGCAGCTTCACTTGAAATGCCTTCACTGCATCATGCATATCAGTTATTATGTGCTCCCGTCCCTGGAGTTGAAGGTTTAAAGCGCTAAGATGCGA
CGTAATGTCAGCCAGGAACGCCAACTCACATTTCCACTTTTCATCCCGCAAAACTGTGCAGTCTTTCCCCTTACTGTCCATGAACTGGCAGATTTCCTCTCGCAGCTCAAAGTGTCTTT
TGAGAACTTTTCCCCGACTCAGCCACCGGACCTCCGTATGATACGGCATATCGCCAAACTCGCAATCTATTTCCCGCAGAAAAGACTGGAATTGGCGGTGATTTAAACCGTGGGCTCT
GATAAAGTTGACGGTTTGTGTTACAGGTTTCATCACATGATCCATTTTTAGGACTTTAGCACTCAGCGATTCCTGGTGTATGATGCAGTGATATACTGTCAACTCACCGGCACAGTTCT
CCTCCCGCATCTTTGAGCGCATCCTTCCCACCAGTCCATTTTTTTTCACCACACATCGCAGGTGCGCCATCTGTTGTAAGTCCAACAAGTTTTTCCCATGGCAGTCTCATGTCGGTTAC
ACTTTGAAATACATTTCCAAAGATGTCTTCTCCTTTCGTTGTCCCGTGCATCGATTTAATGTCCAGTATTTCCTCTGTTACGCACAAATTGGAATCCACTCCACGGATGAATATCGCCAG
CTGTGCAGTGTCAGTCGAGTCAGTAGTTTCATCCACGGCCAGGGAGTATGCAACAAAATCCTTTGCTCTTTCAATCAACTGTGTTTTCAAATCAGTCGCCATCTCACAAACCCGATTAG
CAACAGTGTTTCTGCTGAGGCTTACATTTGCGAATGCTCGCGTTTTATCTGGACAAACGACGTCGCACACTTTCATCATACAATTCTTCACGAATTCCCCCTCGGTAAACGGCCGTCCT
GATTTGGCGATCTCTTCGGCCACAATGAAACTTGCTTTCACAGCAGCTTCACTTTGTGATTTCGCTTTGGTGAAAAACGTCTGCTGGGATGTCAAATTCTTCTTCAACTCCTCTACCTTT
TGTAGCTTCTGTCCTGCGCTCAGGTTTTTTAACTTGTCCTCATGTTTTGTCTCGTAGTGCCGTCTTAGGTTATACTCCTTCATTACAGCGACGTTACTCCCGCAAAGGAGACACACTAAT
TTACCTGCAATTTCAGTAAACATATACTCATTCTCCCACCGGCTTTGAAAGATTCGGTTTTCAGAATACATTTTTCTCTTGGCCATTGTTGTGGTCTAGCTTTGATTTGATATTAGCGTTG
TAGACCGCGAACTTGAACCGCGGCTTGCCGTTGGCGGCAATTGCACTGCATCATGGGATTTGTAGTTTGGGGCGTCATATCACAGGGCCATTAACAACAGATATATACAATGATCAC
GCGGGCCGGATATAATTGTATGGCGGGCCGGATGTGGCCCGCAGGCCTTGAGTTTGACACATGTGGTCTAGAA

>hAT-6_XT_SFo

AATTAAATCAGGGGTGTCAAACTCAATTGCACAGGGGGCCAAAATCCAAAGCACACTTTAGGTCGCGGGCCGAACAGGATAACCATTTATTGAACACACTAAAACTAAACTTTTAAAAC
TTTAAAACTTAACTTTTTTGAACATAAAAATGAATAAAAACAGACAGGAATATTATTCCGGAATAAATCAACTCAAACCTTAAATAACTTATAATATTTTGCTCTCCATAAAAATATATGCTG
TCTAAATTATACAAATTCAAAACATGAACATGCTGCATAACAAAACCTGGAAATATAAATAAAATTTGTGCTTTTCAGCAATAACAAATCAAATCATTCGGTTGTCTTTGCTCTTATGTCAT
TTTATCAGAGCTGGACGCCTGGCATCTTTTTTTGGCAACAAGTTCGTTTATGTTTGGTGTGAGGTTCTGTGTTGTGGAGATTCTCAGGATGGACTGCAGGTGCTCATCAGTGAGACGA
CTCCCGTGTGCTGTTTTGTTAGTCTTCATCACTGAGAAGAGCTTCTCACACAGATATGTGCTACCAAACATGCACAAGGTTCGAGCCGCATGTAGACGGAGCTGGGGCATTTCTGCGG
GAATGGAGTGAATAAACTGTGCGGGCCCTGCAGTGTCGTACTTTGCCTTCAGTGTGCCATTACACTGCAGCTCAATCAGCTCCATCTGAATCTGCACAGGTGCAGTTTCCACATCGAC
GGCAAATGGGTTGCGAAGAAACTCAAAATTCTTTTTTTGTTCTTCAAAGTCACCAAAGCGCCGTGCGAACTCAGTGCGCGGTGCGCTCAGTTTATCAGCAAAGTGCGTATTTGGGAAC
ACCGTTGTGCCGACTTGGTTCAACATTACTTGGCAACAGGGAAAGTGGGGCAAGTTGCACTGGCGCATTTGTGTCTCCCATAAAAGCAGCTTCACTTGAAATGCCTTCACTGCGTCAT
ACATGTCAGTGATCATGCGGTCACGTCCCTGGAGCTGGAGGTTTAAGGCGTTGAGATGAGCTGTTATGTCAGCCAGAAATGCCAACTCGCATTTCCACTTTTCATCCCTAAAATTGGT
GGAGTCTTTTCCTTTACTGTCTATGAACTGACAGATTTCGTTGCTGAGCTCAAAAACTCTGTCGAGAACTTTTCCCCGACTTAGCCAACGCACCTCTGTATGATAAGGAATGTCAGCAA
ACTCTGAATCTATCTCCCGCATAAAGGACTGAAATTGCCGGTGATTTAAACCTTTAGCTCGGATAAAGTTTACGGTTTGTGTTAAAGTGCTCATTACATTGTCCATCTTCAGGACTTTGC
CACATAGTGCTTCCTGGTGTATGATGCAGTGATATGCCGTTAACTCACCAGTACAGTTCTCCTCCTGCATCTTTACTCGCATCCTTCCCACTAGTCCACTTTTTTCACTACACATAGATG
GTGCTCCATCAGTTGTAAGTGCAATAAGTTTGTCCCAGGGCAGTTTCATGTCGGTTATACTTTGACATACATTTTCAAAAATGTCTTTTCCAGTCGTTGTCCCGTGCATCGATTTAATGT
CCATTATTTCCTCTGTGACGCGCAAATTGGAGTCCACTCCACGGATGAAGATGGCCAGCTGTGCAGTATCAGTCATGTCAGTACTTTCATCCACAGCAAGAGAGTATGCAATAAAGTC
TTTGCTTCTTTCACTCAACTGTGTTCTTAAATCAGTGGCCATCTCACGAACTCGATCAGCAATCGTATTTCTACTCAGGCTTACATTTGCCAGCATCTGCTTTTTGTCTGGACATAAGAC
GTCAAACACCTTGATCATGCAGCTCTTCAGAAATTCTCCCTCGGTAAATGGCCTGGCTGATTTGGCGACCTCCTCTGCCACGATAAAACTTGCTTTCACAGCAGCTTCACTTTGTGATT
TTGCACGGGTAAAAAACATCTGCTGAAGTGTCAGATTCTTCTTTAACTCTTCTGCTTTCTGTATCTTCTGCTCTGCATTCAGGTCTTTCAGGTTATCCTGATGTTTTGTCTCATAGTGCCG
TCTTAGATCAAATTCTTTAATTACAGCCACATTAGCTCCACAAATGAGACACACGGGTTTACCGGCAATGTCAGTAAACATATACTCAGCCTCCCATCGGTTTTTAAAGGCTCTGTTTTC
AGAATCAACTTTTCTCTTCGGCATCGTGAGGGCTAGCTTCGCAATAACTTGCAGCATCATAAGCTAGACTTGATTAACGCGGGAAATGTTCGGCAAGGCAGCTGAAGCGCTGCATTAT
GGGATCTGTAGTTTATTGTGTTACCAGCGCTTCATATCGCCGGGCCATTAATAACAATAATATATAAAATGATCTCGCGGGCCGGATATAATTACACGCCGGGCCGGATGTGGCCCGC
GGGCCTTGAGTTTGACACATATGAATTAAAT

>hAT-6_XT_TAm

TTCTAGGGCAGGGGTGTCAAACTCAATTGCACAGGGGGCCAAAATCCAAAACACACTTTCGGTCACGGGCCGAACAGGATAAACATTTATTGAATACATTAAACTTTAAAACTTTTAAA
ACTTAACTTTTTTGAACATGAATATGAATAAAAACAGACAGGACTATTATTCTGGAATAAATCAACTTAAACCTTAAATAACTTCTAATATTTTGCTCTCCATAAAAATATATCCTGTCTAAA
TTATACAAGTTAGAAAAAAGTAAACGTTCAAATGTCTTTACTCTTACATTATTTTATATAAAAAATAAACTTAAATTTAAACTTACTAAAATATCCCAAAAGATTTTGCTCTCCATAAAAATAT
ATCCTGTCAAAATTATACAAATTCAAAATATGAACATGCTGCATAACAAAACCTGGAAATATAAATAAAATTTCAGCAATAACAAATCAAATCATTCAGTTTTTTTTGCTCTTATGTCGTTT
TATCAAAGCTGGATGCCTGGCATCTTTTTTTGGCAACAAGTTCCTTTATGTTTGGTGTGAGTTTCTGTGTTGTGGAGATTCTCAGGATGGACTGCAGGTGCTCATCAGTGAGATGACTC
CTGTGTGCTGTTTTGTTAGTCTTCATCACTGAGAAGAGCTTCTCACACAGATATGTGCTACCAAACATGCACAAAGTTCGAGCCACATGTAGACGGAGCTGGGGCATTTCTGCAGGAA
TGGCGTGAATAAACTGTGGGGGCCCTGCAGTGTCATATTTTGCCTTCAGTGTGCCATTACACTGCAGCTCAATCAGCTTCATCTGAATCTGCACAGGTGCAGTTTCCACATCGACGGC
AAATGGGTTGCGAAGCAACTCCAAATTCTTTTTTTGTTCTTCAAAGTCACCAAAGCGCCGTGCAAACTCAGTGCGCAGTGCACTCAGTTTATCAGCAAAGTGCATATTTGGGAACACCG
TTGTGCCCGCTTGGTTCAACATTACTTGGCAACAGGGAGAGTGGGACATGTTGCACTGGTGCATTTGTGACTCCCATAAAAGCAGCTTCACTTGAAATGCCTTTACTGCGTCATACAT
GTCAGTGATCATGCGGTCACGTCCCTGGAGCTGGAGGTTTAAGGCGTTGAGATGAGCTGTTATGTCAGCCAGAAATGCCAACTTGCATTTCCACTTTTCATCCCTAAAATTGGTGGAG
TCTTTTCCTTTACTGTCCATGAACTGACAGATTTTGTTGCTGAGCTCAAAAACTCTGCTGAGAACTTTTCCCCAACTTAGCCAACGCACCTCTGTATGATAAGGAATGTCAGCAAACTCT
GAATCTATCTCCTGCAAATTGCCGGTGATTTAAACCTTTAGCTCGGATAAAGTTTAGGGTTTGTGTTACAGTGCTCATTACATGGTCCATCTTCAGGACTTTGCCACATAGTGCTTCCTG
GCGTATAATGCAGTGATATGCAGTTAACTCACCAGTACAGTTCTCCTGCATCTTTACTCGCATCCTTCCCACTAGTCCTCTTTTTTCACTACACATAGATGGCGCTCCATCAGTAGTAAG
TGCAATACGTTTGTCCCAGGGCAGTTTCATGTCGGTTATACTTTGACATACATTTTCAAAAATGTCTTTTCCAGTCGTTGTCCCGTGCATCAATTTAATGTCCATTATTTCCTCTGTAACA
CGCAAATTGGAGTCCACTCCATGGATGAAGATGGCCAGCTGTGCAGTATCAGTCATGTCAGTACTTTCATCCACAGCAAGAGAGTATGCAATAAAGTCTTTGCTTCTTTCACTCAACTG
TGTTCTTAAATCAGTGGCCATCTCACAAACTCGATCAGCAATCATCTTTCTACTCAGGCTTACATTTGCCAGAATCTGCTTTTTGTCTGGACATAAGACGTCAAACACCTTGATCATGCA
GCTCTTCAGAAATTCTCCCTCTGTAAATGGCCTGGCTGATTTGGCGATCTCCTCTGCCGCGATAAAACTTGATTTCACAGCAGCTTCACTTTGTGATTTTGCACGGGTAAAAAATGTCT
GCTGAAGTGTCAAATTCTTCTTTAAGTCTTCTGCTTTCTGTATCTTCTGCTCTGCATTCAGGTCTTTCAGGTTATCCTGATGTTTTGTCTCACAGTGCCGTCTTAGATTAAATTCTTTAATT
ACAGCCACATTATCTCCACAAATGAGACACACGGGTTTACCGGCAATGTCAGTAAACATATACTCAGCCTCCCATCGACTTTTAAAGGCTCTGTTTTCAGAATCAACTTTTCTCTTCGG
CATCGTGAGGGCTCAATAACTCGGGCGCAATAACTTGCAGCATCATAAGCTAGGCTAAATGAACGTGAAGCATTTGGCAAGGCAGCTGAAGCGCTGCATTATGGGATCTGTAGTTTAT
TGTCTTTATTGCCAGCGCTTCATATTGCCGGGCCATTAATAACAATAATATATAAAATGATCTCGCAAGCTGGATAAAATCACACGCCGGGCCGGATATGGCCCGCATGCCTCGAGTTT
GACACATATGTTCTAGGG

>hAT-6_XT_SAc

GTCTGGAGCAGGAGTGTCAAACTCAATTGGACAGGGGGCCGAAATTCAAAACACACTTCAGGTCGCGGGCCGAACAGAACAAACATTTATTGAACACACTAAAACTAACTTTTAACAT
AAATATAAATACAAACGGACAGGAATGTTATTCTGGAATATATAAACTTAAACTTTAAATAACTTAAAATATCTTGATCTCCATAAAAATATACCCAGTTAAAATTATACAAGTTAGAAAAA
TATCTCCTGTCAAAATTATACAAGATAGAAATATGAACATGCTGCAAAACAAAACAAAACCTGGAAATATAAAAACAATTTGTGCTTTTCAGCAATAACAAATAATGAATCAAAACATTCA
GTTTTCTTTGCTCTTATGCCATTTTATCAGAGCCGGATGATTGGCATCTTTTTTTGGCAACAAGTTGGTTTATGTTTGGTGTGAGGTCCCGTGTTGTGGAGACTCTCAGGATGGACTGA
AGGTGCTCATCAGTGAGACGACTCCTGTGTGCTGTTTTGTTTGTCTTCATCACTGAGAACAGCTTCTCACACAGATATGTGCTACCAAACATGCACAAGGTTCGAGCCACATGTAGAC
GGAGTTGGGACATTGCTGCGGGAATGGAGTGAATAAACTCTGTGGGCCCTGCAGTGTCGTACTTTGACTTTAGCGTCCCATTACACTGCAGCTCAATCAGCTCCATCTGAATCTGAAC
AGGTGCAGATTCCACGTCGACGGCAAATGGGTTGCGAAACAACTCGAAATTCTTCTTTTGTTCCTCAAAATCACCAAAGCGCCGTGCGAACTCCGTGCGCAGTGCGCTCAGTTTAACA
GCAAAGTGCGTATTTGGGAACACCGTTGTGCCGACTTGGTTCAGCATTACTTGGCAACAGGGAAAGTGGGACAAGTTGCACTGAAGCATTTGTGTCTCCCATAAAATCAGCTTCACTT
GAAATGCCTTCACTGCGTCATACATGTCAGTGATCATGCGGTCACGTCCCTGGAGCTGGAGGTTTAAGGCATTGAGATGTGCGGTTATGTCAGCTAGAAACGCCAACTCACATTTCCA
CTTTTCATCCCGTAAAACGGTGGAGTCTTTGCCTTTACTGTCCATGAACTGACAAATTTCGTTGCTGAGCTCAAAAACTCTGTTGAGAACTTTCCCCCGACTTAGCCAACGCACCTCTG
TATGATAAGGAATGTCGGCAAACTCTGAATCTATAGTCCGCATAAAGGACTGAAATTGCCGGTGATTTAAACCTCTACTTCGGATAAAGTTTACGGTTTGCGTTACAGTGCTCATTACAT
GGTCCATCTTCAGGACTTTGCCACACAGTGCTTCCTGGTGTATGATGCAGTGGTATGTTGTTAACTCACCAGTACAGTTCTCCTCCTGCATCTTTTCTCGCATCCTTCCTACTAGTCCA
CTTTTCTCACCGCACATAGCCGGTGCTCCATCAGTTGTAAGACCAATAAGTTTGTCCCAGGGCAGTTTCATGTCGGTTACACTTTGACATACGTTTTCAAAAATGTCCTTTCCGGTCGT
TGTCCCGTGCATCGGTTTAATGTCCAATATTTCCTCAGTAACACGCAAATCGGAGTCCACCCCACGGATGAAGATGGCTAGCTCTGCAGTGTCAGTCATGTCAGTACTTTCATCCATA
GCAAGAGAGTATGCAATAAAGTCTTTGCTTCTTTTACTCAACTGTGTTCTTAAATCAGTGGCCATCTCACAAACTCGATCAGCAACCGTATTTCTACTCAGGCTTACATTTGCCAGCATC
TGCTTTTTGTCTGGACATATGATGTCGCACACCTTGACCATGCAGCTCTTCAGAAATTCTCCCTCTGTGAATGGCCGGCCTGCTTTGGCGATCTCCTCTGCCACGATAAAACTCGCTTT
CACAGCAGCTTCACTTTGTGATTTTGCACGGGTGAAAAACGTCTGCTGGAATGTCAGCTTCTTCTTTAACTCTTCTACCTTCTGTATCTTCTGCTCTGCATTCAGGTCTTTCAGGTTATC
CAGATGTTTTGTTTCATAGTGCCGTCTTAGATTAAACTCTTTCAATACAGCCACATTAGCGCCACAAATGAGACAAACGGGCTTACCGGCAATGTCAGTAAACATATACTCAACCTCCC
ATCGGTTTTTAAAGGCTCTGTTTTCAGAATCAACTTTTCTCTTCGGCATCATGAGGGGCTAGCTTCGCAATAACTTGCAGCAACAAGCACTTGACCTGATTGCCGCGGAAAAACGCTCG
GCAAGGGAGCGAAAGCACTGCATTATGGGATCTGTAGTGTTTTTTCTTTTTTTTTTTTTTTCATATCGTCGGGCCATTAGTAACAATAATATAAAATGATCTCGCGGGCCGGATATAATT
GCATGCCGGGCCGGATGTGGCCCGCGGGCCTTGAGTTTGACACATATGGTTTAGGG

>hAT-6_XT_SMa

TAGAGCAGGGGTGTCAAACTCAATTGCACATGGGGCCAAAACTCAAAGCACACCTCAGGTCGCGGGCCGAACAGGATAAACATTTATTGAACACCCTTAAACTACACGTTTGAACATG
AATATGAATAAAACAGACAGGAATATTATTTCAGAATAAATAAACTTGCACTTTAAATAAAATAAAATGTATTTTGCTCTCCATAAAAATATATCCTGTCAATAATTATACAAGTTCGAAATA
TGAACATGCTGCAAAAAACCCCTGAAAAAAATAAATAAAAATAGTGCTTTTAAGTAAACGTTAAAATAAATGAAAACATGTTTCTTTGTTCTTATGCCATTTTGTCAGAGCAGGATGTTTG
GCATCTTTTTTTGGCAGTAAGATCGTTTAGGTTTGGTGTGAGTTCCTGTGCTGTGGAGATCCTCAGGATGGACTGCAGGTGTCCGTCAGTGAGACGACTCCTGTGTGCTGTTTTGTTC
ATCTTCATCACTGAGAACAGCTTCTCACACAGATATGTGCTACCAAACATGCACAAGGTTTGAGCAGCATGCAGACGGAGCTGGGGCATTGTTTCAGGAATTGAGCGAATAAATTGTG
CGGGCCCTGCAGTGTCGTACTTCGCCTTTAGTGTCCCATTACACTGCAGCTCAATCAGCTCCATCTGAATCTGTACAGGAGCAGTTTCCACGTCGACGGCAAACGGGTTGCGAAAGT
CACCAAAGCGCCGCGCGAACTCAGTGCGTAGTGCGCTCAGTTTGTCAGCAAAGTGCGTATTTGGGAACACCGTTGCGCCGACTTGGTTAAACATTACTTGGCAACAGGGGAAGTGA
GGCAAGTTGGACTGGCGCATTTGTGTCTCCCATAAGAGCAGCTTCACTTTAAATGCCTTCACCGTATCGTACATGTCAGTGATTATGCGGTCACGTCCCTGGAGATGAAGGTTCAAGA
CGTTGAGATGAGCTGTTACGTCAGCCAGAAACGCCAGCTCACATTTCCACTTTTCATCCCGCAAAACTGTGGTGTCTTTTCCTTTACTGTCCATGAACTGACAGATTTCCTTGCTGAGC
TCAAAAACTCTATTGAGAACTTTTCCCCGACTCAGCCAGCGCACCTCTGTATGATAAGGAATGTCGGCAAACTCTGAATCTATTTCCCGCATAAAAGACTGAAATTGCCAGTGATTTAA
ACCTTTAGCTCGGATAAAGTTTACGGTTTGCGTTACAGTGTTCATTACATGCTCCATTTTTAGGACTTTACAACACAGCATTTCCTGGTGTATGATGCAGTGATACGCTGTTAGCTCACC
AGTACAGTTCTCCTCCTGCATCTTTGCTCGCATCCTTCCCACCAGTCCAACTTTTTCACTGCACATCGCCGGTGCTCCGTCTGTTGTAAGTCCAATAAGTTTGTCCCAGGGCAGTTTCA
TGTCAGTTATGCTTTGACATACGTTTTCAAAAATGTCTTTTCCTGTCGTTGTTCCATGCATCGATTTAATGTCCAATATTTCCTCTGTAACACACAAACTGGAGTCCACTTCACGGATGAA
AATGGCCAGCTGTGCAGTGTCCGTCATGTCGGTACTTTCATCAACAGCAAGAGAAAATGCAGTAAAGTCTTTGCTTCTCTCACTCAACTGTGTTTTTAAATCAGTGGCCATCTCACAAA
TCCGATCAGCAACCGTATTTCTACTCAGGCTTACATTTGCAAACATCTGCTTATTTTCTGGACACAAGACATCGCACACCTTCATCATGCAGCTCTTCAGAAACTCTCCCTCGGTAAATG
GACGGGCTGATTTGGCTATCTCCTGTGCCACGATAAAACTTGCTTTCACAGCAGCTTCACTTTGGGATTTCGCTCTGGTGAAAAACGTCTGCTGAAATCTCAGATTCTTCTTTAACTCTT
TTACTCTCTGTATCTTCTCCTCTGCATTCAGGTTTTGCAGCTCCTGATGTTTTGTCTCGTAGTGCCGCCGTATATTAAATTCCTTAATTACAGCCACATTAGCTCCACAAACGAGACACA
CAGGTTTACCAGCAATGTCAGTAAACATGTACTCAGCCTCCCATCGGTTTTGAAATGCTCTGTTTTCAGAATCAACTTTTCTCTTCGCCATTGTGAGGGGCTAGCTTCGCAATAACTTTT
CAACGGGGTTGTAGCAGCCGGTTGTAGCACAAAAGAAGCTGACTTGATTGACGCGGAAAAGTTCCCGGGTATCCGAGTGTCCGTTACTTGGTTACTGAAGCACTGCATTATGGGATC
TGTAGTTTGTGTATTGTCAGCGCTTCATGTCACCGGGCCATAATAATAGATCTATATAAAATTATCTTGCAGGCCGGATATAATTGCACGCCGGGCCGGATGTGGCCCGCGGGCCTTG
AGCTTGACATATATGACGTAGAG

>hAT-6_XT_MTP

CAGGGGTGTCAAACTCAATTGCACAGGGGGCCAAAACTCAAAACTCGCGGGCCGAACAGTATAACCATTTATTTAACAGACTAAATATTTATGTTTTTTAACCATTAATATGAACCAAAA
TGCAGGATATCATTCCAAAATAAATACATTTCAACTTAAAATATTTTGCTCTTCATAAAAAAAATATCCTGTCAATACAATACATTCAAAATCTGTACATGCTGGAATATTAAAAAATCTGAA
AATATAAATAAAAAAATTGAATTTAAAGCAAAAGTATAATCATAACAAATCATAACATTCCATTTTCTTGTCCTATTTAGTCAGAGCCTGATACTTGGAGTCTTTTCTTTGCAACAAGTTCGT
TTATGTTTGGGGTTAGGTTCTGTGTTGTGAAGATTCTCAGGATCGATTGCAGGTGTTCATCAGTGAGGCGACTCCTGTGTGACGTTTTGTTAATTTTCATCACAGAGAACAGCTGCTCG
CACAGATATGTGCTGCCAAACATGGACAGGATTCGAGCCGCATGTTGGCGGAGCTGTTGCATCGCTTCAGGAATGAAGCGAGTGAACTGTGCTGGCCCCGCAGTGTCGTACTTTGC
CTTCAGTGTCCCGTTACATTGCAGTTCTATCAGCTCCATCTGCATTTCTACAGGTGCGGTTTCCACATCGACTGCAAATGGGTTGCGAAGCAGCTCGAAGTTACTTTTCTGTGCCTCAA
AGTCACTGAAGCGCCGTGCGAACTCAGTGCGCAGCGCGCTGAGTTTTTCAGCAAAGGTGGCATTTGGGAAAACTGTGGCACTTTCTTGGTTCCGTATTACTTGGCAACAGGGAAAGT
GAGACAAGTTGCATTGGTGCATGTGTGTCTCCCATAAGCGCAGCTTCACTTGAAATGCCTTCACTGCATCATGCATATCGGTTATTATGTGCTCCCGTCCCTGGAGTTGAAGGTTTAAA
GCGCTAAGATGCGACGTTATGTCAGCCAGGAACACCAACTCACATTTCCACTTTTCATCCCGCAGAACTGTGCAGTCTTTCCCCTTACTGTCCATGAACTGGCAGATTTCCTCTCGCA
GCTCAAAGTGTCTTTTGAGAACTTTTCCCCGACTTAGCCACCGGACCTCCGTATGATATGGCATATCGCCAAACTCGCTATCTATTTCCCGCAGAAAAGACTGGAATTGGCGGTGATTT
AAACCGTGGGCTCTGATAAAGTTGACGGTTTGTGTTACAGTGTTCATCACATGATCCATTTTTAGGACTTTAGCACTCAGCGATTCCTGGTGTATGATGCAGTGATACACTGTCAACTC
ACCGGCACAGTTCTCCTCCCGCATCTTTGAGCGCATTCTTCCCACCAGTCCATTTTTTTCACCACACATAGCAGGTGCGCCATCTGTTGTAAGTCCAACGAGTTTTTCCCACGGCAGTT
TCATGTCGGTTACACTTTGAAATACATTTCCAAAGATGTCTTCTCCTTTCGTTGTCCCGTGCATCGATTTAATGTCCAGTATTTCCTGTGTTACGCACAAATTGGAATCCACACCACGGA
TAAATATCGCCAGCTGTGCAGTGTCAGTCGCGTCAGTAGTTTCATCCACGGCAAGGGAGTATGCAACAAAATCTTTTGCTCTTTCAATCAACTGTGTTTTCAAATCAGTCGCCATCTCA
CAAACCCGATTAGCAACAGTGTTTCTGCTGAGGCTTACATTTGCAAACGCTCGCGTTTTATCTGGACAAAGGACGTCGCACACTTTCATCATACAATTCTTTACGAATTCCCCCTCGGT
AAACGGCCGTCCTGATTTGGCGATCTCTTCGGCCACAATGAAACTTGCTTTCACAGCAGCTTCACTTTGTGATTTTGCTTTGGTGAAAAACGTCTGCTGGGATGTCAAATTCTTCTTCA
ACTCCTCTACCTTTTGTAGCTTCTGTCCTGCGCTCAGGTTTTTGAATTTGTTCTCATGTTTCGTCTCGTAGTGCCGTCTTAGGTTATACTCCTTCATTACAGCGATATTACTCCCGCAAA
GGAGACACACTGGTTTACCTGCAATTTCAGTAAACATATACTCATTCTCCCACCGGCTTTGAAAGCCTCGGTTTTCAGAATCAATTTTTCTCTTGGCCATTGTTGGGGTCTAGCTTTGAT
TTGAGATTAGCGTTGTATACATCAACAGACCGCGAACTTGAACCGCGGCTTGCCGTTGGCGGCAATTGCACTGCATCATGGGATTTGTAGTGTGTGTTATTGGGGCGTCATATCACAG
GGCCATTAAAAACAGATATATAAAACGATTTCGCGGGCCGGATATAATTGTATGGCGGGCCGGACGTGGCCCGCGGGCCTTGAGTTTGACACATGTGCTCTAGGG

>hAT-6_XT_CPB

CAGGGGTGTCAAACTCAATTGCACAGGGGGCCAAAACTCAAAACTCGCGGGCCGAACAGTATAACCATTTATTTAACAGACTAAATATTTATGTTTTTTAACCATTAATATGAACCAAAA
TACAGGATATCATTCCAAAATAAATACATTTCAACTTAAAATATTTTGCTCTTCATAAAAAAAATATCCTGTCAATACAATACATTCAAAATCTGTACATGCTGGAATATTAAAAAATCTGAAA
ATATAAATAAAAAAATTGAATTTAAAGCAAAAGTATAATCATAACAAATCATAACATTCCATTTTCTTGTCCTATTTAGTCAGAGCCTGATACTTGGAGTCTTTTCTTTGCAACAAGTTCGTT
TATGTTTGGGGTTAGGTTCTGTGTTGTGAAGATTCTCAGGATCGATTGCAGGTGTTCATCAGTGAGGCGACTCCTGTGTGACGTTTTGTTAATTTTCATCACAGAGAACAGCTGCTCGC
ACAGATATGTGCTGCCAAACATGGACAGGATTCGAGCCGCATGTTGGCGGAGCTGCGGCATCGCTTCAGGAATGAAGCGAGTGAACTGTGCTGGCCCCGCAGTGTCGTACTTTGCC
TTCAGTGTCCCGTTACATTGCAGTTCTATCAGCTCCATCTGCATTTCTACAGGTGCGGTTTCCACATCGACTGCAAATGGGTTGCGAAGCAGCTCGAAGTTACTTTTCTGTGCCTCAAA
GTCACTGAAGCGCCGTGCGAACTCAGTGCGCAGCGCGCTGAGTTTTTCAGCAAAGGTGGCATTTGGGAAAACTGTGGCACTTTCTTGGTTCCGTATTACTTGGCAACAGGGAAAGTG
AGACAAGTTGCATTGGTGCATTTGTGTCTCCCATAAGCGCAGCTTCACTTGAAATGCCTTCACTGCATCATGCATATCGGTTATTATGTGCTCCCGTCCCTGGAGTTGAAGGTTTAAAG
CGCTAAGATGCGACGTTATGTCAGCCAGGAACGCCAACTCACATTTCCACTTTTCATCCCGCAGAACTGTGCAGTCTTTCCCCTTACTGTCCATGAACTGGCAGATTTCCTCTCGCAG
CTCAAAGTGTCTTTTGAGAACTTTTCCCCGACTTAGCCACCGGACCTCCGTATGATATGGCATATCGCCAAACTCGCTATCTATTTCCCGCAGAAAAGACTGGAATTGGCGGTGATTTA
AACCGTGGGCTCTGATAAAGTTGACGGTTTGTGTTACAGTGTTCATCACATGATCCATTTTTAGGACTTTAGCACTCAGCGATTCCTGGTGTATGATGCAGTGATACACTGTCAACTCA
CCGGCACAGTTCTCCTCCCGCATCTTTGAGCGCATCCTTCCCACCAGTCCATTTTTTTCACCACACATAGCAGGTGCGCCATCTGTTGTAAGTCCAACGAGTTTTTCCCACGGCAGTTT
CATGTCGGTTACACTTTGAAATACATTTCCAAAGATGTCTTCTCCTTTCGTTGTCCCGTGCATCGATTTAATGTCCAGTATTTCCTCTGTTACGCACAAATTGGAATCCACACCACGGAT
AAATATCGCCAGCTGTGCAGTGTCAGTCGCGTCAGTAGTTTCATCCACGGCAAGGGAGTATGCAACAAAATCTTTTGCTCTTTCAATCAACTGTGTTTTCAAATCAGTCGCCATCTCAC
AAACCCGATTAGCAACAGTGTTTCTGCTGAGGCTTACATTTGCAAACGCTCGCGTTTTATCTGGACAAAGGACGTCGCACACTTTCATCATACAATTCTTTACGAATTCCCCCTCGGTA
AACGGCCGTCCTGATTTGGCGATCTCTTCGGCCACAATGAAACTTGCTTTCACAGCAGCTTCACTTTGTGATTTTGCTTTGGTGAAAAACGTCTGCTGGGATGTCAAATTCTTCTTCAA
CTCCTCTACCTTTTGTAGCTTCTGTCCTGCGCTCAGGTTTTTGAATTTGTTCTCATGTTTCGTCTCGTAGTGCCGTCTTAGGTTATACTCCTTCATTACAGCGATATTACTCCCGCAAAG
GAGACACACTGGTTTACCTGCAATTTCAGTAAACATATACTCATTCTCCCACCGGCTTTGAAAGCCTCGGTTTTCAGAATCAATTTTTCTCTTGGCCATTGTTGGGGTCTAGCTTTGATT
TGAGATTAGCGTTGTATACATCAACAGACCGCGAACTTGAACCGCGGCTTGCCGTTGGCGGCAATTGCACTGCATCATGGGATTTGTAGTGTGTGTTATTGGGGCGTCATATCACAG
GGCCATTAAAAACAGATATATAAAACGATTTCGCGGGCCGGATATAATTGTATGGCGGGCCGGATGTGGCCCGCGGGCCTTGAGTTTGACACATGTGCTCTA

>hAT-6_XT_SOd

CAGGGGTGTCAAACTCAATTGCACAGGGGGCCAAAACTCAAAACTCGCGGGCTGAACAGTATAACCATTTATTTAACAGACTAAATATTTATGTTTTTTAACCATTAATATGAACCAAAA
TACAGGATATCATTCCAAAATAAATACATTTCAACTTAAAATATTTTGCTCTTCATAAAAAAATATCCTGTCAATACAATACATTCAAAATCTGTACATGCTGGAATATTAAAATATCTGAAA
ATATAAATAAAAAATTGAATTTAAAGCAAAAGTATAATCATAACAAATCATAACATTCCATTTTCTTGTCCTATTTAGTCAGAGCCTGATACTTGGAGTCTTTTCTTTGCAACAAGTTCGTT
TATGTTTGGGGTTAGGTTCTGTGTTGTGAAGATTCTCAGGATCGATTGCAGGTGTTCATCAGTGAGGCGACTCCTGTGTGACGTTTTGTTAATTTTCATCACAGAGAACAGCTGCTCGC
ACAGATATGTGCTGCCAAACATGGACAGGATTCGAGCCGCATGTTGGCGGAGCTGCGGCATCGCTTCAGGAATGAAGCGAGTGAACTGTGCTGGCCCCGCAGTGTCGTACTTTGCC
TTCAGTGTCCCGTTACATTGCAGTTCTATCAGCTCCATCTGCATTTCTACAGGTGCGGTTTCCACATCGACTGCAAATGGGTTGCGAAGCAGCTCGAAGTTACTTTTCTGTGCCTCAAA
GTCACTGAAGCGCCGTGCGAACTCAGTGCGCAGCGCGCTGAGTTTTTCAGCAAAGGTGGCATTTGGGAAAACTGTGGCACTTTCTTGGTTCCGTATTACTTGGCAACAGGGAAAGTG
AGACAAGTTGCATTGGTGCATTTGTGTCTCCCATAAGCGCAGCTTCACTTGAAATGCCTTCACTGCATCATGCATATCGGTTATTATGTGCTCCCGTCCCTGGAGTTGAAGGTTTAAAG
CGCTAAGATGCGACGTTATGTCAGCCAGGAACGCCAACTCACATTTCCACTTTTCATCCCGCAGAACTGTGCAGTCTTTCCCCTTACTGTCCATGAACTGGCAGATTTCCTCTCGCAG
CTCAAAGTGTCTTTTGAGAACTTTTCCCCGACTTAGCCACCGGACCTCCGTATGATATGGCATATCGCCAAACTCGCTATCTATTTCCCGCAGAAAAGACTGGAATTGGCGGTGATTTA
AACCGTGGGCTCTGATAAAGTTGACGGTTTGTGTTACAGTGTTCATCACATGATCCATTTTTAGGACTTTAGTACTCAGCGATTCCTGGTGTATGATGCAGTGATACACTGTCAACTCA
CCGGCACAGTTCTCCTCCCGCATCTTTGAGCGCATCCTTCCCACCAGTCCATTTTTTTCACCACACATAGCAGGTGCGCCATCTGTTGTAAGTCCAACGAGTTTTTCCCACGGCAGTTT
CATGTCGGTTACACTTTGAAATACATTTCCAAAGATGTCTTCTCCTTTCGTTGTCCCGTGCATCGATTTAATGTCCAGTATTTCCTCTGTTACGCACAAATTGGAATCCACACCACGGAT
AAATATCGCCAGCTGTGCAGTGTCAGTCGCGTCAGTAGTTTCATCCACGGCAAGGGAGTATGCAACAAAATCTTTTGCTCTTTCAATCAACTGTGTTTTCAAATCAGTCGCCATCTCAC
AAACCCGATTAGCAACAGTGTTTCTGCTGAGGCTTACATTTGCAAACGCTCGCGTTTTATCTGGACAAAGGACGTCGCACACTTTCATCATACAATTCTTTACGAATTCCCCCTCGGTA
AACGGCCGTCCTGATTTGGCGATCTCTTCGGCCACAATGAAACTTGCTTTCACAGCAGCTTCACTTTGTGATTTTGCTTTGGTGAAAAACGTCTGCTGGGATGTCAAATTCTTCTTCAA
CTCCTCTACCTTTTGTAGCTTCTGTCCTGCGCTCAGGTTTTTGAATTTGTTCTCATGTTTCGTCTCGTAGTGCCGTCTTAGGTTATACTCCTTCATTACAGCGACATTACTCCCGCAAAG
GAGACACACTGGGTTACCTGCAATTTCAGTAAACATATACTCATTCTCCCACCGGCTTTGAAAGCCTCGGTTTTCAGAATCAATTTTTCTCTTGGCCATTGTTGGGGTCTAGCTTTGATT
TGATATTAGCGTTGTATACATCAACAGACCGCGAACTTGAACCGCGGCTTGCCGTTGGTGGCAATTGCACTGCATCATGGGATTTGTAGTGTGTGTTATTGGGGCGTCATATCACAGG
GCCATTAAAAACAGATATATAAAACGATTTCGCGGGCCGGATATAATTGTATGGCGGGCCGGATGTGGCCCGCGGGCCTTGAGTTTGACACATGTGCTCTAG

>hAT-6_XT_MTu

CAGGGGTGTCAAACTCAATTGCACAGGGGGCCAAAACTCAAAACTCGCGGGCCGAACAGTATAACCATTTATTTAACAGACTAAATATTTATGTTTTTTAACCATTAATATGAACCAAAA
TACAGGATATCATTCCAAAATAAATACATTTCAACTTAAAATATTTTGCTCTTCATAAAAAAATATCCTGTCAATACAATACATTCAAAATCTGTACATGCTGGAATATTAAAAAATCTGAAA
ATATAAATAAAAAATTGAATTTAAAGCAAAAGTATAATCATAACAAATCATAACATTCCATTTTCTTGTCCTATTTAGTCAGAGCCTGATACTTGGAGTCTTTTCTTTGCAACAAGTTCGTT
TATGTTTGGGGTTAGGTTCTGTGTTGTGAAGATTCTCAGGATCGATTGCAGGTGTTCATCAGTGAGGCGACTCCTGTGTGACGTTTTGTTAATTTTCATCACAGAGAACAGCTGCTCGC
ACAGATATGTGCTGCCAAACATGGACAGGATTCGAGCCGCATGTTGGCGGAGCTGCGGCATCGCTTCAGGAATGAAGCGAGTGAACTGTGCTGGCCCCGCAGTGTCGTACTTTGCC
TTCAGTGTCCCGTTACATTGCAGTTCTATCAGCTCCATCTGCATTTCTACAGGTGCGGTTTCCACATCGACTGCAAATGGGTTGCGAAGCAGCTCGAAGTTACTTTTCTGTGCCTCAAA
GTCACTGAAGCGCCGTGCGAACTCAGTGCGCAGCGCGCTGAGTTTTTCAGCAAAGGTGGCATTTGGGAAAACTGTGGCACTTTCTTGGTTCCGTATTACTTGGCAACAGGGAAAGTG
AGACAAGTTGCATTGGTGCATTTGTGTCTCCCATAAGCGCAGCTTCACTTGAAATGCCTTCACTGCATCATGCATATCGGTTATTATGTGCTCCCGTCCCTGGAGTTGAAGGTTTAAAG
CGCTAAGATGCGACGTTATGTCAGCCAGGAACGCCAACTCACATTTCCACTTTTCATCCCGCAGAACTGTGCAGTCTTTCCCCTTACTGTCCATGAACTGGCAGATTTCCTCTCGCAG
CTCAAAGTGTCTTTTGAGAACTTTTCCCCGACTTAGCCACCGGACCTCCGTATGATATGGCATATCGCCAAACTCGCTATCTATTTCCCGCAGAAAAGACTGGAATTGGCGGTGATTTA
AACCGTGGGCTCTGATAAAGTTGACGGTTTGTGTTACAGTGTTCATCACATGATCCATTTTTAGGACTTTAGCACTCAGCGATTCCTGGTGTATGATGCAGTGATACACTGTCAACTCA
CCGGCACAGTTCTCCTCCCGCATCTTTGAGCGCATCCTTCCCACCAGTCCATTTTTTTCACCACACATAGCAGGTGCGCCATCTGTTGTAAGTCCAACGAGTTTTTCCCACGGCAGTTT
CATGTCGGTTACACTTTGAAATACATTTCCAAAGATGTCTTCTCCTTTCGTTGTCCCGTGCATCGATTTAATGTCCAGTATTTCCTCTGTTACGCACAAATTGGAATCCACACCACGGAT
AAATATCGCCAGCTGTGCAGTGTCAGTCGCGTCAGTAGTTTCATCCACGGCAAGGGAGTATGCAACAAAATCTTTTGCTCTTTCAAGCAACTGTGTTTTCAAATCAGTCGCCATCTCAC
AAACCCGATTAGCAACAGTGTTTCTGCTGAGGCTTACATTTGCAAACGCTCGCGTTTTATCTGGACAAAGGACGTCGCACACTTTCATCATACAATTCTTTACGAATTCCCCCTCGGTA
AACGGCCGTCCTGATTTGGCGATCTCTTCGGCCACAATGAAACTTGCTTTCACAGCAGCTTCACTTTGTGATTTTGCTTTGGTGAAAAACGTCTGCTGGGATGTCAAATTCTTCTTCAA
CTCCTCTACCTTTTGTAGCTTCAGTCCTGCGCTCAGGTTTTTGAATTTGTTCTCATGTTTCGTCTCGTAGTGCCGTCTTAGGTTATACTCCTTCATTACAGCGATATTACTCCCGCAAAG
GAGACACACTGGTTTACCTGCAATTTCAGTAAACATATACTCATTCTCCCACCGGCTTTGAAAGCCTCGGTTTTCAGAATCAATTTTTCTCTTGGCCATTGTTGGGGTCTAGCTTTGATT
TGATATTAGCGTTGTATACATCAACAGACCGCGAACTTGAACCGCGGCTTGCCGTTGGCGGCAATTGCACTGCATCATGGGATTTGTAGTGTGTGTTATTGGGGCGTCATATCACAGG
GCCATTAAAAACAGATATATAAAACGATTTCGCGGGCCGGATATAATTGTATGGCGGGCCGGATGTGGCCCGCGGGCCTTGAGTTTGACACATGTG

>hAT-6_XT_DMa

CAGGGGTGTCAAACTCAATTGCACAGGGGGCCAAAACTCAAAACTCGCGGGCCGAACAGTATAACCATTTATTTAACAGACTAAATATTTATGTTTTTTAACCATTAATATGAACCAAAA
TACAGGATATCATTCCAAAATAAATACATTTCAACTTAAAATATTTTGCTCTTCATAAAAAAATATCCTGTCAATACAATACATTCAAAATCTGTACATGCTGGAATATTAAAAAATCTGAAA
ATATAAATAAAAAATTGAATTTAAAGCAAAAGTATAATCATAACAAATCATAACATTCCATTTTCTTGTCCTATTTAGTCAGAGCCTGATACTTGGAGTCTTTTCTTTGCAACAAGTTCGTT
TATGTTTGGGGTTAGGTTCTGTGTTGTGAAGATTCTCAGGATCGATTGCAGGTGTTCATCAGTGAGGCGACTCCTGTGTGACGTTTTGTTAATTTTCATCACAGAGAACAGCTGCTCGC
ACAGATATGTGCTGCCAAACATGGACAGGATTCGAGCCGCATGTTGGCGGAGCTGCGGCATCGCTTCAGGAATGAAGCGAGTGAACTGTGCTGGCCCCGCAGTGTCGTACTTTGCC
TTCAGTGTCCCGTTACATTGCAGTTCTATCAGCTCCATCTGCATTTCTACAGGTGCGGTTTCCACATCGACTGCAAATGGGTTGCGAAGCAGCTCGAAGTTACTTTTCTGTGCCTCAAA
GTCACTGAAGCGCCGTGCGAACTCAGTGCGCAGCGCGCTGAGTTTTTCAGCAAAGGTGGCATTTGGGAAAACTGTGGCACTTTCTTGGTTCCGTATTACTTGGCAACAGGGAAAGTG
AGACAAGTTGCATTGGTGCATTTGTGTCTCCCATAAGCGCAGCTTCACTTGAAATGCCTTCACTGCATCATGCATATCGGTTATTATGTGCTCCCGTCCCTGGAGTTGAAGGTTTAAAG
CGCTAAGATGCGACGTTATGTCAGCCAGGAACGCCAACTCACATTTCCACTTTTCATCCCGCAGAACTGTGCAGTCTTTCCCCTTACTGTCCATGAACTGGCAGATTTCCTCTCGCAG
CTCAAAGTGTCTTTTGAGAACTTTTCCCCGACTTAGCCACCGGACCTCCGTATGATATGGCATATCGCCAAACTCGCTATCTATTTCCCGCAGAAAAGACTGGAATTGGCGGTGATTTA
AACCGTGGGCTCTGATAAAGTTGACGGTTTGTGTTACAGTGTTCATCACATGATCCATTTTTAGGACTTTAGCACTCAGCGATTCCTGGTGTATGATGCAGTGATACACTGTCAACTCA
CCGGCACAGTTCTCCTCCCGCATCTTTGAGCGCATCCTTCCCACCAGTCCATTTTTTTCACCACACATAGCAGGTGCGCCATCTGTTGTAAGTCCAACGAGTTTTTCCCACGGCAGTTT
CATGTCGGTTACACTTTGAAATACATTTCCAAAGATGTCTTCTCCTTTCGTTGTCCCGTGCATCGATTTAATGTCCAGTATTTCCTCTGTTACGCACAAATTGGAATCCACACCACGGAT
AAATATCGCCAGCTGTGCAGTGTCAGTCGCGTCAGTAGTTTCATCCACGGCAAGGGAGTATGCAACAAAATCTTTTGCTCTTTCAAGCAACTGTGTTTTCAAATCAGTCGCCATCTCAC
AAACCCGATTAGCAACAGTGTTTCTGCTGAGGCTTACATTTGCAAACGCTCGCGTTTTATCTGGACAAAGGACGTCGCACACTTTCATCATACAATTCTTTACGAATTCCCCCTCGGTA
AACGGCCGTCCTGATTTGGCGATCTCTTCGGCCACAATGAAACTTGCTTTCACAGCAGCTTCACTTTGTGATTTTGCTTTGGTGAAAAACGTCTGCTGGGATGTCAAATTCTTCTTCAA
CTCCTCTACCTTTTGTAGCTTCAGTCCTGCGCTCAGGTTTTTGAATTTGTTCTCATGTTTCGTCTCGTAGTGCCGTCTTAGGTTATACTCCTTCATTACAGCGATATTACTCCCGCAAAG
GAGACACACTGGTTTACCTGCAATTTCAGTAAACATATACTCATTCTCCCACCGGCTTTGAAAGCCTCGGTTTTCAGAATCAATTTTTCTCTTGGCCATTGTTGGGGTCTAGCTTTGATT
TGATATTAGCGTTGTATACATCAACAGACCGCGAACTTGAACCGCGGCTTGCCGTTGGCGGCAATTGCACTGCATCATGGGATTTGTAGTGTGTGTTATTGGGGCGTCATATCACAGG
GCCATTAAAAACAGATATATAAAACGATTTCGCGGGCCGGATATAATTGTATGGCGGGCCGGATGTGGCCCGCGGGCCTTGAGTTTGACACATGTG

>hAT-6_XT_MTT

MATDLKTQLIERAKDFVAYSLAVDETTDATDTAQLAIFIRGVDSNLCVTQEILDIKSMHGTTKGEDIFGNVFQSVTDMKLPWEKLIGLTTDGAPAMCGEKNGLVGRMRSKMREENCAEPRLNH
RQFQSFLREIDSEFGDMPYHTEVRWLSRGKVLKRHFELREEICQFMDSKGKDCTVLRDEKWKCELVFLADITSHLSALNLQLQGREHIITDMHDAVKAFQVKLRLWETHMHQCNLSHFPCCQ
VIRNQESATVFPNATFAEKLSALRTEFARRFSDFEAQKSNFELLRNPFAVDVETAPVEMQMELIELQCNGTLKAKYDTAGPAQFTRFIPEAMQQLRQHAARILSMFGSTYLCEQLFSVMKINKT
SHRSRLTDEHLQSILRIFTTQNLTPNINELVAKKRLQVSGSD

>hAT-6_XT_MTP

MAKRKIDSENRGFQSRWENEYMFTEIAGKPVCLLCGSNIAVMKEYNLRRHYETKHENKFKNLSAGQKLQKVEELKKNLTSQQTFFTKAKSQSEAAVKASFIVAEEIAKSGRPFTEGEFVKNCM
MKVCDVLCPDKTRAFANVSLSRNTVANRVCEMATDLKTQLIERAKDFVAYSLAVDETTDATDTAQLAIFIRGVDSNLCVTQEILDIKSMHGTTKGEDIFGNVFQSVTDMKLPWEKLVGLTTDGA
PAMCGEKNGLVGRMRSKMREENCAGELTVYHCIIHQESLSAKVLKMDHVMNTVTQTVNFIRAHGLNHRQFQSFLREIDSEFGDMPYHTEVRWLSRGKVLKRHFELREEICQFMDSKGKDCT
VLRDEKWKCELVFLADITSHLSALNLQLQGREHIITDMHDAVKAFQVKLRLWETHMHQCNLSHFPCCQVIRNQESATVFPNATFAEKLSALRTEFARRFSDFEAQKSNFELLRNPFAVDVETA
PVEMQMELIELQCNGTLKAKYDTAGPAQFTRFIPEAMQQLRQHAARILSMFGSTYLCEQLFSVMKINKTSHRSRLTDEHLQSILRIFTTQNLTPNINELVAKKRLQVSGSD

>hAT-6_XT_CPB

MAKRKIDSENRGFQSRWENEYMFTEIAGKPVCLLCGSNIAVMKEYNLRRHYETKHENKFKNLSAGQKLQKVEELKKNLTSQQTFFTKAKSQSEAAVKASFIVAEEIAKSGRPFTEGEFVKNCM
MKVCDVLCPDKTRAFANVSLSRNTVANRVCEMATDLKTQLIERAKDFVAYSLAVDETTDATDTAQLAIFIRGVDSNLCVTEEILDIKSMHGTTKGEDIFGNVFQSVTDMKLPWEKLVGLTTDGA
PAMCGEKNGLVGRMRSKMREENCAGELTVYHCIIHQESLSAKVLKMDHVMNTVTQTVNFIRAHGLNHRQFQSFLREIDSEFGDMPYHTEVRWLSRGKVLKRHFELREEICQFMDSKGKDCT
VLRDEKWKCELAFLADITSHLSALNLQLQGREHIITDMHDAVKAFQVKLRLWETQMHQCNLSHFPCCQVIRNQESATVFPNATFAEKLSALRTEFARRFSDFEAQKSNFELLRNPFAVDVETA
PVEMQMELIELQCNGTLKAKYDTAGPAQFTRFIPEAMPQLRQHAARILSMFGSTYLCEQLFSVMKINKTSHRSRLTDEHLQSILRIFTTQNLTPNINELVAKKRLQVSGSD

>hAT-6_XT_SOd

MAKRKIDSENRGFQSRWENEYMFTEIAGNPVCLLCGSNVAVMKEYNLRRHYETKHENKFKNLSAGQKLQKVEELKKNLTSQQTFFTKAKSQSEAAVKASFIVAEEIAKSGRPFTEGEFVKNC
MMKVCDVLCPDKTRAFANVSLSRNTVANRVCEMATDLKTQLIERAKDFVAYSLAVDETTDATDTAQLAIFIRGVDSNLCVTEEILDIKSMHGTTKGEDIFGNVFQSVTDMKLPWEKLVGLTTDG
APAMCGEKNGLVGRMRSKMREENCAGELTVYHCIIHQESLSTKVLKMDHVMNTVTQTVNFIRAHGLNHRQFQSFLREIDSEFGDMPYHTEVRWLSRGKVLKRHFELREEICQFMDSKGKDC
TVLRDEKWKCELAFLADITSHLSALNLQLQGREHIITDMHDAVKAFQVKLRLWETQMHQCNLSHFPCCQVIRNQESATVFPNATFAEKLSALRTEFARRFSDFEAQKSNFELLRNPFAVDVET
APVEMQMELIELQCNGTLKAKYDTAGPAQFTRFIPEAMPQLRQHAARILSMFGSTYLCEQLFSVMKINKTSHRSRLTDEHLQSILRIFTTQNLTPNINELVAKKRLQVSGSD

>hAT-6_XT_MTu

MAKRKIDSENRGFQSRWENEYMFTEIAGKPVCLLCGSNIAVMKEYNLRRHYETKHENKFKNLSAGLKLQKVEELKKNLTSQQTFFTKAKSQSEAAVKASFIVAEEIAKSGRPFTEGEFVKNCM<br>MKVCDVLCPDKTRAFANVSLSRNTVANRVCEMATDLKTQLLERAKDFVAYSLAVDETTDATDTAQLAIFIRGVDSNLCVTEEILDIKSMHGTTKGEDIFGNVFQSVTDMKLPWEKLVGLTTDGA<br>PAMCGEKNGLVGRMRSKMREENCAGELTVYHCIIHQESLSAKVLKMDHVMNTVTQTVNFIRAHGLNHRQFQSFLREIDSEFGDMPYHTEVRWLSRGKVLKRHFELREEICQFMDSKGKDCT<br>VLRDEKWKCELAFLADITSHLSALNLQLQGREHIITDMHDAVKAFQVKLRLWETQMHQCNLSHFPCCQVIRNQESATVFPNATFAEKLSALRTEFARRFSDFEAQKSNFELLRNPFAVDVETA<br>PVEMQMELIELQCNGTLKAKYDTAGPAQFTRFIPEAMPQLRQHAARILSMFGSTYLCEQLFSVMKINKTSHRSRLTDEHLQSILRIFTTQNLTPNINELVAKKRLQVSGSD
>hAT-6_XT_DMa
MAKRKIDSENRGFQSRWENEYMFTEIAGKPVCLLCGSNIAVMKEYNLRRHYETKHENKFKNLSAGLKLQKVEELKKNLTSQQTFFTKAKSQSEAAVKASFIVAEEIAKSGRPFTEGEFVKNCM<br>MKVCDVLCPDKTRAFANVSLSRNTVANRVCEMATDLKTQLLERAKDFVAYSLAVDETTDATDTAQLAIFIRGVDSNLCVTEEILDIKSMHGTTKGEDIFGNVFQSVTDMKLPWEKLVGLTTDGA<br>PAMCGEKNGLVGRMRSKMREENCAGELTVYHCIIHQESLSAKVLKMDHVMNTVTQTVNFIRAHGLNHRQFQSFLREIDSEFGDMPYHTEVRWLSRGKVLKRHFELREEICQFMDSKGKDCT<br>VLRDEKWKCELAFLADITSHLSALNLQLQGREHIITDMHDAVKAFQVKLRLWETQMHQCNLSHFPCCQVIRNQESATVFPNATFAEKLSALRTEFARRFSDFEAQKSNFELLRNPFAVDVETA<br>PVEMQMELIELQCNGTLKAKYDTAGPAQFTRFIPEAMPQLRQHAARILSMFGSTYLCEQLFSVMKINKTSHRSRLTDEHLQSILRIFTTQNLTPNINELVAKKRLQVSGSD
>hAT-6_XT_TSE
MAKRKIDSENRGFQSRWENEYMFTEIAGKPVCLLCGSNIAVMKEYNLRRHYETKHENKFKNLSAGQKLQKVEELKKNLTSQQTFFTKAKSQSEAAVKASFIVAEEIAKSGRPFTEGEFVKNCM<br>MKVCDVLCPDKTRAFANVSLSRNTVANRVCEMATDLKTQLIERAKDFVAYSLAVDETTDATDTAQLAIFIRGVDSNLCVTEEILDIKSMHGTTKGEDIFGNVFQSVTDMKLPWEKLVGLTTDGA<br>PAMCGEKNGLVGRMRSKMREENCAGELTVYHCIIHQESLSAKVLKMDHVMNTVTQTVNFIRAHGLNHRQFQSFLREIDSEFGDMPYHTEVRWLSRGKVLKRHFELREEICQFMDSKGKDCT<br>VLRDEKWKCELAFLADITSHLSALNLQLQGREHIITDMHDAVKAFQVKLRLWETQMHQCNLSHFPCCQVIRNQESATVFPNATFAEKLSALRTEFARRFSDFEAQKSNFELLRNPFAVDVETA<br>PVEMQMELIELQCNGTLKAKYDTAGPAQFTRFIPEAMPQLRQHAARILSMFGSTYLCEQLFSVMKINKTSHRSRLTDEHLQSILRIFTTQNLTPNINELVAKKRLQVSGSD
>hAT-6_XT
MPKRKVDSKNRAFKNRWEAEYMFTDIAGKPLCLICGANVAVIKEFNLRRHYETKHQDNLKDLNAEQKIQKVEELKKNLTLQQTIFTRAKSESEAAVKASFIVAEEIAKSARPFTKGEFLKSCMIK<br>VFDVLCPDKKQMLANIQMELIELQCNGTLKAKYDTAGPAQFIHSIPAEMPQLRLHAARTLCMFGSTYLCEKLLSVMKTNKTAHRRHLTDEHLQSILRISTQNLTPNINNLFVCQASSSDKMT
>hAT-6_XT_ESp
MAKRKMYSENRIFQSRWENEYMFTEIAGKLVCLLCGSNVAVMKEYNLRRHYETKHEDKLKNLSAGQKLQKVEELKKNLTSQQTFFTKAKSQSEAAVKASFIVAEEIAKSGRPFTEGEFVKNC<br>MMKVCDVVCPDKTRAFANVSLSRNTVANRVCEMATDLKTQLIERAKDFVAYSLAVDETTDSTDTAQLAIFIRGVDSNLCVTEEILDIKSMHGTTKGEDIFGNVFQSVTDMRLPWEKLVGLTTDG<br>APAMCGEKKWTGGKDALKDAGGELCRAHGLNHRQFQSFLREIDCEFGDMPYHTEVRWLSRGKVLKRHFELREEICQFMDSKGKDCTVLRDEKWKCELAFLADITSHLSALNLQLQGREHIIT<br>DMHDAVKAFQVKLRLWETQMHQCNLSHFPCCQVILNQESDTVFPNATFAEKLSALRTEFARRFSDFEAQKSNFELLRNPFAVDVETAPVEMQMELIELQCNGTLKAKYDTEGPAQFTRFLPE<br>AMPQLRQHAARILSMFGSTYSGAQKFTYTCLS
>hAT-6_XT_SFo
MLQVIAKLALTMPKRKVDSENRAFKNRWEAEYMFTDIAGKPVCLICGANVAVIKEFDLRRHYETKHQDNLKDLNAEQKIQKAEELKKNLTLQQMFFTRAKSQSEAAVKASFIVAEEVAKSARPF<br>TEGEFLKSCMIKVFDVLCPDKKQMLANVSLSRNTIADRVREMATDLRTQLSERSKDFIAYSLAVDESTDMTDTAQLAIFIRGVDSNLRVTEEIMDIKSMHGTTTGKDIFENVCQSITDMKLPWDK<br>LIALTTDGAPSMCSEKSGLVGRMRVKMQEENCTGELTAYHCIIHQEALCGKVLKMDNVMSTLTQTVNFIRAKGLNHRQFQSFMREIDSEFADIPYHTEVRWLSRGKVLDRVFELSNEICQFIDS<br>KGKDSTNFRDEKWKCELAFLADITAHLNALNLQLQGRDRMITDMYDAVKAFQVKLLLWETQMRQCNLPHFPCCQVMLNQVGTTVFPNTHFADKLSAPRTEFARRFGDFEEQKKNFEFLRNP<br>FAVDVETAPVQIQMELIELQCNGTLKAKYDTAGPAQFIHSIPAEMPQLRLHAARTLCMFGSTYLCEKLFSVMKTNKTAHGSRLTDEHLQSILRISTTQNLTPNINELVAKKRCQASSSDKMT
>hAT-6_XT_TAm
MPKRKVDSENRAFKSRWEAEYMFTDIAGKPVCLICGDNVAVIKEFNLRRHCETKHQDNLKDLNAEQKIQKAEDLKKNLTLQQTFFTRAKSQSEAAVKSSFIAAEEIAKSARPFTEGEFLKSCMI<br>KVFDVLCPDKKQILANVSLSRKMIADRVCEMATDLRTQLSERSKDFIAYSLAVDESTDMTDTAQLAIFIHGVDSNLRVTEEIMDIKLMHGTTTGKDIFENVCQSITDMKLPWDKRIALTTDGAPSM<br>CSEKRGLVGRMRVKMQENCTEVRWLSWGKVLSRVFELSNKICQFMDSKGKDSTNFRDEKWKCKLAFLADITAHLNALNLQLQGRDRMITDMYDAVKAFQVKLLLWESQMHQCNMSHSPC<br>CQVMLNQAGTTVFPNMHFADKLSALRTEFARRFGDFEEQKKNLELLRNPFAVDVETAPVQIQMKLIELQCNGTLKAKYDTAGPPQFIHAIPAEMPQLRLHVARTLCMFGSTYLCEKLFSVMKT<br>NKTAHRSHLTDEHLQSILRISTTQKLTPNIKELVAKKRCQASSFDKTT
>hAT-6_XT_SAc
MPKRKVDSENRAFKNRWEVEYMFTDIAGKPVCLICGANVAVLKEFNLRRHYETKHLDNLKDLNAEQKIQKVEELKKKLTFQQTFFTRAKSQSEAAVKASFIVAEEIAKAGRPFTEGEFLKSCMV<br>KVCDIICPDKKQMLANVSLSRNTVADRVCEMATDLRTQLSKRSKDFIAYSLAMDESTDMTDTAELAIFIRGVDSDLRVTEEILDIKPMHGTTTGKDIFENVCQSVTDMKLPWDKLIGLTTDGAPA<br>MCGEKSGLVGRMREKMQEENCTGELTTYHCIIHQEALCGKVLKMDHVMSTVTQTVNFIRSRGLNHRQFQSFMRTIDSEFADIPYHTEVRWLSRGKVLNRVFELSNEICQFMDSKGKDSTVLR<br>DEKWKCELAFLADITAHLNALNLQLQGRDRMITDMYDAVKAFQVKLILWETQMLQCNLSHFPCCQVMLNQVGTTVFPNTHFAVKLSALRTEFARRFGDFEEQKKNFELFRNPFAVDVESAPV<br>QIQMELIELQCNGTLKSKYDTAGPTEFIHSIPAAMSQLRLHVARTLCMFGSTYLCEKLFSVMKTNKTAHRSRLTDEHLQSILRVSTTRDLTPNINQLVAKKRCQSSGSDKMA
>hAT-6_XT_SMa
MAKRKVDSENRAFQNRWEAEYMFTDIAGKPVCLVCGANVAVIKEFNIRRHYETKHQELQNLNAEEKIQRVKELKKNLRFQQTFFTRAKSQSEAAVKASFIVAQEIAKSARPFTEGEFLKSCMM<br>KVCDVLCPENKQMFANVSLSRNTVADRICEMATDLKTQLSERSKDFTAFSLAVDESTDMTDTAQLAIFIREVDSSLCVTEEILDIKSMHGTTTGKDIFENVCQSITDMKLPWDKLIGLTTDGAPA<br>MCSEKVGLVGRMRAKMQEENCTGELTAYHCIIHQEMLCCKVLKMEHVMNTVTQTVNFIRAKGLNHWQFQSFMREIDSEFADIPYHTEVRWLSRGKVLNRVFELSKEICQFMDSKGKDTTVL<br>RDEKWKCELAFLADVTAHLNVLNLHLQGRDRIITDMYDTVKAFKVKLLLWETQMRQSNLPHFPCCQVMFNQVGATVFPNTHFADKLSALRTEFARRFGDFRNPFAVDVETAPVQIQMELIELQ<br>CNGTLKAKYDTAGPAQFIRSIPETMPQLRLHAAQTLCMFGSTYLCEKLFSVMKMNKTAHRSRLTDGHLQSILRISTAQELTPNLNDLTAKKRCQTSCSDKMA

**Table S3**	
**Geographical ranges of species with horizontally transferred hAT-6_XT**	
**Species**	**Geographic range**
T. s. elegans	North America
M. t. terrapin	North America
E. spectabile	North America
T. amazonica	Amazon
S. maximus	Northeast Atlantic, Mediterranian Sea
S. acus	British Isles, Mediterranian Sea
S. foromsus	Southeast Asia
X. tropicalis	West Africa
M. t. pilenta	North America
C. p. bellii	North America
D. mawii	Central America
S. odoratus	North America
M. tuberculta	South America
