# Supplementary material for: Multiple horizontal transfer events of a DNA transposon into turtles, fishes, and a frog
Source: Mob DNA. 2024 Apr 11;15:7. doi: 10.1186/s13100-024-00318-9 (PMC11008031; doi:10.1186/s13100-024-00318-9)
Supplement: Supplementary file 2 — Supplementary Material 2 [file 13100_2024_318_MOESM2_ESM.pdf]

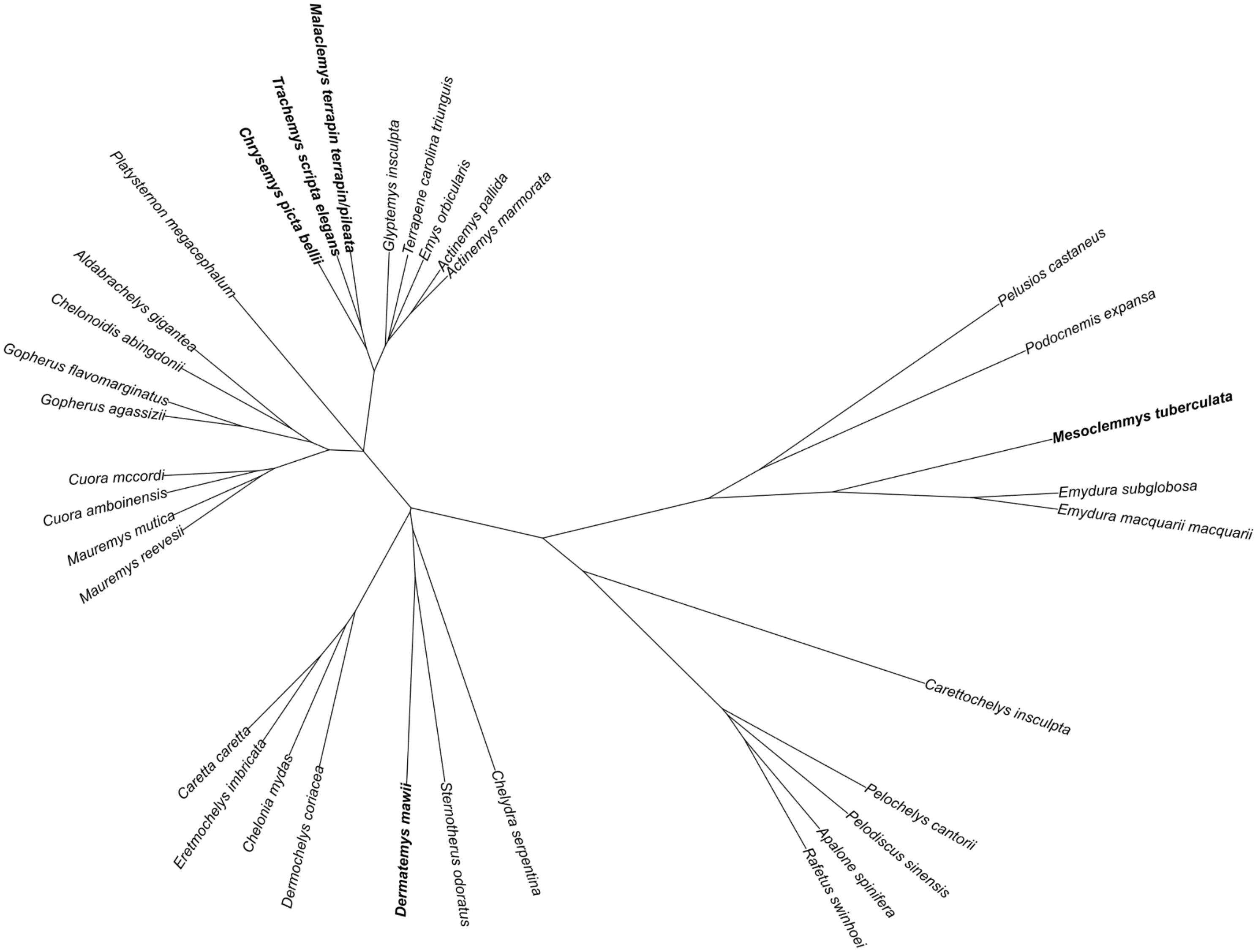

Figure S1: Phylogeny of Testudine genomes available on NCBI (https://www.ncbi.nlm.nih.gov/assembly search=Turtle) made with Timetree (see Methods). Species in bold text contain hAT-6_XT DNA transposable element.

```
Etheostoma_spectabile             -----------MAK-----RKMYSENRIFQSRWENEYMFTEIAGKLVCLLCGSNVAVMKE
Malaclemys_terrapin_terrapin      ------------------------------------------------------------
Trachemys_scripta_elegans         -----------MAK-----RKIDSENRGFQSRWENEYMFTEIAGKPVCLLCGSNIAVMKE
Scophthalmus_maximus              -----------MAK-----RKVDSENRAFQNRWEAEYMFTDIAGKPVCLVCGANVAVIKE
Syngnathus_acus                   -----------MPK-----RKVDSENRAFKNRWEVEYMFTDIAGKPVCLICGANVAVLKE
Thalassophryne_amazonica          -----------MPK-----RKVDSENRAFKSRWEAEYMFTDIAGKPVCLICGDNVAVIKE
Xenopus_tropicalis                -----------MPK-----RKVDSKNRAFKNRWEAEYMFTDIAGKPLCLICGANVAVIKE
Scleropages_formosus              MLQVIAKLALTMPK-----RKVDSENRAFKNRWEAEYMFTDIAGKPVCLICGANVAVIKE

Etheostoma_spectabile             YNLRRHYETKHEDKLKNLSAGQKLQKVEELKKNLTSQQTFFTKAKSQSEAAVKASFIVAE
Malaclemys_terrapin_terrapin      ------------------------------------------------------------
Trachemys_scripta_elegans         YNLRRHYETKHENKFKNLSAGQKLQKVEELKKNLTSQQTFFTKAKSQSEAAVKASFIVAE
Scophthalmus_maximus              FNIRRHYETKHQE-LQNLNAEEKIQRVKELKKNLRFQQTFFTRAKSQSEAAVKASFIVAQ
Syngnathus_acus                   FNLRRHYETKHLDNLKDLNAEQKIQKVEELKKKLTFQQTFFTRAKSQSEAAVKASFIVAE
Thalassophryne_amazonica          FNLRRHCETKHQDNLKDLNAEQKIQKAEDLKKNLTLQQTFFTRAKSQSEAAVKSSFIAAE
Xenopus_tropicalis                FNLRRHYETKHQDNLKDLNAEQKIQKVEELKKNLTLQQTIFTRAKSESEAAVKASFIVAE
Scleropages_formosus              FDLRRHYETKHQDNLKDLNAEQKIQKAEELKKNLTLQQMFFTRAKSQSEAAVKASFIVAE

Etheostoma_spectabile             EIAKSGRPFTEGEFVKNCMMKVCDVVCPDKTRAFANVSLSRNTVANRVCEMATDLKTQLI
Malaclemys_terrapin_terrapin      --------------------------------------------------MATDLKTQLI
Trachemys_scripta_elegans         EIAKSGRPFTEGEFVKNCMMKVCDVLCPDKTRAFANVSLSRNTVANRVCEMATDLKTQLI
Scophthalmus_maximus              EIAKSARPFTEGEFLKSCMMKVCDVLCPENKQMFANVSLSRNTVADRICEMATDLKTQLS
Syngnathus_acus                   EIAKAGRPFTEGEFLKSCMVKVCDIICPDKKQMLANVSLSRNTVADRVCEMATDLRTQLS
Thalassophryne_amazonica          EIAKSARPFTEGEFLKSCMIKVFDVLCPDKKQILANVSLSRKMIADRVCEMATDLRTQLS
Xenopus_tropicalis                EIAKSARPFTKGEFLKSCMIKVFDVLCPDKKQMLAN------------------------
Scleropages_formosus              EVAKSARPFTEGEFLKSCMIKVFDVLCPDKKQMLANVSLSRNTIADRVREMATDLRTQLS

Etheostoma_spectabile             ERAKDFVAYSLAVDETTDSTDTAQLAIFIRGVDSNLCVTEEILDIKSMHGTTKGEDIFGN
Malaclemys_terrapin_terrapin      ERAKDFVAYSLAVDETTDATDTAQLAIFIRGVDSNLCVTQEILDIKSMHGTTKGEDIFGN
Trachemys_scripta_elegans         ERAKDFVAYSLAVDETTDATDTAQLAIFIRGVDSNLCVTEEILDIKSMHGTTKGEDIFGN
Scophthalmus_maximus              ERSKDFTAFSLAVDESTDMTDTAQLAIFIREVDSSLCVTEEILDIKSMHGTTTGKDIFEN
Syngnathus_acus                   KRSKDFIAYSLAMDESTDMTDTAELAIFIRGVDSDLRVT------------------FEN
Thalassophryne_amazonica          ERSKDFIAYSLAVDESTDMTDTAQLAIFIHGVDSNLRVTEEIMDIKLMHGTTTGKDIFEN
Xenopus_tropicalis                ------------------------------------------------------------
Scleropages_formosus              ERSKDFIAYSLAVDESTDMTDTAQLAIFIRGVDSNLRVTEEIMDIKSMHGTTTGKDIFEN
                                               *

Etheostoma_spectabile             VFQSVTDMRLPWEKLVGLTTDGAPAMCGEKKWTGGKDALKDAGGELCR------------
Malaclemys_terrapin_terrapin      VFQSVTDMKLPWEKLIGLTTDGAPAMCGEKNGLVGRMRSKMRE-ENCA------------
Trachemys_scripta_elegans         VFQSVTDMKLPWEKLVGLTTDGAPAMCGEKNGLVGRMRSKMRE-ENCAGELTVYHCIIHQ
Scophthalmus_maximus              VCQSITDMKLPWDKLIGLTTDGAPAMCSEKVGLVGRMRAKMQE-ENCTGELTAYHCIIHQ
Syngnathus_acus                   VCQSVTDMKLPWDKLIGLTTDGAPAMCGEKSGLVGRMREKMQE-ENCTGELTTYHCIIHQ
Thalassophryne_amazonica          VCQSITDMKLPWDKRIALTTDGAPSMCSEKRGLVGRMRVKMQ--ENCT------------
Xenopus_tropicalis                ------------------------------------------------------------
Scleropages_formosus              VCQSITDMKLPWDKLIALTTDGAPSMCSEKSGLVGRMRVKMQE-ENCTGELTAYHCIIHQ
                                                       *

Etheostoma_spectabile             -------------------------AHGLNHRQFQSFLREIDCEFGDMPYHTEVRWLSRG
Malaclemys_terrapin_terrapin      -------------------------EPRLNHRQFQSFLREIDSEFGDMPYHTEVRWLSRG
Trachemys_scripta_elegans         ESLSAKVLKMDHVMNTVTQTVNFIRAHGLNHRQFQSFLREIDSEFGDMPYHTEVRWLSRG
Scophthalmus_maximus              EMLCCKVLKMEHVMNTVTQTVNFIRAKGLNHWQFQSFMREIDSEFADIPYHTEVRWLSRG
Syngnathus_acus                   EALCGKVLKMDHVMSTVTQTVNFIRSRGLNHRQFQSFMRTIDSEFADIPYHTEVRWLSRG
Thalassophryne_amazonica          ----------------------------------------------------EVRWLSWG
```

```
Xenopus_tropicalis                ------------------------------------------------------------
Scleropages_formosus              EALCGKVLKMDNVMSTLTQTVNFIRAKGLNHRQFQSFMREIDSEFADIPYHTEVRWLSRG
                                                                                        **

Etheostoma_spectabile             KVLKRHFELREEICQFMDSKGKDCTVLRDEKWKCELAFLADITSHLSALNLQLQGREHII
Malaclemys_terrapin_terrapin      KVLKRHFELREEICQFMDSKGKDCTVLRDEKWKCELVFLADITSHLSALNLQLQGREHII
Trachemys_scripta_elegans         KVLKRHFELREEICQFMDSKGKDCTVLRDEKWKCELAFLADITSHLSALNLQLQGREHII
Scophthalmus_maximus              KVLNRVFELSKEICQFMDSKGKDTTVLRDEKWKCELAFLADVTAHLNVLNLHLQGRDRII
Syngnathus_acus                   KVLNRVFELSNEICQFMDSKGKDSTVLRDEKWKCELAFLADITAHLNALNLQLQGRDRMI
Thalassophryne_amazonica          KVLSRVFELSNKICQFMDSKGKDSTNFRDEKWKCKLAFLADITAHLNALNLQLQGRDRMI
Xenopus_tropicalis                ------------------------------------------------------------
Scleropages_formosus              KVLDRVFELSNEICQFIDSKGKDSTNFRDEKWKCELAFLADITAHLNALNLQLQGRDRMI

Etheostoma_spectabile             TDMHDAVKAFQVKLRLWETQMHQCNLSHFPCCQVILNQESDTVFPNATFAEKLSALRTEF
Malaclemys_terrapin_terrapin      TDMHDAVKAFQVKLRLWETHMHQCNLSHFPCCQVIRNQESATVFPNATFAEKLSALRTEF
Trachemys_scripta_elegans         TDMHDAVKAFQVKLRLWETQMHQCNLSHFPCCQVIRNQESATVFPNATFAEKLSALRTEF
Scophthalmus_maximus              TDMYDTVKAFKVKLLLWETQMRQSNLPHFPCCQVMFNQVGATVFPNTHFADKLSALRTEF
Syngnathus_acus                   TDMYDAVKAFQVKLILWETQMLQCNLSHFPCCQVMLNQVGTTVFPNTHFAVKLSALRTEF
Thalassophryne_amazonica          TDMYDAVKAFQVKLLLWESQMHQCNMSHSPCCQVMLNQAGTTVFPNMHFADKLSALRTEF
Xenopus_tropicalis                ------------------------------------------------------------
Scleropages_formosus              TDMYDAVKAFQVKLLLWETQMRQCNLPHFPCCQVMLNQVGTTVFPNTHFADKLSAPRTEF

Etheostoma_spectabile             ARRFSDFEAQKSNFELLRNPFAVDVETAPVEMQMELIELQCNGTLKAKYDTEGPAQFTRF
Malaclemys_terrapin_terrapin      ARRFSDFEAQKSNFELLRNPFAVDVETAPVEMQMELIELQCNGTLKAKYDTAGPAQFTRF
Trachemys_scripta_elegans         ARRFSDFEAQKSNFELLRNPFAVDVETAPVEMQMELIELQCNGTLKAKYDTAGPAQFTRF
Scophthalmus_maximus              ARRFGDF----------RNPFAVDVETAPVQIQMELIELQCNGTLKAKYDTAGPAQFIRS
Syngnathus_acus                   ARRFGDFEEQKKNFELFRNPFAVDVESAPVQIQMELIELQCNGTLKSKYDTAGPTEFIHS
Thalassophryne_amazonica          ARRFGDFEEQKKNLELLRNPFAVDVETAPVQIQMKLIELQCNGTLKAKYDTAGPPQFIHA
Xenopus_tropicalis                -------------------------------IQMELIELQCNGTLKAKYDTAGPAQFIHS
Scleropages_formosus              ARRFGDFEEQKKNFEFLRNPFAVDVETAPVQIQMELIELQCNGTLKAKYDTAGPAQFIHS

Etheostoma_spectabile             L--PEAMPQLRQHAARILSMFGSTYSGAQKFTYTCLS-----------------------
Malaclemys_terrapin_terrapin      I--PEAMQQLRQHAARILSMFGSTYLCEQLFSVMKINKTSHRSRLTDEHLQSILRIFTTQ
Trachemys_scripta_elegans         I--PEAMPQLRQHAARILSMFGSTYLCEQLFSVMKINKTSHRSRLTDEHLQSILRIFTTQ
Scophthalmus_maximus              I--PETMPQLRLHAAQTLCMFGSTYLCEKLFSVMKMNKTAHRSRLTDGHLQSILRISTAQ
Syngnathus_acus                   I--PAAMSQLRLHVARTLCMFGSTYLCEKLFSVMKTNKTAHRSRLTDEHLQSILRVSTTR
Thalassophryne_amazonica          I--PAEMPQLRLHVARTLCMFGSTYLCEKLFSVMKTNKTAHRSHLTDEHLQSILRISTTQ
Xenopus_tropicalis                I--PAEMPQLRLHAARTLCMFGSTYLCEKLLSVMKTNKTAHRRHLTDEHLQSILRIS-TQ
Scleropages_formosus              I--PAEMPQLRLHAARTLCMFGSTYLCEKLFSVMKTNKTAHGSRLTDEHLQSILRISTTQ
                                                             *
Etheostoma_spectabile             ------------------------
Malaclemys_terrapin_terrapin      NLTPNINELVAKKRLQVSGSD---
Trachemys_scripta_elegans         NLTPNINELVAKKRLQVSGSD---
Scophthalmus_maximus              ELTPNLNDLTAKKRCQTSCSDKMA
Syngnathus_acus                   DLTPNINQLVAKKRCQSSGSDKMA
Thalassophryne_amazonica          KLTPNIKELVAKKRCQASSFDKTT
Xenopus_tropicalis                NLTPNINNLFV---CQASSSDKMT
Scleropages_formosus              NLTPNINELVAKKRCQASSSDKMT
```

Figure S2: Conservation of the DDE catalytic triad in hAT-6_XT transposases marked by asterisk (*).

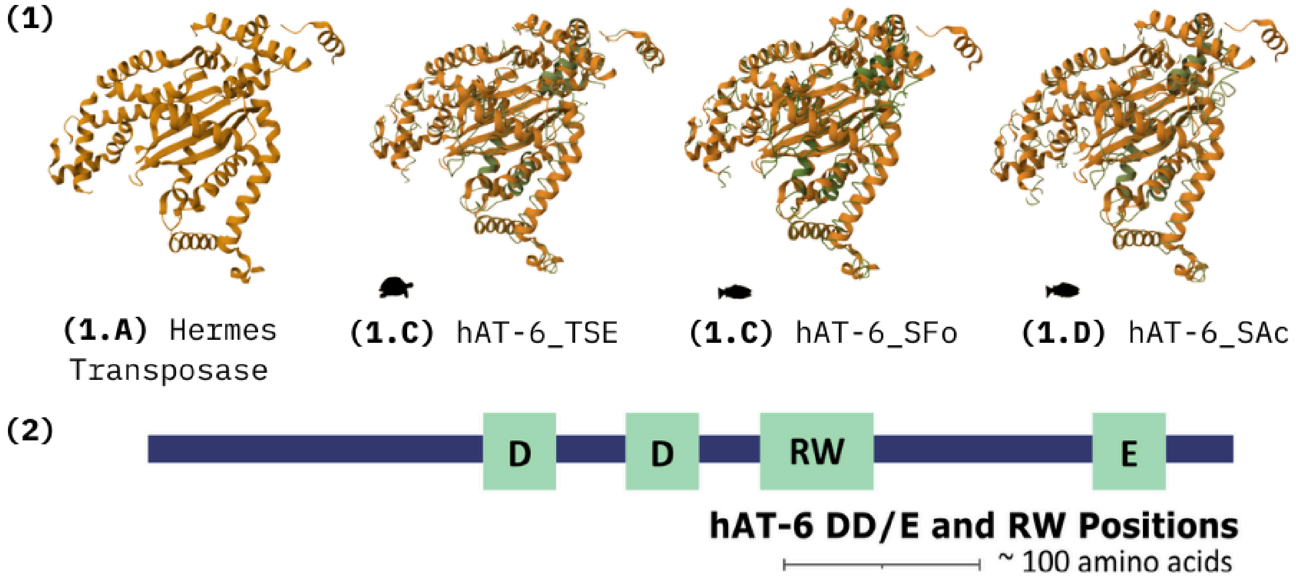

Figure S3: Predicted 3D structure of hAT-6_XTs and the essential residues required for transposition. (1) The predicted 3D structures of a sample of hAT_XT transposases. 1.A) Three-dimensional ribbon structure of the Hermes DNA transposase monomer (https://www.rcsb.org/structure/2BW3). AlphaFold structural alignment of the best-predicted ribbon structures of 1.B) hAT-6_XT_TSE 1.C) hAT-6_XT_SFo, and 1.D) hAT-6_XT_SAc is overlaid with the Hermes DNA transposase (orange), showing high structural similarity. (2) Schematic of relative DDD/E and RW residue positions in hAT-6_XT transposases.

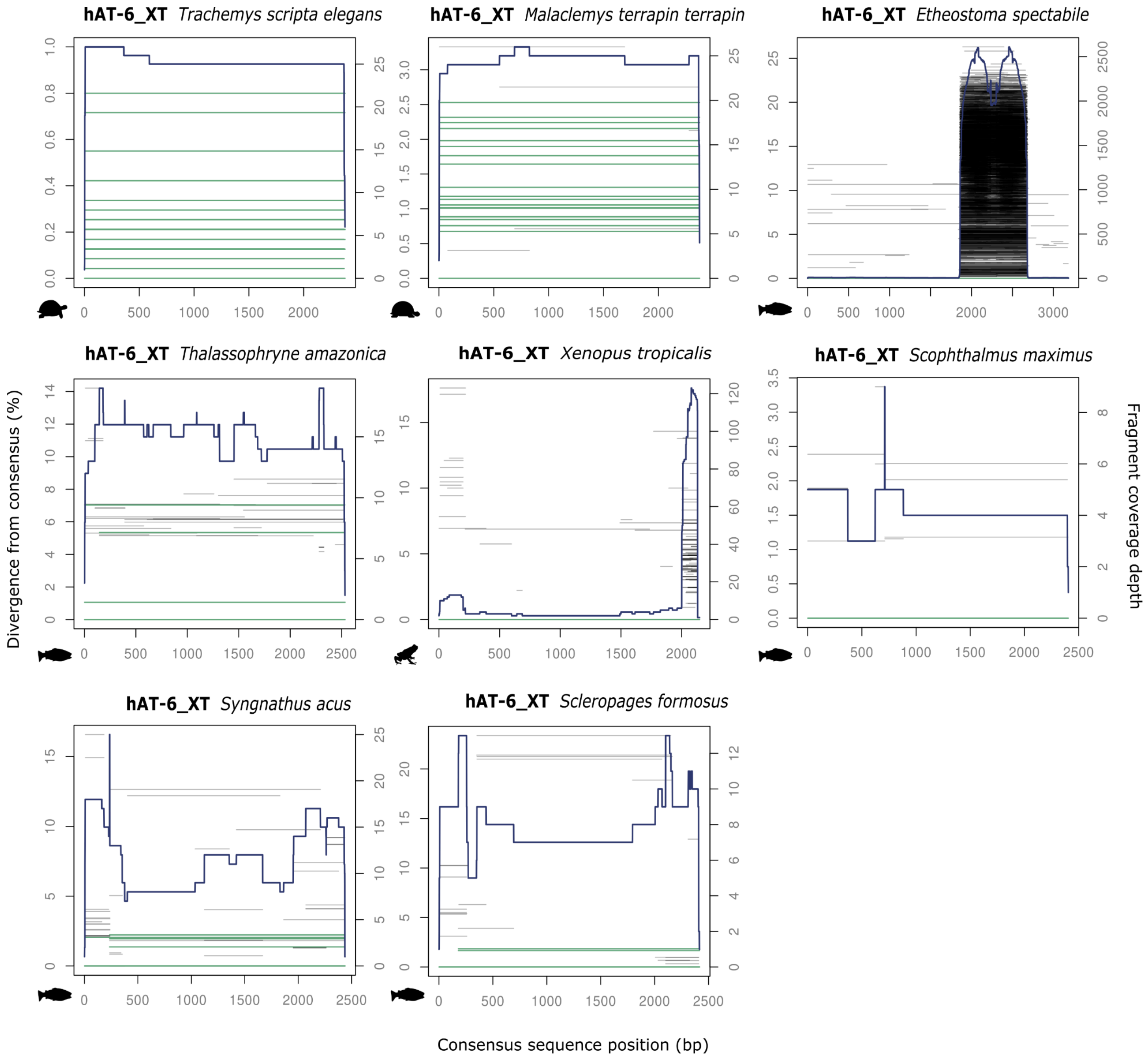

Figure S4: Coverage and divergence plots of the 8 horizontally transferred hAT-6_XT transposons. hAT-6_XT relative divergence and abundance were plotted using TE Aid (https://github.com/clemgoub/TE-Aid). The blue line represents the depth of coverage (right-hand Y-axis) of each fragment aligned to the repeat representative sequence. Green lines represent a full-length copy of the repeat. Black lines show repeat fragments. Percentage divergence from the representative sequence is shown on the left-hand Y-axis.

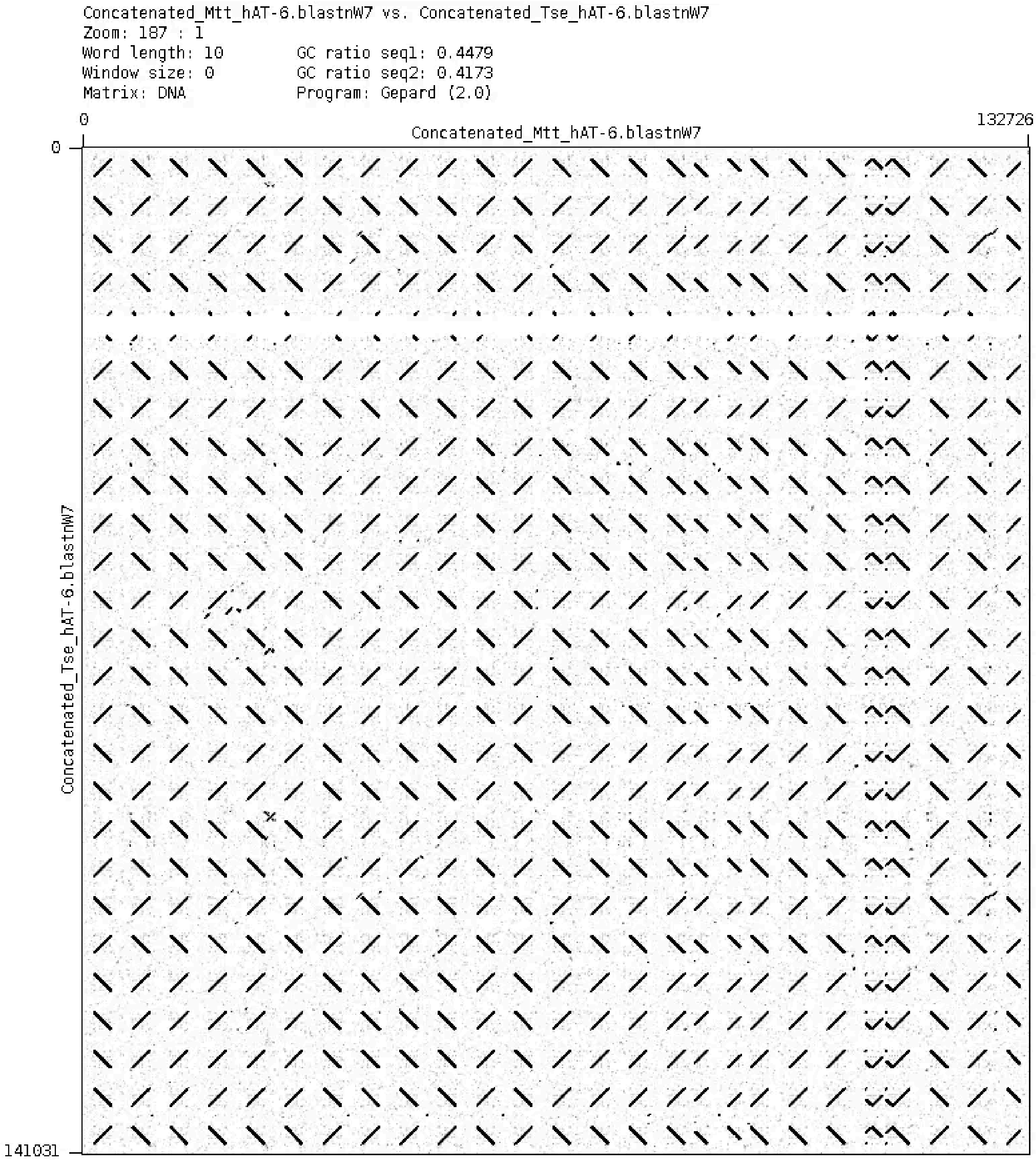

Figure S5: Dot plot alignment of all the concatenated hAT-6_XT insertions (greater than 100 bp) in hAT-6_XT_MTT vs hAT-6_XT_TSE.

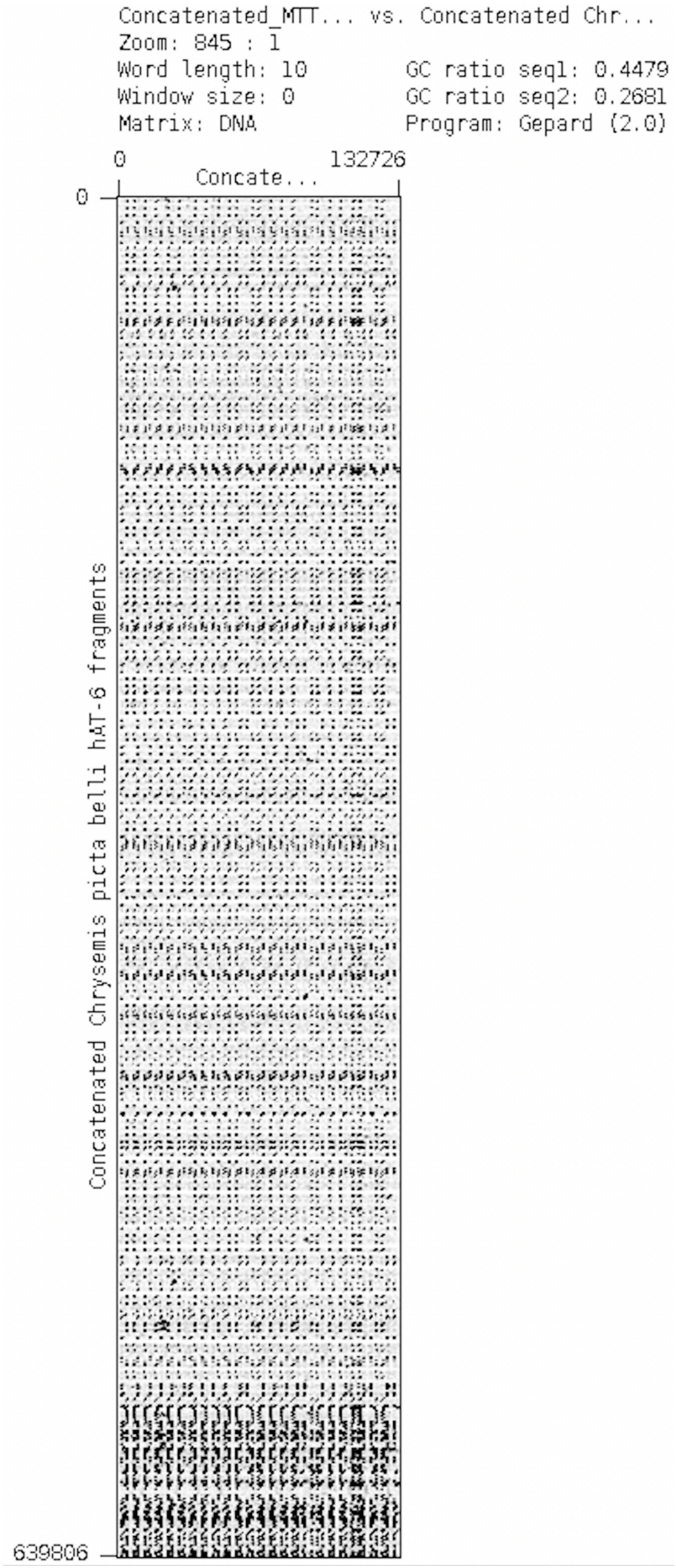

Figure S6: Dot plot alignment of all the concatenated hAT-6_XT insertions (greater than 100 bp) in hAT-6_XT_MTT vs hAT-6_XT_CPB.

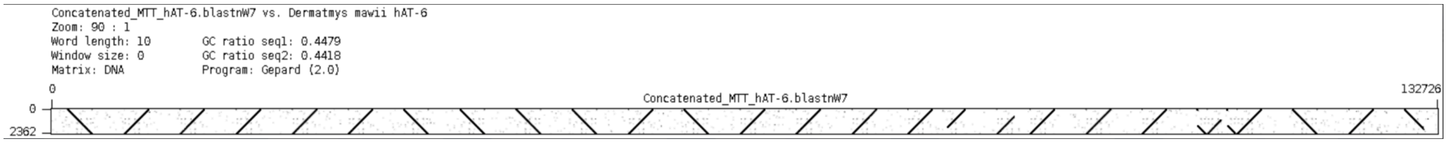

Figure S7: Dot plot alignment of all the concatenated hAT-6_XT insertions (greater than 100 bp) in hAT-6_XT_MTT vs hAT-6_XT_DMa.

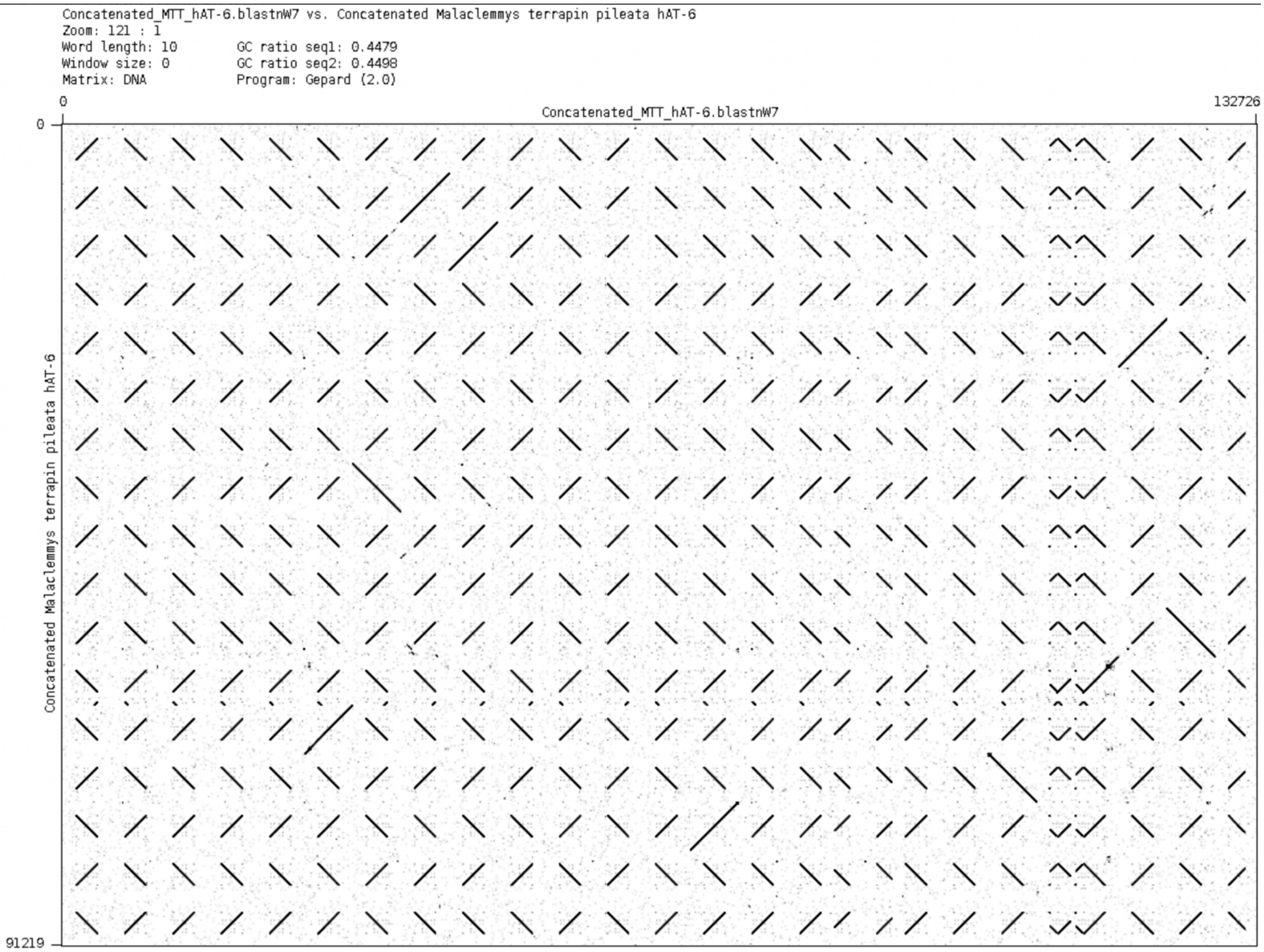

Figure S8: Dot plot alignment of all the concatenated hAT-6_XT insertions (greater than 100 bp) in hAT-6_XT_MTT vs hAT-6_XT_MTP

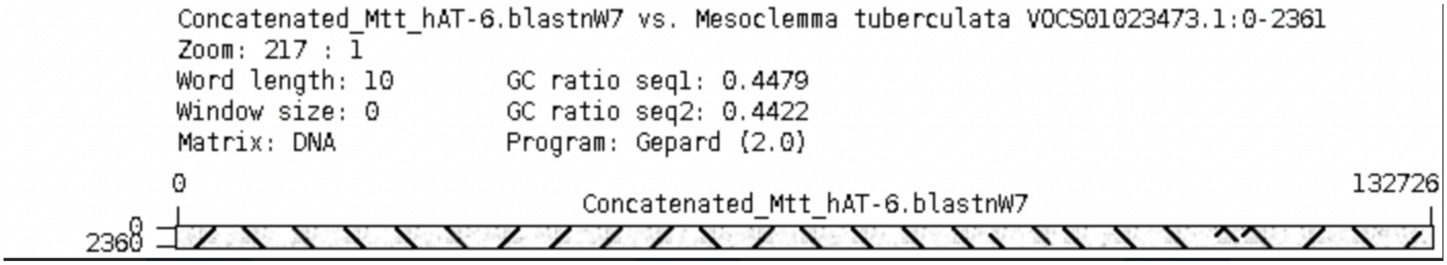

Figure S9: Dot plot alignment of all the concatenated hAT-6_XT insertions (greater than 100 bp) in hAT-6_XT_MTT vs hAT-6_XT_MTu

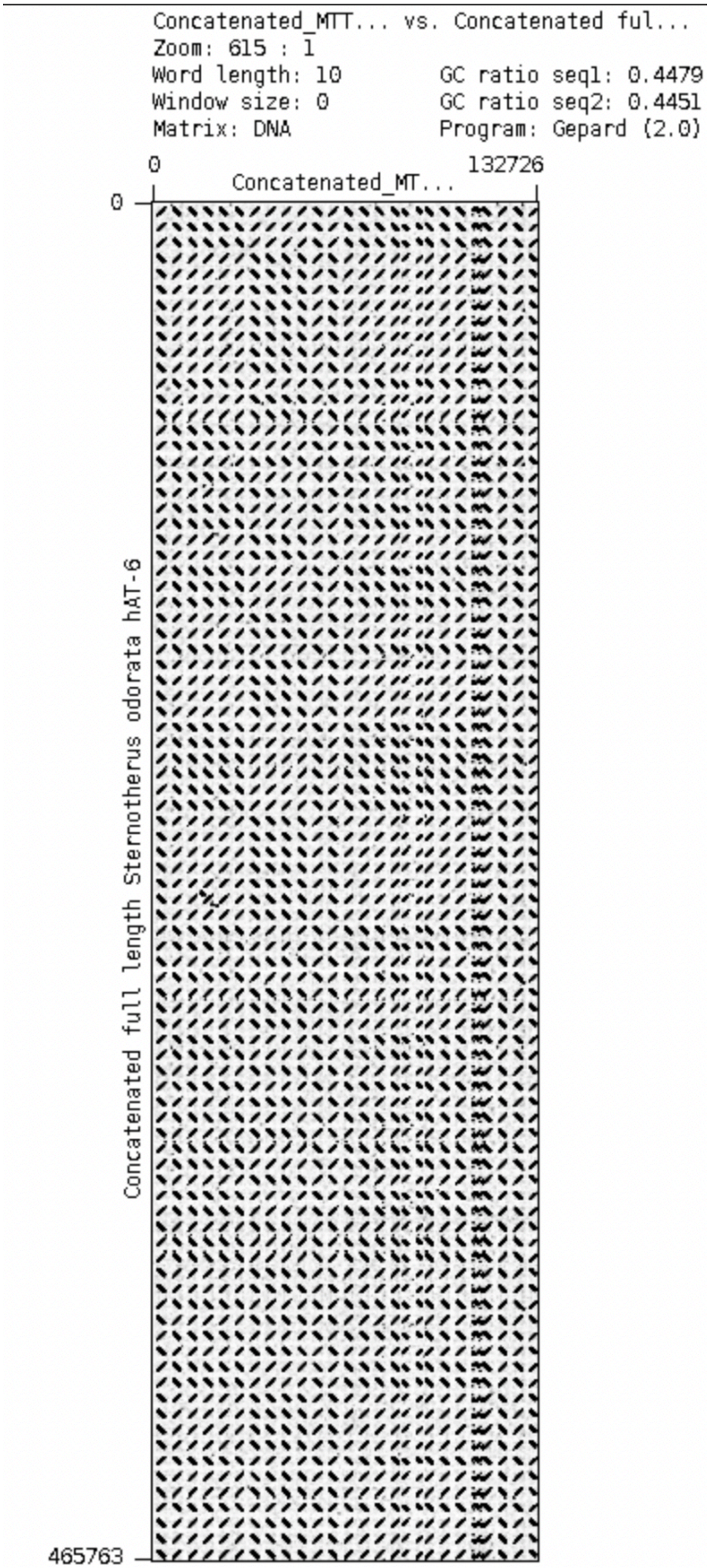

Figure S10: Dot plot alignment of all the concatenated hAT-6_XT insertions (greater than 100 bp) in hAT-6_XT_MTT vs hAT-6_XT_SOd

```
hAT-6_XT_ESp    TTCTAGACCACATGTGTCAAA--------------------------------/1860/
hAT-6N1_XT_ESp  CAGTTGTTAAGACTGGAAAAG----------------------------------/23/
                   * *   * *   *  **
hAT-6_XT_ESp    atacagtggtgctcaaaagtttacatacacatgcttaagttgactaaaaagaggaat-aa
hAT-6N1_XT_ESp  ctacagtggtgctcaaaagtttacatacacatgcttaagttgactaaaaagaggaataaa
                 ******************************************************** **

hAT-6_XT_ESp    aaaatcatgttttggaaatttattttaatacctaaattaaaaaatgaggtaaaatccaac
hAT-6N1_XT_ESp  aaaatcatgttttggaaatttattttaatacctaaattaaaaaatgaggtaaaatccaac
                ************************************************************

hAT-6_XT_ESp    ctttaaggacaccaattttctttgtgaatgaataacgtattgtaaataaataaatgttct
hAT-6N1_XT_ESp  ctttaaggacaccaattttctttgtgaatgaataacgtattgtaaataaataaatgttct
                ************************************************************

hAT-6_XT_ESp    tatttaaaatacaggggtcataagtatacatacccctatgttaaattcccatagaggcag
hAT-6N1_XT_ESp  tatttaaaatacaggggtcataagtatacatacccctatgttaaattcccatagaggcag
                ************************************************************

hAT-6_XT_ESp    gcagatttttattattaaaggccagttatttcctggattcaggatattatgcatcctgat
hAT-6N1_XT_ESp  gcagatttttattattaaaggacagttatttcctggattcaggatattatgcatcctgat
                ********************* **************************************

hAT-6_XT_ESp    aaagttcccttggcctttagaattaaaatagccccacatcctcacatactcttcaccatg
hAT-6N1_XT_ESp  aaagttcccttggcctttagaattaaaatagccccacatcatcacatactcttcaccatg
                **************************************** *******************

hAT-6_XT_ESp    cttagagataggcatggttttatttcagttagactaatacctggtttgatttgcattgag
hAT-6N1_XT_ESp  cttagagataggcatggttttatttcagttagactaatacctggtttgatttgcattgag
                ************************************************************

hAT-6_XT_ESp    agatgattttatagaaagtatcccatgcctatctctaagcatggtgaagagtatgtgagg
hAT-6N1_XT_ESp  agatgattttatagaaagtatcccatgcctatctctaagcatggtgaagagtatgtgagg
                ************************************************************

hAT-6_XT_ESp    atgtggggctattttaattctaaaggccaagggaactttatcaggatgcataatatcctg
hAT-6N1_XT_ESp  atgtggggctattttaattctaaaggccaagggaactttatcaggatgcataatatcctg
                ************************************************************

hAT-6_XT_ESp    aatccaggaaataactggcctttaataataaaaatctgcctgcctctatgggaatttaac
hAT-6N1_XT_ESp  aatccaggaaataactggcctttaataataaaaatctgcctgcctctatgggaatttaac
                ************************************************************

hAT-6_XT_ESp    ataggggtatgtatacttatgacccctgtattttaaataagaacatttatttatttacaa
hAT-6N1_XT_ESp  ataggggtatgtatacttatgacccctgtattttaaataagaacatttatttatttacaa
                ************************************************************

hAT-6_XT_ESp    tacgttattcattcacaaagaaaattggtgtccttaaaggttggattttacctcattttt
hAT-6N1_XT_ESp  tacgttattcattcacaaagaaaattggtgtccttaaaggttggattttacctcattttt
                ************************************************************

hAT-6_XT_ESp    taatttaggtattaaaataaatttccaaaacatga-tttttattcctctttttagtcaa
hAT-6N1_XT_ESp  taatttaggtattaaaataaatttccaaaacatgatttttttattcctctttttagtcaa
                *********************************** ************************

hAT-6_XT_ESp    cttaagcatgtgtatgtaaacttttgagcaccactgtatgtgtgcgagcagctgttctct
hAT-6N1_XT_ESp  cttaagcatgtgtatgtaaacttttgagcaccactgtatat-------------------

                ***************************************.*-------------------


hAT-6_XT_ESp    /2679/---------------------------------AGTTTGACACCCCTGTTCTA

hAT-6N1_XT_ESp  /1001/---------------------------------CTTTTGACAGTCCTAATGTA
                                                         *******  ***  * **
```

Figure S11: Multiple alignment of *Etheostoma spectabile* (ESp) hAT-6_XT (blue) and hAT-6N1_XT_ESp (green). Shown in bold, terminally inverted repeats (TIRs) of hAT-6_XT_ESp show homology to TIRs of hAT-6N1_XT_ESp. Conserved residues are marked by asterisk (*).

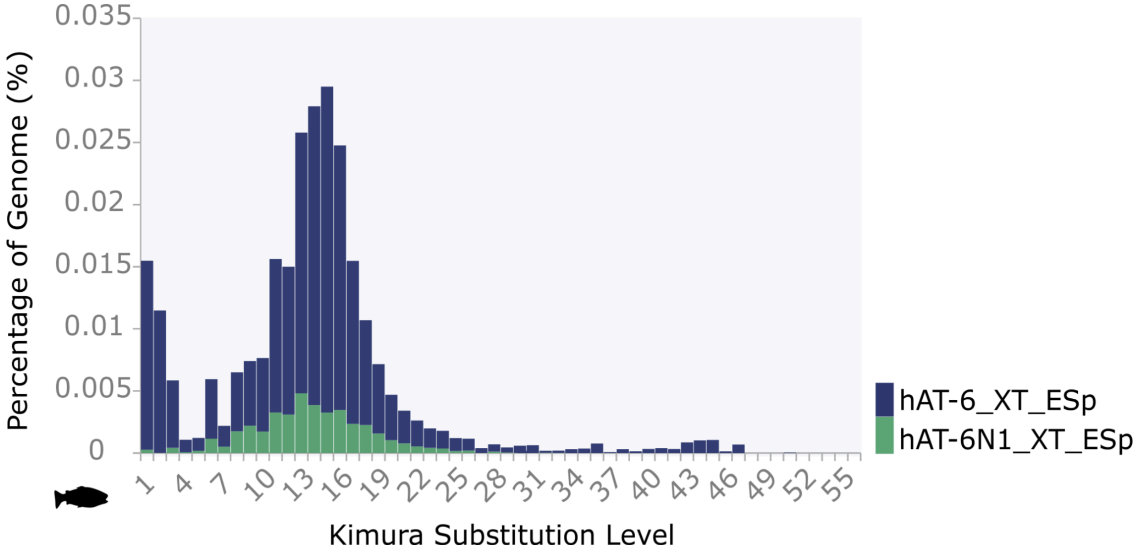

Figure S12: Kimura distance-based divergence of hAT-6_XT derived non-autonomous DNA transposon compared (hAT-6N1_XT_ESp) to hAT-6_XT from the genome of *Etheostoma spectabile* (hAT-6_XT_ESp). The left-hand axis indicates the percentage of each TE in the genome and the bottom axis displays the relative age increasing from left to right.

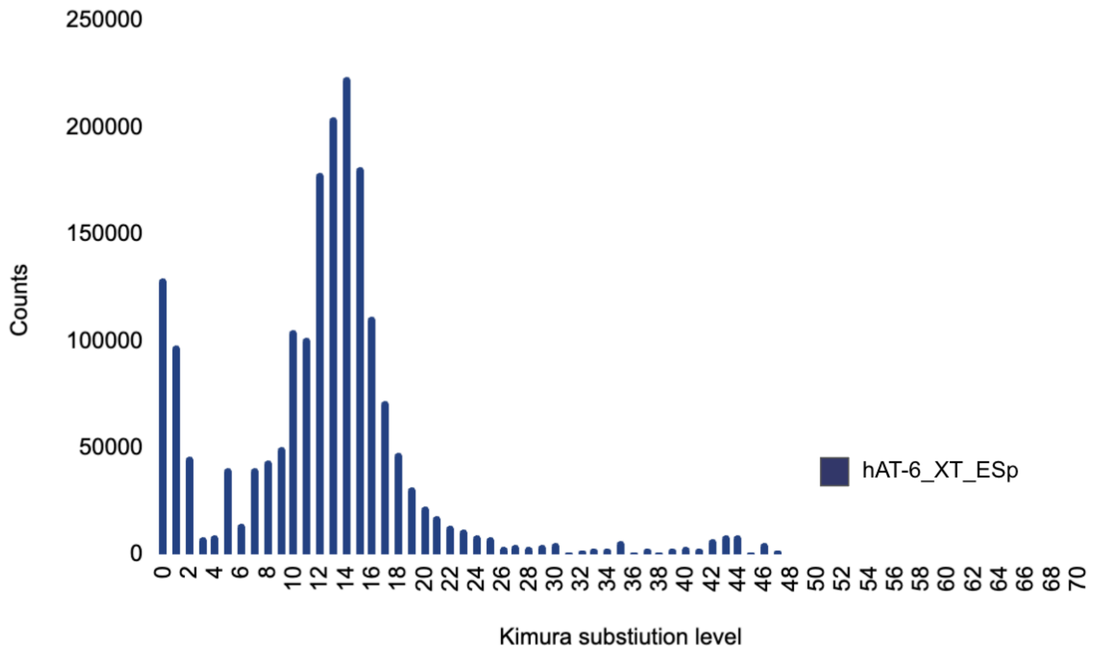

Figure S13: Kimura distance-based divergence of hAT-6_XT from the genome of *Etheostoma spectabile*. The left-hand axis for main plots and insets indicates base-pair counts of hAT-6_XT in the genome and the bottom axis for main plots and insets displays relative age shown from left to right.

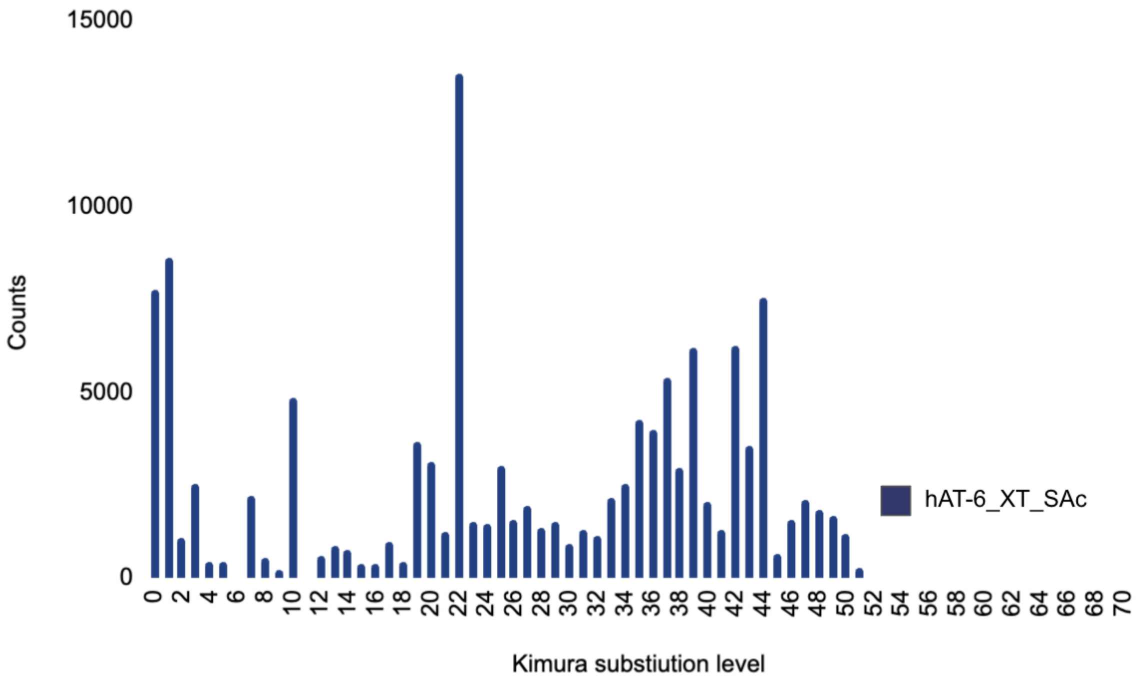

Figure S14: Kimura distance-based divergence of hAT-6_XT from the genome of *Sygnathus acus*. The left-hand axis for main plots and insets indicates base-pair counts of hAT-6_XT in the genome and the bottom axis for main plots and insets displays relative age shown from left to right.

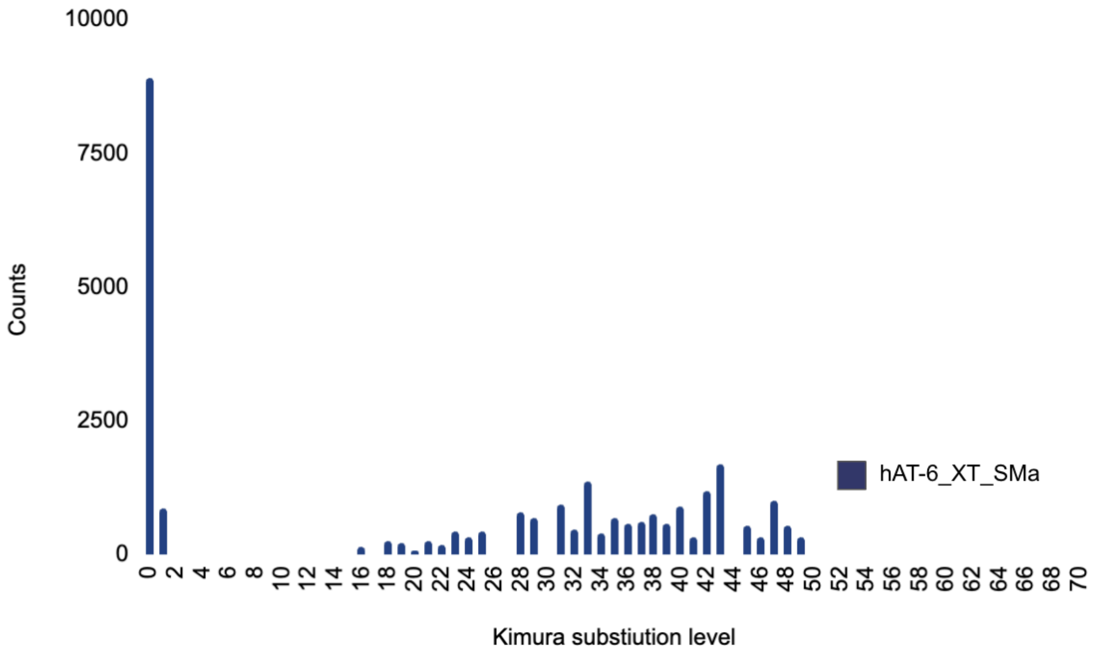

Figure S15: Kimura distance-based divergence of hAT-6_XT from the genome of *Scopthalmus maximus*. The left-hand axis for main plots and insets indicates base-pair counts of hAT-6_XT in the genome and the bottom axis for main plots and insets displays relative age shown from left to right.

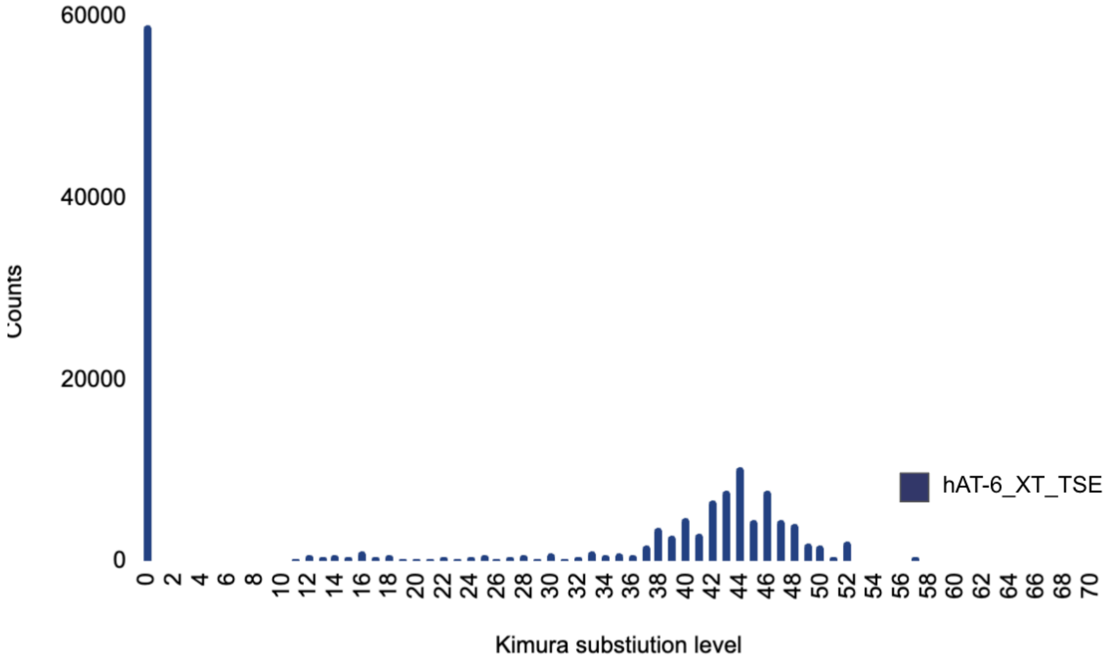

Figure S16: Kimura distance-based divergence of hAT-6_XT from the genome of *Trachemys scripta elegans.* The left-hand axis for main plots and insets indicates base-pair counts of hAT-6_XT in the genome and the bottom axis for main plots and insets displays relative age shown from left to right.

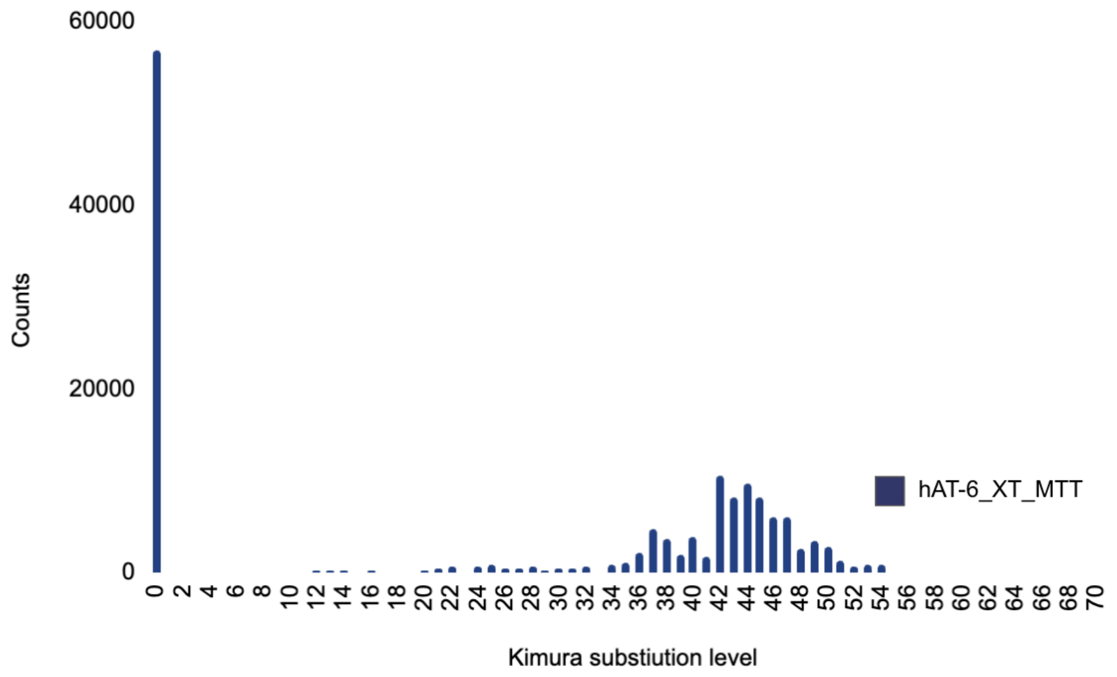

Figure S17: Kimura distance-based divergence of hAT-6_XT from the genome of *Malaclemys terrapin terrapin*. The left-hand axis for main plots and insets indicates base-pair counts of hAT-6_XT in the genome and the bottom axis for main plots and insets displays relative age shown from left to right.

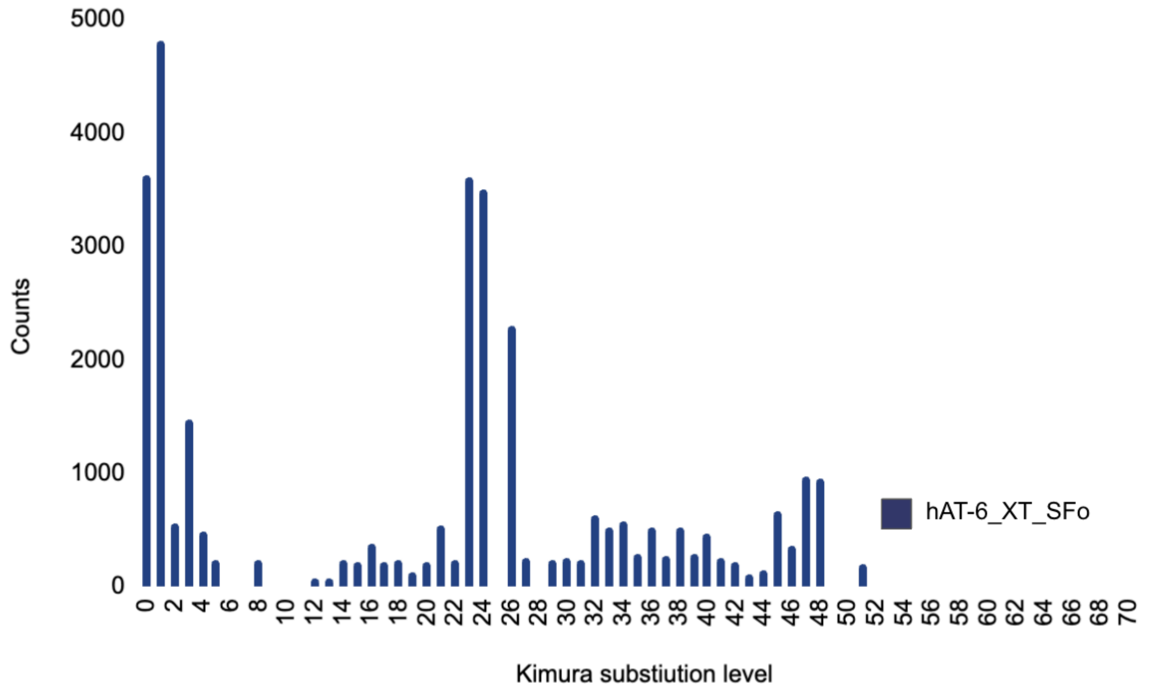

Figure S18: Kimura distance-based divergence of hAT-6_XT from the genome of *Scleropages formosus*. The left-hand axis for main plots and insets indicates base-pair counts of hAT-6_XT in the genome and the bottom axis for main plots and insets displays relative age shown from left to right.

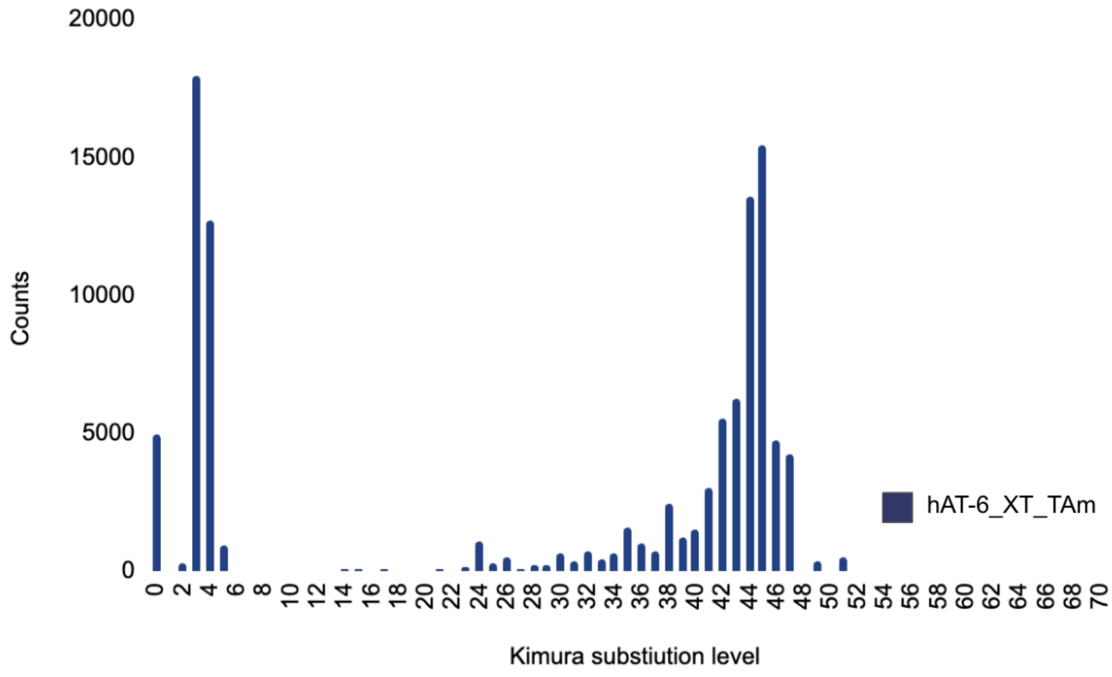

Figure S19: Kimura distance-based divergence of hAT-6_XT from the genome of *Thalassophryne amazonica*. The left-hand axis for main plots and insets indicates base-pair counts of hAT-6_XT in the genome and the bottom axis for main plots and insets displays relative age shown from left to right.

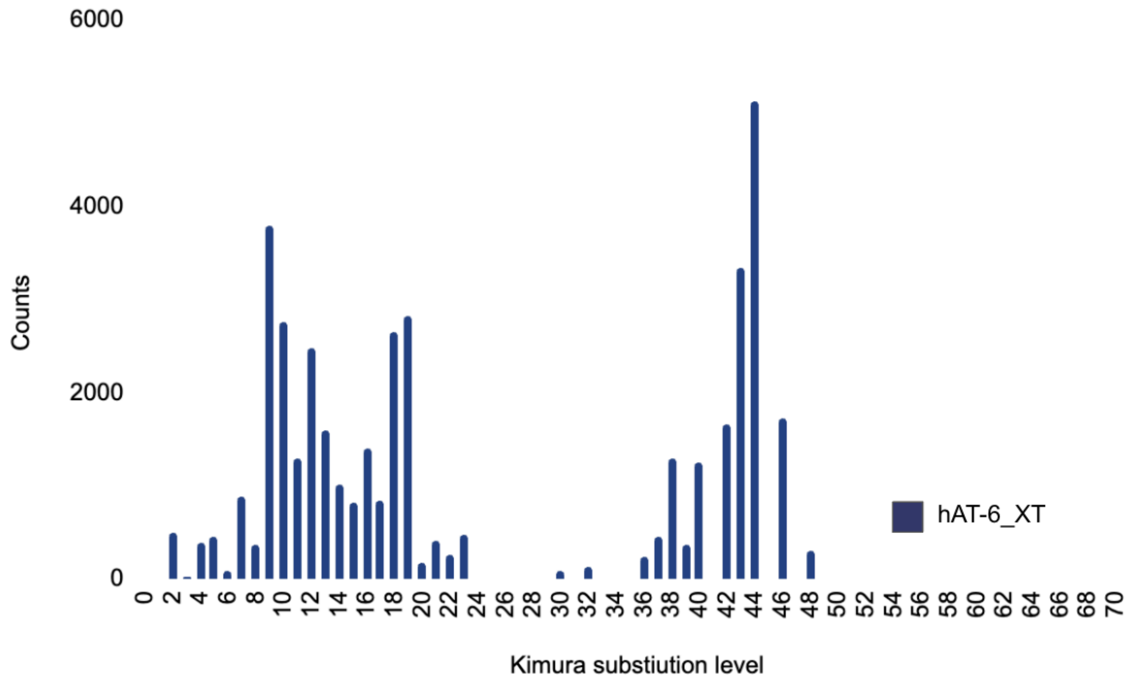

Figure S20: Kimura distance-based divergence of hAT-6_XT from the genome of *Xenopus tropicalis*. The left-hand axis for main plots and insets indicates base-pair counts of hAT-6_XT in the genome and the bottom axis for main plots and insets displays relative age shown from left to right.

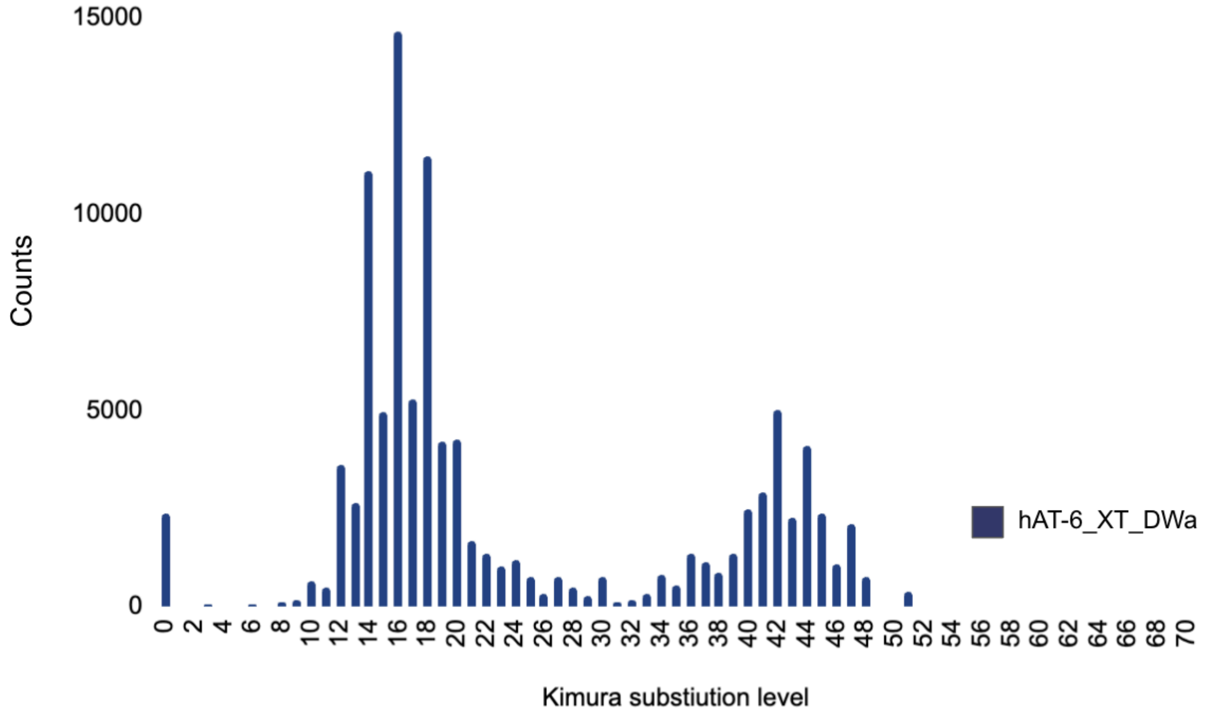

Figure S21: Kimura distance-based divergence of hAT-6_XT from the genome of *Dermatemys mawii*. The left-hand axis for main plots and insets indicates base-pair counts of hAT-6_XT in the genome and the bottom axis for main plots and insets displays relative age shown from left to right.

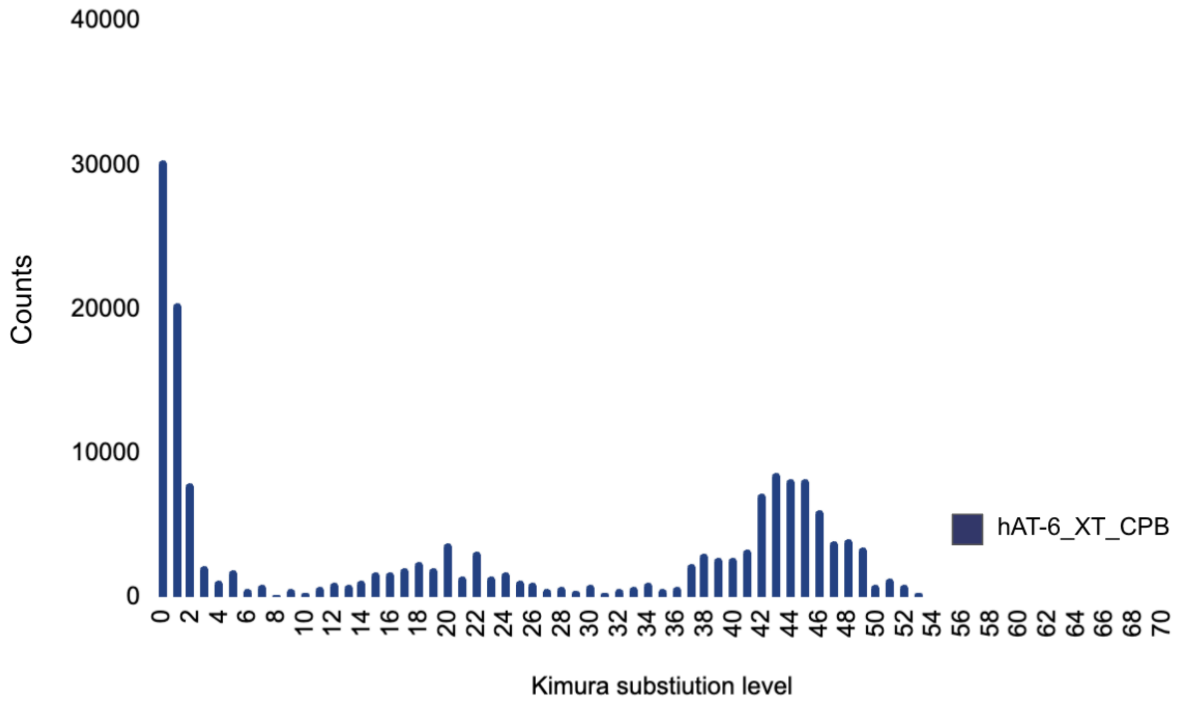

Figure S22: Kimura distance-based divergence of hAT-6_XT from the genome of *Chrysemys picta bellii*. The left-hand axis for main plots and insets indicates base-pair counts of hAT-6_X Tin the genome and the bottom axis for main plots and insets displays relative age shown from left to right.

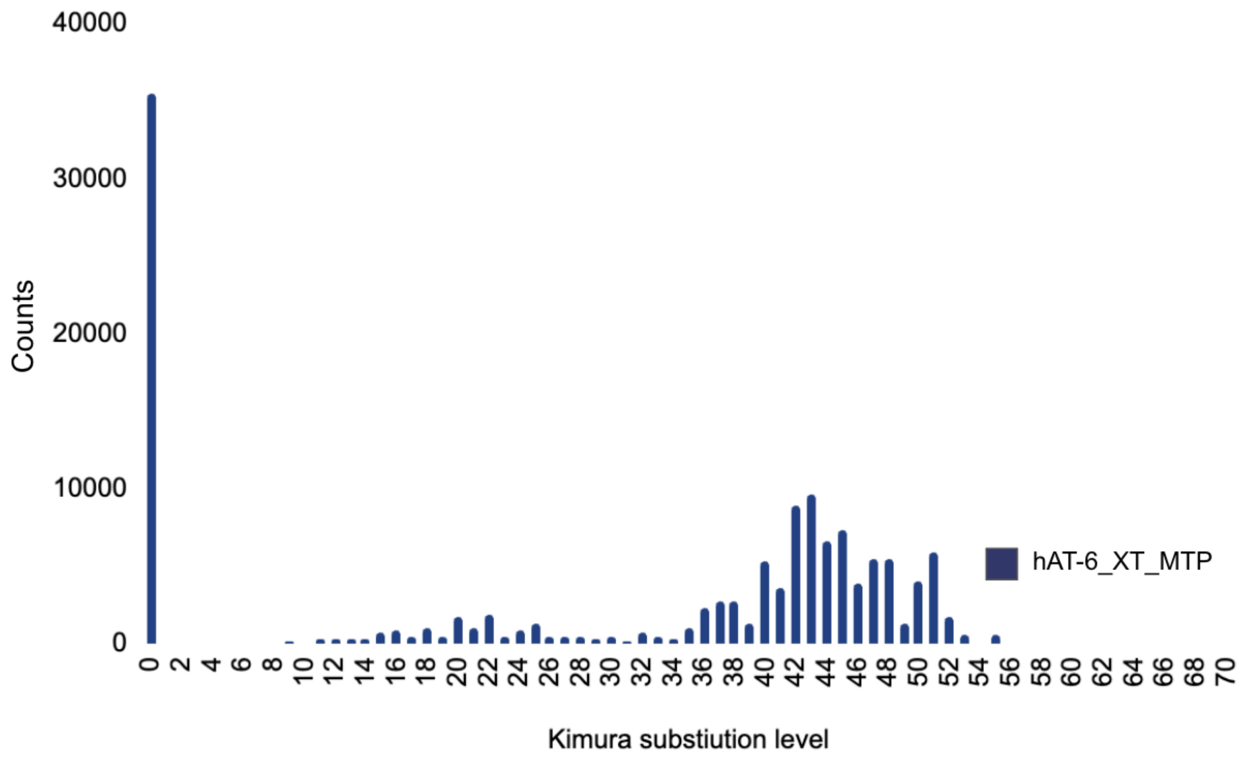

Figure S23: Kimura distance-based divergence of hAT-6_XT from the genome of *Malaclemys terrapin pileata.* The left-hand axis for main plots and insets indicates base-pair counts of hAT-6_XT in the genome and the bottom axis for main plots and insets displays relative age shown from left to right.

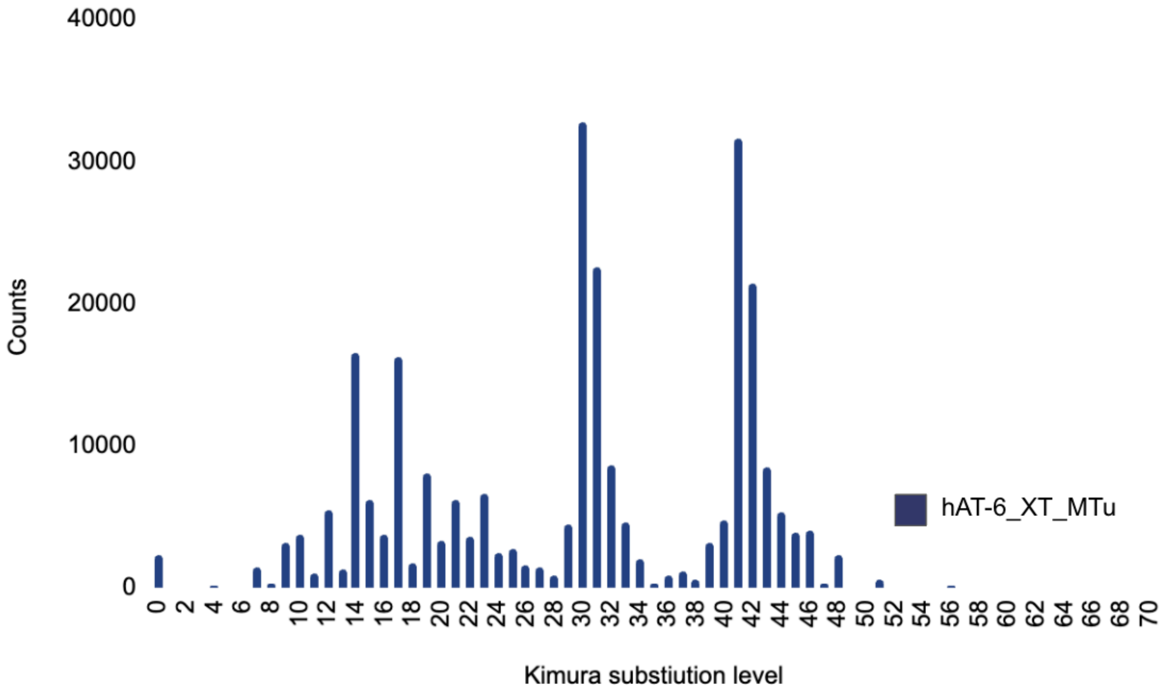

Figure S24: Kimura distance-based divergence of hAT-6_XT from the genome of *Mesoclemmys tuberculata.* The left-hand axis for main plots and insets indicates base-pair counts of hAT-6_XT in the genome and the bottom axis for main plots and insets displays relative age shown from left to right.

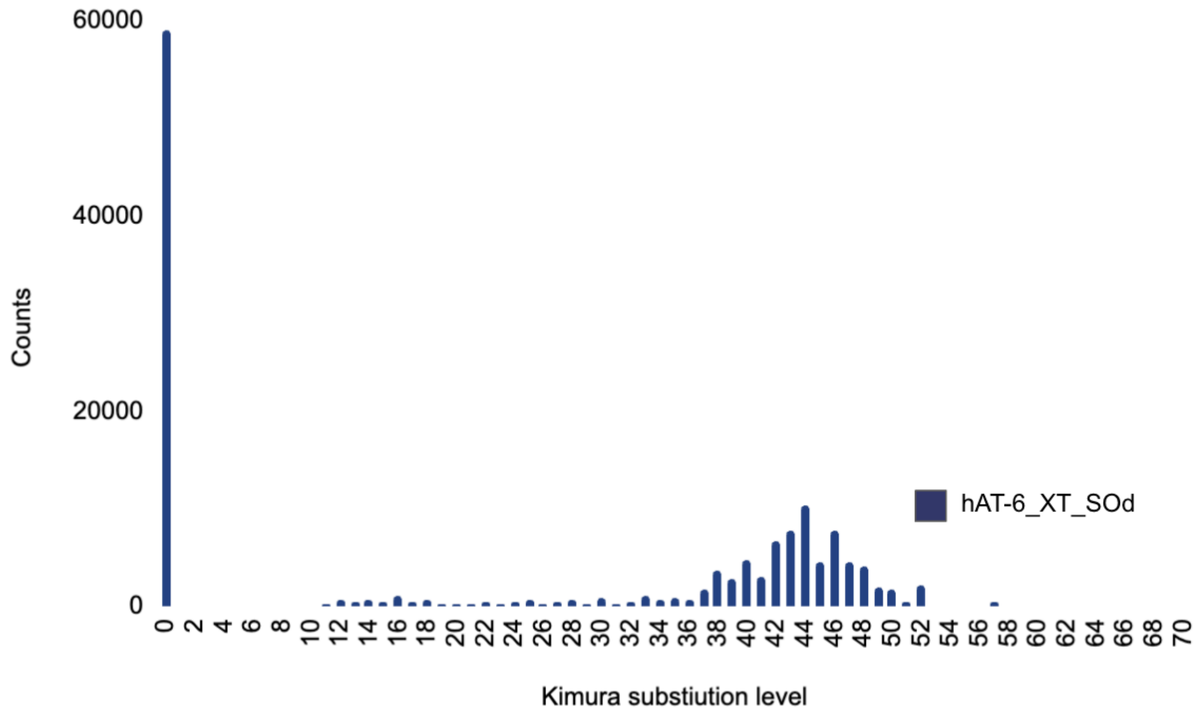

Figure S25: Kimura distance-based divergence of hAT-6_XT from the genome of *Sternotherus odoratus.* The left-hand axis for main plots and insets indicates base-pair counts of hAT-6_XT in the genome and the bottom axis for main plots and insets displays relative age shown from left to right.

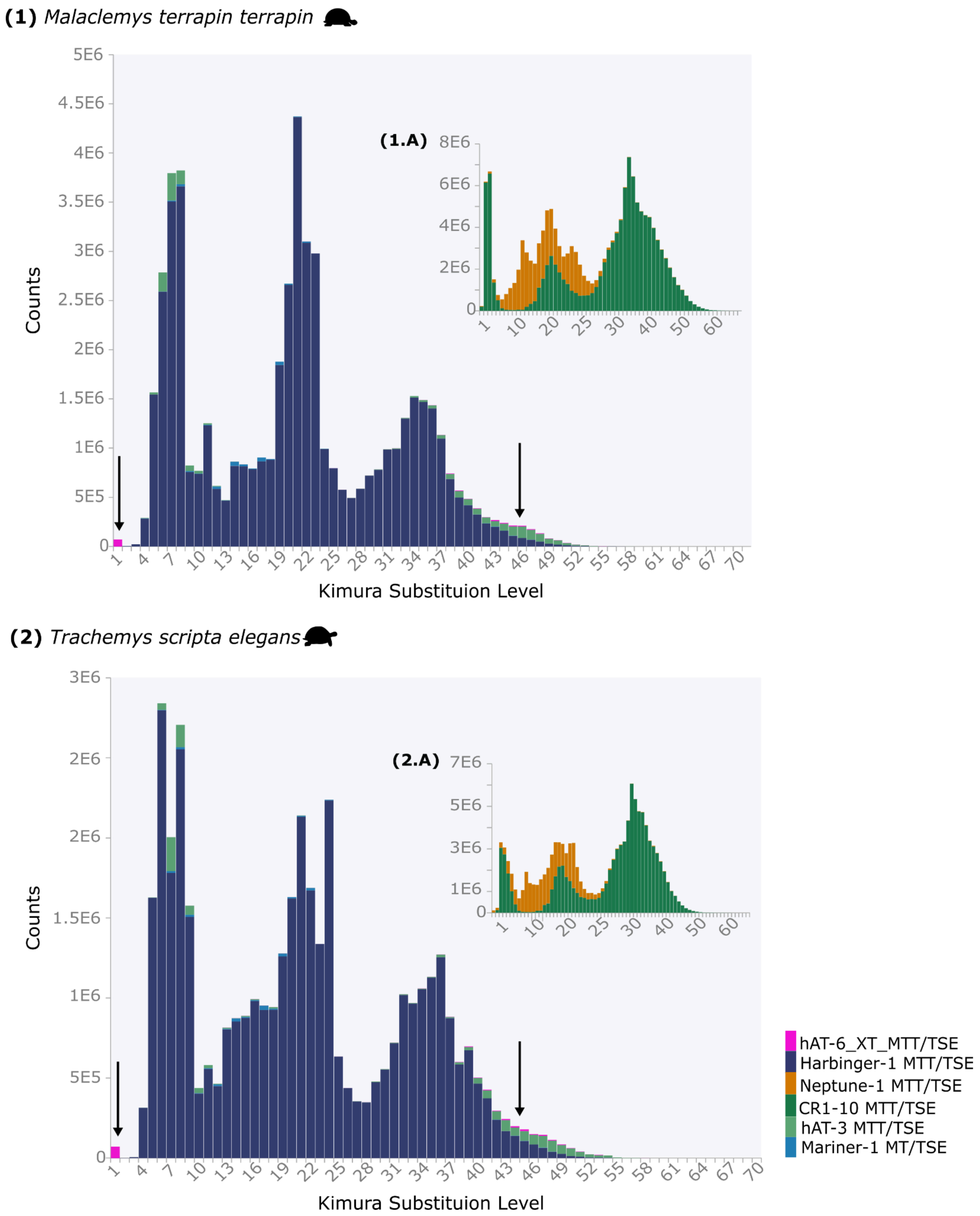

Figure S26: Kimura distance-based divergence of DNA transposons and retrotransposons from (1) *M.t. terrapin* and (2) *T.s. elegans*. DNA transposon (hAT-6_MTT/TSE, hAT-3_MTT/TSE, Mariner-1_MTT/TSE, and Harbinger-1_MTT/TSE) divergence is shown in contrast to the divergence of retrotransposons (Neptune-1_MTT/TSE and CR1-10_MTT/TSE) for both genomes. The left-hand axis for main plots and insets indicates base-pair counts of hAT-6_XT in the genome and the bottom axis for main plots and insets displays relative age shown from left to right.

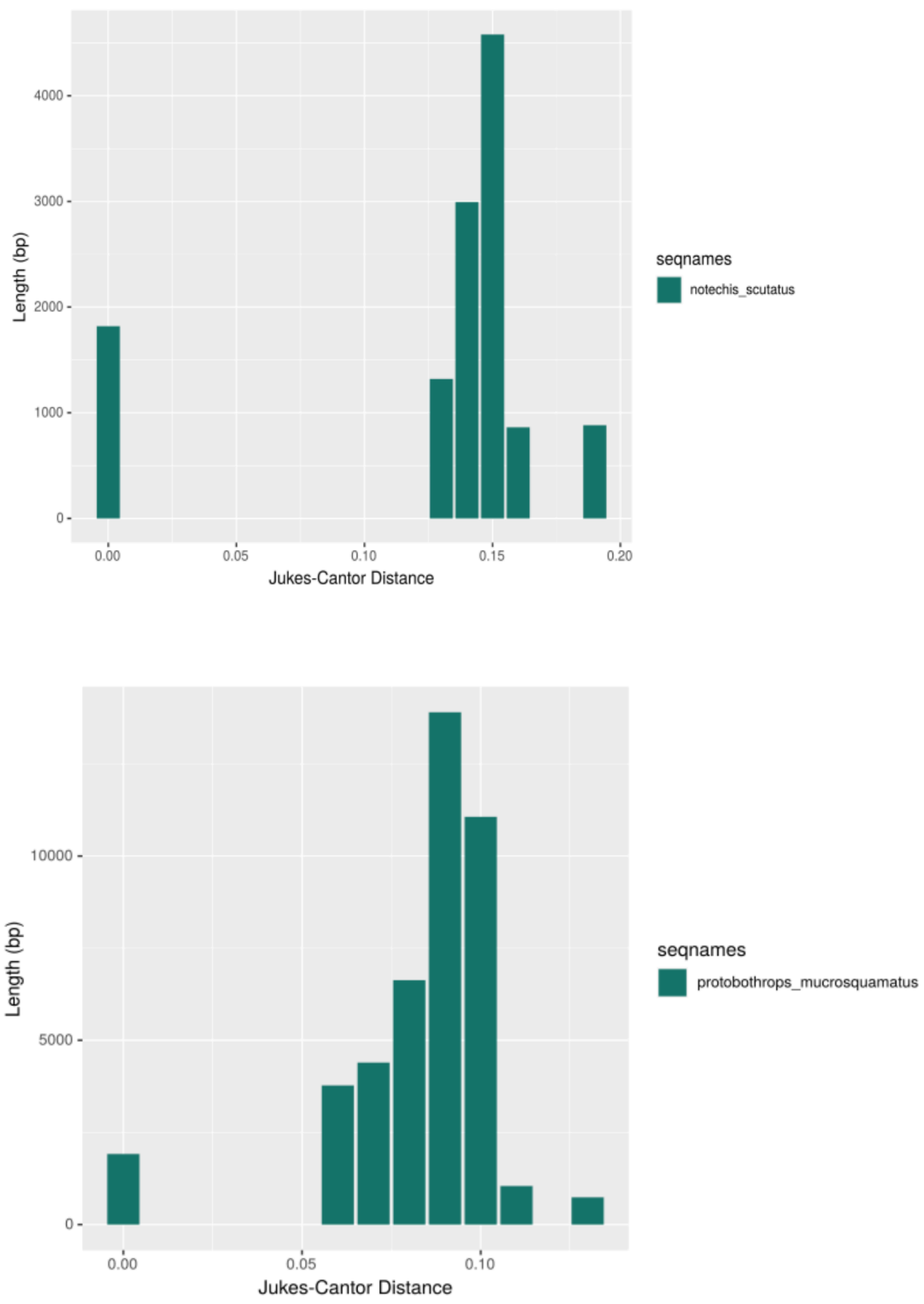

Figure S27: Divergence plots for hAT-6_XT-like sequences from snake genomes. Length of sequence (bp) is shown on the X-axis. Jukes-Cantor distance is shown in the Y axis.

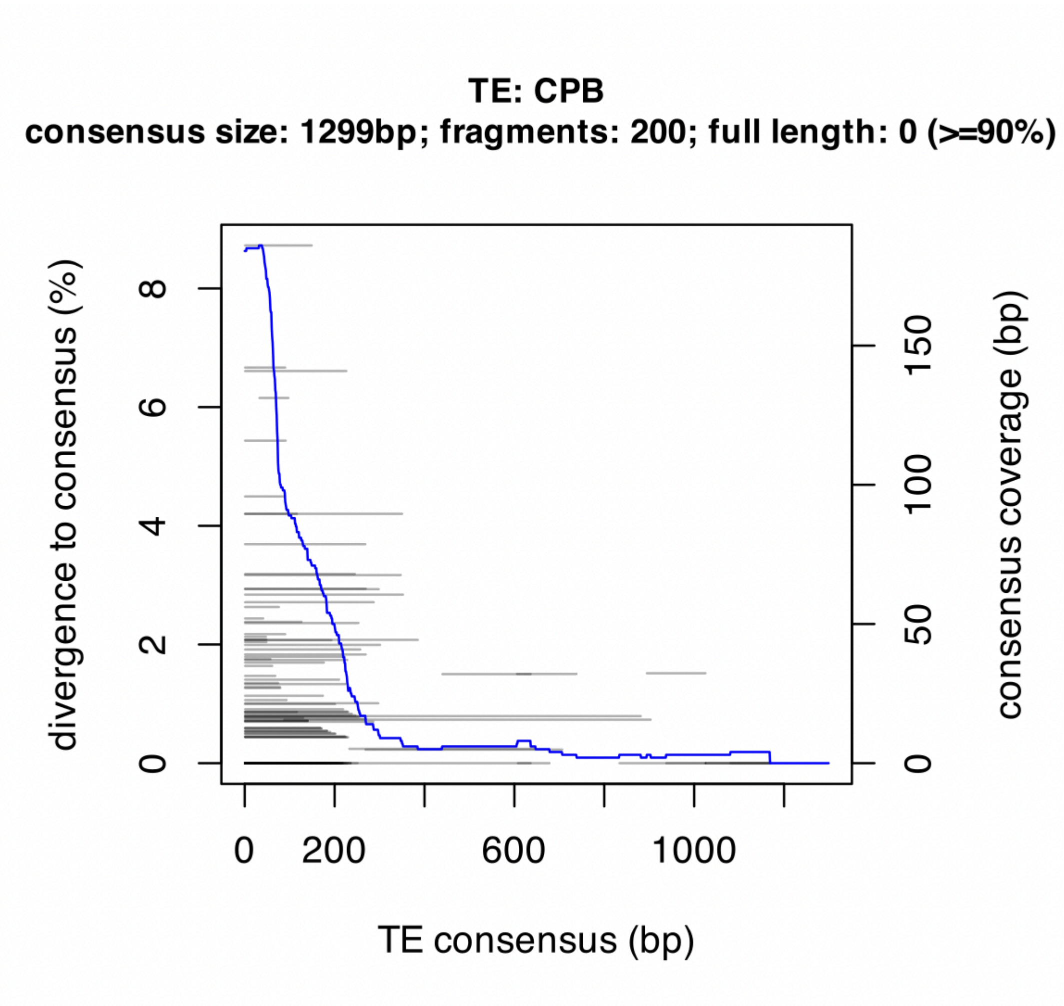

Figure S28: Coverage and divergence plots of hAT-6_XT in *C.p. bellii*. hAT-6_XT relative divergence and abundance were plotted using TE Aid (https://github.com/clemgoub/TE-Aid) [47]. The blue line represents the depth of coverage (right-hand Y-axis) of each fragment aligned to the repeat representative sequence. Green lines represent a full-length copy of the repeat. Black lines show repeat fragments. Percentage divergence from the representative sequence is shown on the left-hand Y-axis.
